# The Interaction between *30b-5p* miRNA and *MBNL1* mRNA is Involved in Vascular Smooth Muscle Cell Differentiation in Patients with Coronary Atherosclerosis

**DOI:** 10.3390/ijms21010011

**Published:** 2019-12-18

**Authors:** Chin Cheng Woo, Wenting Liu, Xiao Yun Lin, Rajkumar Dorajoo, Kee Wah Lee, A Mark Richards, Chuen Neng Lee, Thidathip Wongsurawat, Intawat Nookaew, Vitaly Sorokin

**Affiliations:** 1 Department of Surgery, Yong Loo Lin School of Medicine, National University of Singapore, Singapore 119228, Singapore; surwoocc@nus.edu.sg (C.C.W.); surlcn@nus.edu.sg (C.N.L.); 2 Genome Institute of Singapore, Agency for Science, Technology and Research (A*STAR), Singapore 138672, Singapore; liuwt@gis.a-star.edu.sg (W.L.); dorajoor@gis.a-star.edu.sg (R.D.); 3 Department of Cardiac, Thoracic and Vascular Surgery, National University Hospital, National University Health System, Singapore 119228, Singapore; xiao_yun_lin@nuhs.edu.sg; 4 Department of Pathology, Yong Loo Lin School of Medicine, National University of Singapore, Singapore 119228, Singapore; kee_wah_lee@u.nus.edu; 5 Cardiovascular Research Institute, Yong Loo Lin School of Medicine, National University of Singapore, Singapore 119228, Singapore; mdcarthu@nus.edu.sg; 6 Christchurch Heart Institute, University of Otago, Christchurch 8140, New Zealand; 7 Department of Biomedical Informatics, College of Medicine, University of Arkansas for Medical Sciences, Little Rock, AR 72205, USA; twongsurawat@uams.edu (T.W.); inookaew@uams.edu (I.N.)

**Keywords:** aortic wall, atherosclerosis, microRNA, muscle cell differentiation, vascular smooth muscle cells

## Abstract

Vascular smooth muscle cells (VSMCs) in the arterial wall have diverse functions. In pathological states, the interplay between transcripts and microRNAs (miRNAs) leads to phenotypic changes. Understanding the regulatory role of miRNAs and their target genes may reveal how VSMCs modulate the pathogenesis of coronary artery disease. Laser capture microdissection was performed on aortic wall tissues obtained from coronary artery bypass graft patients with and without recent acute myocardial infarction (MI). The mSMRT-qPCR miRNA assay platform (MiRXES, Singapore) was used to profile miRNA. The miRNA data were co-analyzed with significant mRNA transcripts. TargetScan 7.1 was applied to evaluate miRNA–mRNA interactions. The miRNA profiles of 29 patients (16 MI and 13 non-MI) were evaluated. Thirteen VSMC-related miRNAs were differentially expressed between the MI and non-MI groups. Analysis revealed seven miRNA-targeted mRNAs related to muscular tissue differentiation and proliferation. TargetScan revealed that among the VSMC-related transcripts, *MBNL1* had a recognition site that matched the *hsa-miR-30b-5p* target seed sequence. In addition to predicted analysis, our experiment in vitro with human VSMC culture confirmed that *hsa-miR-30b-5p* negatively correlated with *MBNL1*. Our data showed that overexpression of *hsa-miR-30b-5p* led to downregulation of *MBNL1* in VSMCs. This process influences VSMC proliferation and might be involved in VSMC differentiation.

## 1. Introduction

Vascular smooth muscle cells (VSMCs) are the predominant cell type within aortic tunica media [1]. These highly specialized cells possess both contractile and secretory properties, allowing VSMCs to switch from a contractile phenotype to a more synthetic phenotype in response to multiple local environmental cues [2,3,4,5].

Within the arterial wall in healthy individuals, VSMC rarely proliferate or migrate and demonstrate low synthetic activity. These cells mainly perform contractile functions supported by the expression of a series of VSMC-specific contractile proteins, including smooth muscle (SM) α-actin, SM myosin heavy chain, calponin, SM22α, and smoothelin [6,7]. In response to arterial wall injury or inflammation, VSMC have the capacity to alter their activity and function and contribute to vascular remodeling via decreased expression of contractile markers and increased VSMC proliferation, migration, and extracellular matrix synthesis [8]. The mechanisms of VSMC phenotypic modulation in arterial disease, including coronary artery disease (CAD), are not fully understood. Understanding VSMC differentiation in disease states might offer insights into VSMC phenotypic modulation and the pathogenesis of CAD.

In the past decade, several studies have reported the regulatory role of microRNAs (miRNAs) in VSMC phenotypic modulation [3,4]. These short (approximately 24 nucleotides), noncoding RNAs bind to the 3’ untranslated region of targeted mRNAs and induce degradation or translational inhibition [9]. Thus, the expression of miRNAs is inversely related to the expression of their targeted mRNAs. Muscleblind-like splicing regulator 1 (*MBNL1*), a novel target gene investigated in our study, was recently reported to promote muscle cell differentiation in mouse myoblasts [10]. No published study to date has investigated this mRNA in VSMCs in humans.

The dysregulation of miRNAs has been associated with VSMC phenotypic modulation and the progression of cardiovascular diseases, but the mechanisms are poorly understood [11]. In our study, we aimed to identify novel miRNA targets that could lead to a greater understanding of VSMC proliferation and differentiation in CAD.

## 2. Results

### 2.1. Demographic Characteristics of the Patients Recruited for This Study 

The clinical characteristics of the myocardial infarction (MI) (*n* = 16) and non-MI (*n* = 13) patients recruited for this study are presented in Table 1. There were no significant intergroup differences in demographic or clinical characteristics. Troponin I levels were significantly higher in the MI group than in the non-MI group.

### 2.2. Characteristics of miRNAs in VSMCs from the Atherosclerotic Wall

**Figure 1:**
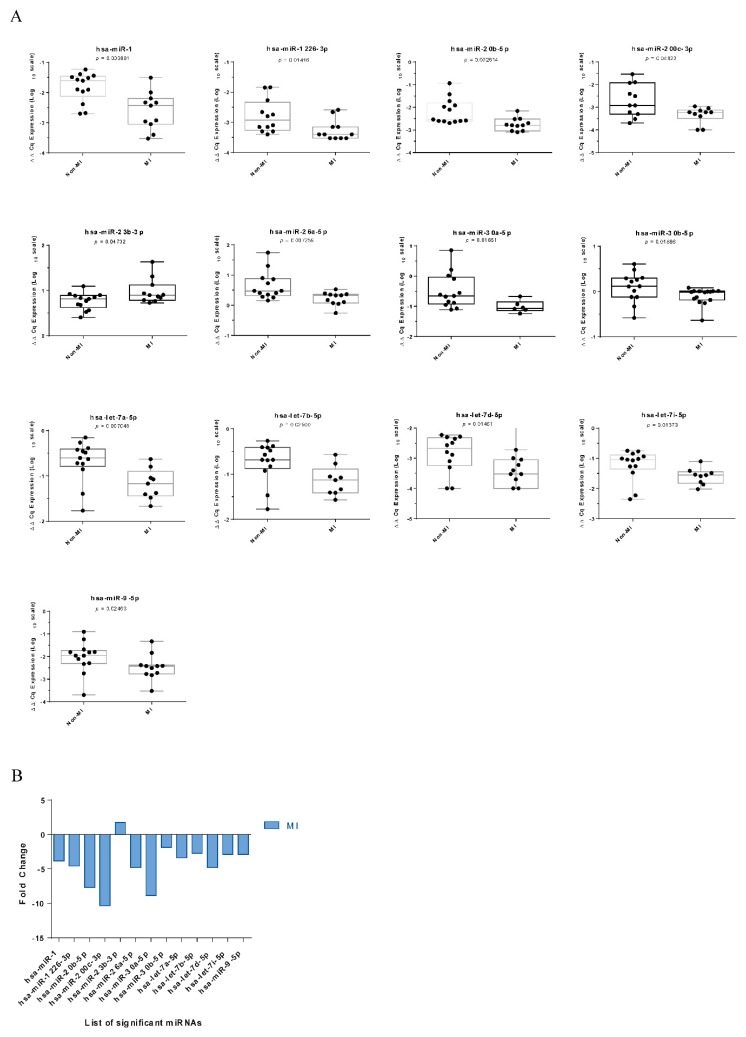
(**A**) Expression patterns of miRNAs significantly (*p* ≤ 0.05) downregulated in MI-related aortic samples. (**B**) The expressions of *hsa-miR-1, hsa-miR-1226-3p, hsa-miR-20b-5p, hsa-miR-200c-3p, hsa-miR-26a-5p, hsa-miR-30a-5p, hsa-miR-30b-5p, hsa-miR-let-7a-5p, hsa-miR-let-7b-5p, hsa-miR-let-7d-5p, hsa-miR-let-7i-5p*, and *hsa-miR-9-5p* were lower in MI samples than in non-MI samples, while *hsa-miR-23b-3p* was upregulated in the MI group.

Of 74 profiled miRNAs, 13 were found to be significantly differentially expressed (Figure 1). All differentially expressed miRNAs except *hsa-miR-23b-3p* were downregulated in the MI group compared to the non-MI group.

**Figure 2:**
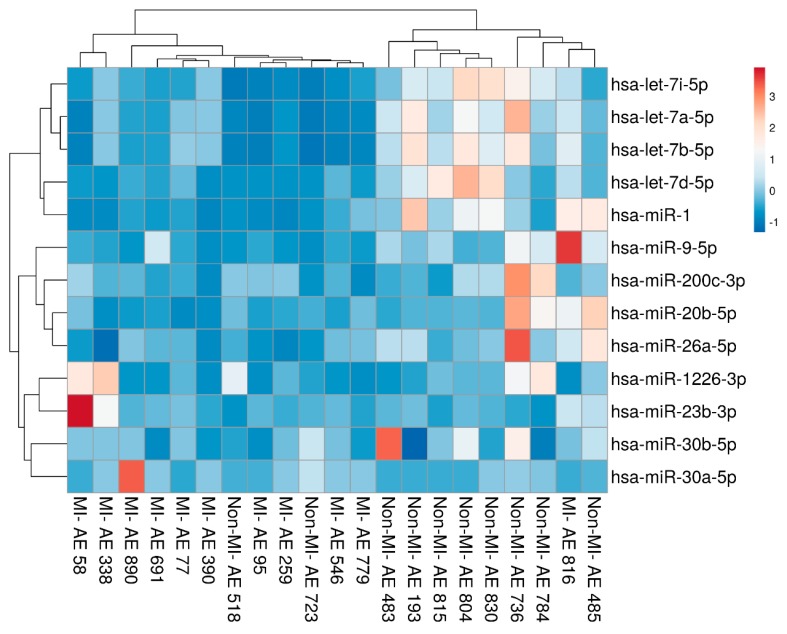
Hierarchical clustering analysis of the differentially expressed miRNAs found in vascular smooth muscle cells (VSMCs) from aortic wall tissues. Each row represents the miRNA expression in MI versus non-MI samples; the columns show the expression profiles of the subjects in the MI and non-MI groups. Upregulated miRNAs are indicated in red, while downregulated miRNAs are indicated in blue.

Hierarchical clustering analysis was conducted for the 13 differentially expressed miRNAs. Only samples with positive real-time quantitative polymerase chain reaction (RT-qPCR) data were selected for clustering analysis. Therefore, gene expression data from 11 MI and 10 non-MI samples were used. The clustering result is shown in Figure 2. The differential expression of the 13 miRNA targets distinguished MI from non-MI samples with 86% accuracy (sensitivity of 83% and specificity of 89%).

### 2.3. Target mRNA Prediction for Differentially Expressed miRNAs

Target mRNA prediction based on negatively regulated miRNAs was performed using Ingenuity Pathway Analysis (IPA) software (Ingenuity® Systems, www.qiagen.com/ingenuity), the DNA Intelligent Analysis (DIANA)-MicroT-CDS V5.0 prediction algorithm (http://diana.imis.athena-innovation.gr/DianaTools/index.php) [12], and the miRNA Enrichment Analysis and Annotation (miEAA) Tool (https://ccb-compute2.cs.uni-saarland.de/mieaa_tool/) [13]. For this analysis, we used the 12 downregulated differentially expressed miRNAs. The predictive analysis generated 3648 common potential predicted target mRNAs (Supplementary Table S1). The list of 3648 potential target mRNA was cross-matched with our own data concerning upregulated genes derived from a previously published microarray study performed on the same group of patients [5]. Of the 3648 predicted miRNA-targeted genes, 276 genes (Supplementary Table S2) were upregulated and inversely correlated with the associated targeting miRNA(s) among the 12 miRNAs that were significantly downregulated.

### 2.4. Biological Function Analysis of Predicted miRNA Target Genes

Gene ontology (GO) analysis of the 276 predicted miRNA-targeted genes was performed with Cytoscape V3.6.0 (http://www.cytoscape.org/) with the ClueGO V2.5 plugin [14]. The top 10 significantly enriched biological processes (adjusted *p*-value < 0.05) were related to muscle tissue: muscle tissue development, striated muscle tissue development, type I pneumocyte differentiation, muscle structure development, skeletal muscle organ development, heart development, regulation of muscle tissue development, regulation of striated muscle tissue development, regulation of muscle organ development, and regulation of skeletal muscle cell differentiation. The GO biological processes and associated mRNA are listed in Table 2.

To improve our understanding of VSMC-related processes, we narrowed down the identified 276 predicted mRNA target genes to those influenced by VSMC-related miRNAs. The list of 276 predicted miRNA target genes was cross-matched with a published list of 21-mRNA classifiers associated with VSMCs [5]. The list of matched genes is shown in Table 3.

**Figure 3:**
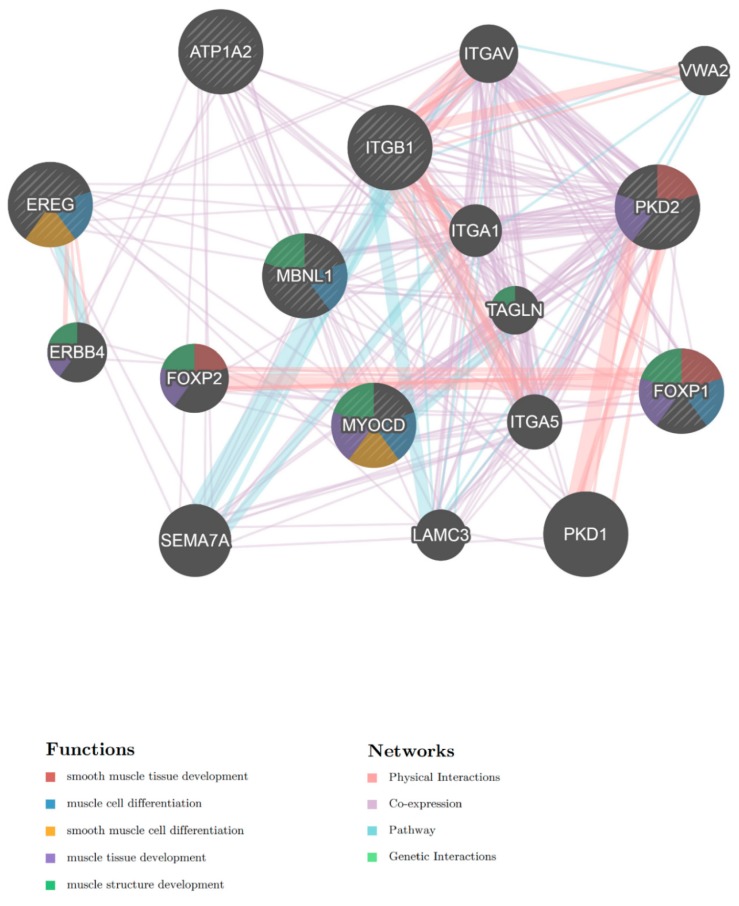
Network analysis of predicted miRNA target genes shows associations with muscle cell differentiation.

Network analysis of the seven selected genes was visualized using the GeneMANIA [15] plugin for Cytoscape [16] software (Figure 3). In the network analysis, *EREG*, *FOXP1*, *MBNL1*, and *MYOCD* were related to muscle cell differentiation, *FOXP1* and *PKD2* were related to SM tissue development, and *EREG* and *MYOCD* were related to smooth muscle cell (SMC) differentiation.

### 2.5. Analysis of Predicted miRNA Target mRNAs Associated with Muscle Cell Differentiation

Network analysis revealed four differentially expressed genes (*EREG*, *FOXP1*, *MBNL1*, and *MYOCD*) in VSMCs that were related to muscle cell differentiation. The analysis also brought our attention to a novel gene, *MBNL1*, which has not yet been reported to regulate VSMC differentiation in humans. Hence, *MBNL1* was selected for a more comprehensive analysis to understand its potential role in VSMC differentiation. Based on IPA software analysis, *MBNL1* was a predicted target gene for *hsa-miR-30b-5p*, in accordance with our finding that *hsa-miR-30b-5p* and *MBNL1* mRNA were inversely correlated in VSMCs.

**Figure 4:**
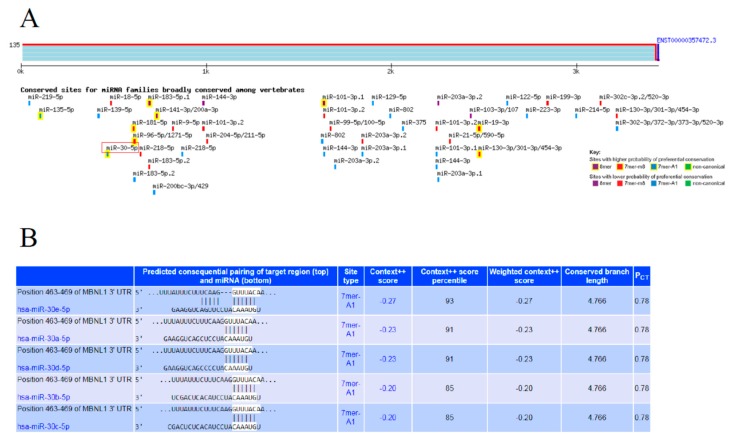
(**A**) Prediction results from TargetScan 7.1 revealed the potential site recognized by the seed sequences of *hsa-miR-30b-5p* in *MBNL1*; (**B**) *hsa-miR-30b-5p* had a context++ score of 85, suggesting that it is the most representative miRNA that regulates the expression of *MBNL1*.

An independent bioinformatics tool, TargetScan 7.1 (http://www.targetscan.org/vert_71/) [17], was used to predict miRNA targets and found that *MBNL1* contained potential sites for *hsa-miR-30b-5p*. The results of this analysis (Figure 4) showed that *MBNL1* has potential recognition sites for 7-mer-A1 seed sequences of *hsa-miR-30b-5p* with a context++ score of 85, suggesting that *hsa-miR-30b-5p* is the most representative miRNA that strongly regulates the expression of *MBNL1*.

### 2.6. Experimental Evaluation of Regulatory Function of hsa-miR-30b-5p on MBNL1 in Human VSMC

**Figure 5:**
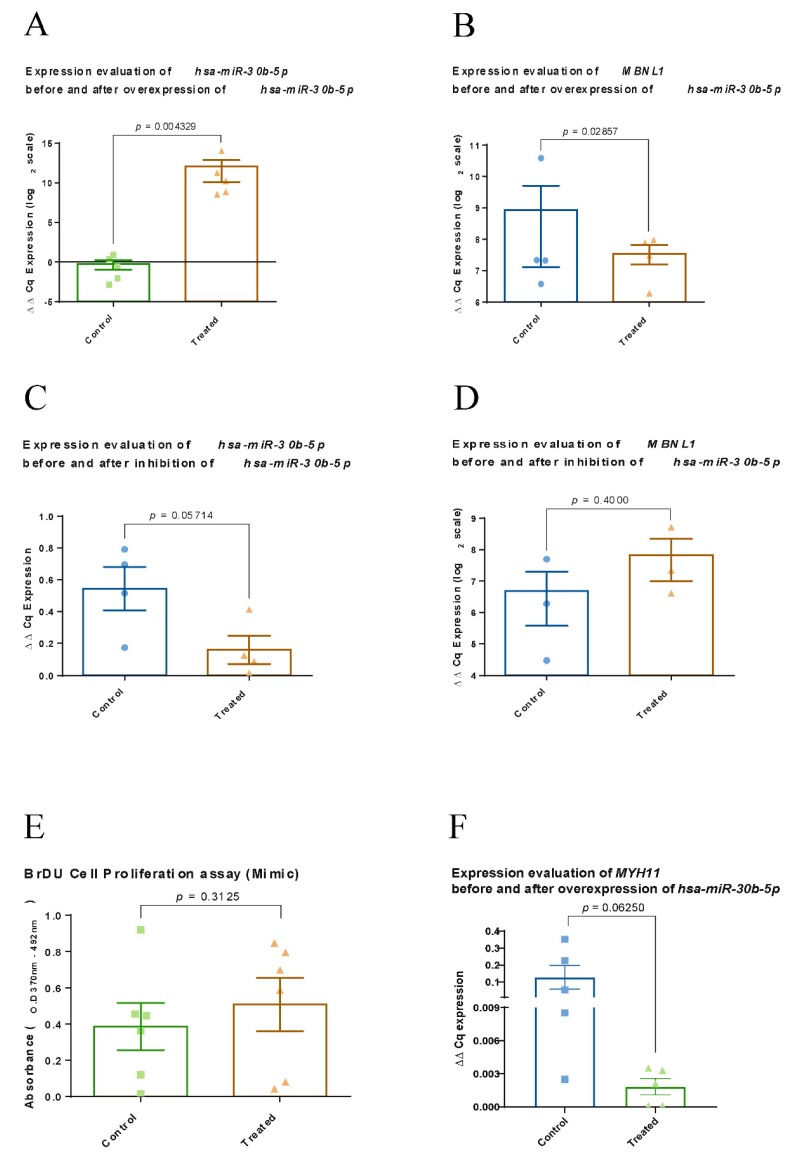
Evaluation of *hsa-miR-30b-5p* and *MBNL1* after 24 h of overexpression and inhibition experiments. (**A,C**) The expressions of *hsa-miR-30b-5p* after 24 h of overexpression and inhibition, respectively. (**B,D**) Corresponding expressions of *MBNL1*. (**E**) The proliferative index change after overexpression of *hsa-miR-30b-5p*. (**F**) Expressions of *MYH11* after overexpression of *hsa-miR-30b-5p.*

To confirm the predicted regulatory role of *hsa-miR-30b-5p* on *MBNL1*, we used commercially available human VSMC culture. The *hsa-miR-30b-5p* gain-of-function (mimic) and loss-of-function (inhibition) study was conducted using a transfection protocol (with at least three biological and three technical replicates). The levels of *hsa-miR-30b-5p* and *MBNL1* were quantitated by RT-qPCR. Statistical analysis was performed using non-parametric Mann–Whitney U test. The RNA expression results are displayed as relative expression values (Figure 5). In the *hsa-miR-30b-5p* mimic experiment, we were able to reach significant overexpression of *hsa-miR-30b-5p* with *p* = 0.043 *(*Figure 5A). After 24 h of overexpressed *hsa-miR-30b-5p* in the same VSMC culture, we evaluated *MBNL1* expression level and found it to be significantly downregulated with *p* = 0.028 (Figure 5B). In the loss of *hsa-miR-30b-5p* function experiment, we inhibited the level of *hsa-miR-30b-5p* (Figure 5C). Corresponding assessment of *MBNL1* in the same VSMC culture revealed upregulation of *MBNL1* expression; however, the trend did not reach significance level. Assessed *MYH11,* as common marker of VSMC differentiation, revealed a non-significant drop after 24 h of overexpressed *hsa-miR-30b-5p* (Figure 5F). 

The VSMC culture with significant upregulation of *MBNL1* in the mimic experiment was assessed further using a bromodeoxyuridine (BrdU) cell proliferation assay. The BrdU cell proliferation index is shown in Figure 5E. Our data showed an overexpression of *hsa-miR-30b-5p* uptrend proliferation index in human VSMC culture (non-significant change). 

## 3. Discussion

The VSMC phenotype is influenced by signals from the local environment, including miRNAs. To date, several studies have demonstrated that miRNAs are pleiotropic modulators of VSMC differentiation [18,19,20,21]. The molecular mechanisms underlying the regulation of VSMC differentiation by these miRNAs are diverse, but mainly involve the direct or indirect regulation of target gene expression. Alterations in VSMC differentiation promote plaque formation, which is characteristic of the progression of atherosclerosis [22,23,24].

In our study, miRNA profiling was performed on the genetic material isolated from VSMCs from the aortic wall. Thirteen miRNAs were significantly differentially expressed in VSMCs from MI versus non-MI aortic samples. Co-analysis of differentially expressed mRNAs narrowed our search to four differentially expressed genes that could participate in the cascade of events leading to muscle cell differentiation: *EREG*, *FOXP1*, *MBNL1*, and *MYOCD*. Among the genes involved in muscle cell differentiation, *EREG*, *FOXP1*, and *MYOCD* have been previously reported in several studies on the muscle cell differentiation process in humans [25]. Previous publications have reported that *MBNL1* is involved in RNA splicing events and the regulation of mouse myoblast differentiation, but little is known about its involvement in VSMC differentiation, especially in humans [10,26].

The ability of VSMC to transition between contractile and synthetic phenotypes has been reported in several studies. Epidermal growth factor-like ligands (e.g., *EREG*), which are expressed by VSMC, potently activate ERK and p38 MAPK, resulting in VSMC dedifferentiation [27]. Myocardin (*MYOCD*), a critical factor for SMC differentiation, interacts with serum response factor, activates SMC reporter genes and triggers signaling pathways that induce SMC differentiation [28,29,30]. 

In addition to *EREG* and *MYOCD*, the expression of forkhead box protein subfamily P1 (*FOXP1*), a class of transcription factors, was also upregulated in our previous gene expression study [5]. *FOXP1* is known to be an important regulator of several processes in cardiovascular development, such as cardiac muscle cell differentiation and proliferation [31]. Muscleblind-like splicing regulator 1 (*MBNL1*), a novel target gene discovered in our study, has been reported to promote muscle cell differentiation in mouse myoblasts [10]. Based on our preliminary data from aortic wall VSMCs isolated using laser captured microdissection (LCM), we suggest that *MBNL1* could play an important role in human VSMC regulation. Based on the prediction results from TargetScan, we also found that *MBNL1* has a potential recognition site for *hsa-miR-30b-5p* seed sequences, suggesting that *hsa-miR-30b-5p* could regulate the expression of *MBNL1*. Although the roles of both *hsa-miR-30b-5p* and *MBNL1* in VSMC differentiation are not well defined, these findings suggested a plausible relationship between *hsa-miR-30b-5p* and *MBNL1* in VSMC differentiation. Based on these data, we hypothesize that the expression of *MBNL1* is downregulated by overexpressed *hsa-miR-30b-5p*.

Our experiments in human VSMC culture with the mimic and inhibition protocols were able to confirm that overexpression of *hsa-miR-30b-5p* indeed downregulated *MBNL1* expression level. To our knowledge, this is the first human VSMC culture experimental confirmation of *hsa-miR-30b-5p* regulatory role on *MBNL1* transcript. The cell proliferation experimental study revealed that overexpression of *hsa-miR-30b-5p* and corresponding *MBNL1* downregulation could affect the proliferation of human VSMC culture. Moreover, assessed mRNA *MYH11,* as common marker of VSMC differentiation, dropping after 24 h of overexpressed *hsa-miR-30b-5p.*

## 4. Materials and Methods 

### 4.1. Study Population

This study was approved by the National Healthcare Group Domain Specific Review Board, Singapore (NHG DSRB Reference Number: 2009/00216). Written consent was obtained from patients prior to the study. Patients with CAD who underwent coronary artery bypass grafting surgery at National University Hospital, Singapore were recruited. The study population comprised of a group that had experienced a MI and a group that had not (non-MI). The MI group included uncomplicated patients with a recent ST-elevation (MI less than 5 days before the operation) or non-ST-elevation MI (*n* = 16). The non-MI group comprised patients (*n* = 13) with stable angina according to the existing American College of Cardiology guidelines (with no history of any acute cardiovascular event within three months prior to surgery). Cardiac status was documented by coronary angiogram, electrocardiogram, and plasma troponin (hsTnI). Patient demographics and clinical characteristics are displayed in Table 1. All patients were receiving beta-blockers, statins, and anti-platelet therapies.

**Figure 6:**
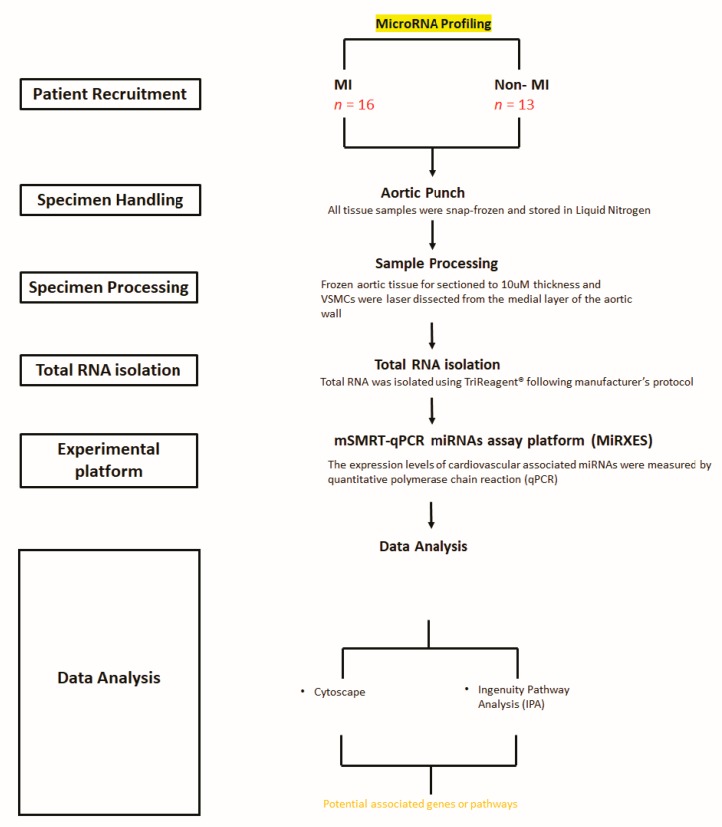
Workflow of tissue processing and data analysis for this miRNA- and mRNA-associated study.

Perioperative aortic punch samples from participating patients underwent LCM for the isolation of VSMCs. Total RNA was extracted from LCM tissue. The miRNA profile was evaluated using the mSMRT-qPCR miRNA assay platform (MiRXES, Singapore). miRNA profiling was performed using RT-qPCR. A schematic diagram of the project’s workflow is presented in Figure 6.

### 4.2. Aortic Wall Tissue Collection

Aortic wall tissues were collected at the time of proximal anastomosis between the aorta and saphenous vein grafts. These aortic wall samples were immediately cryopreserved on dry ice before being transferred to a liquid nitrogen tank for prolonged storage (NHG DSRB Tissue Bank Registration Number: NUH/2009-0073).

### 4.3. Cryosectioning, Staining, and LCM of VSMC

Frozen aortic wall tissues were embedded in Tissue-Tek® optimal cutting temperature compound (Sakura Finetek, Torrance, CA, USA), cryosectioned to a thickness of 10 µm, and placed on an RNase-free polyethylene naphthalate membrane slide (Carl Zeiss Microscopy, Jena, Germany). Each slide was stained with an Arcturus Histogene LCM Frozen Section Staining Kit (Applied Biosystems, Carlsbad, CA, USA). LCM was performed immediately upon completion of staining. PALM® MicroBeam (Carl Zeiss Microscopy, Jena, Germany) was used to dissect VSMCs from the aortic wall sections. The dissected VSMCs were then scraped into a microcentrifuge tube containing 100 µL of ice-cold TRI Reagent® (Molecular Research Center, Cincinnati, OH, USA) using 19 G sterile needles, and cryopreserved on dry ice until genomic processing.

### 4.4. RNA Processing and miRNA Profiling, Heatmapping, and Clustering

Total RNA was isolated from VSMC with TRI Reagent® (Molecular Research Center, Cincinnati, OH, USA) following the manufacturer’s protocol. Synthetic spike-in miRNAs (MiRXES, Singapore) were added to the isolated RNA samples, which served as internal controls.

Approximately 1 ng of total RNA per sample was reverse transcribed into cDNA prior to RT-qPCR with the IDEAL Individual miRNA qPCR assay kit (MiRXES, Singapore). RT-qPCR was performed using the mSMRT-qPCR miRNA assay platform (MiRXES, Singapore). Due to the limited availability of starting material, we narrowed our exploratory study to a total of 74 SMC-associated miRNAs that were related to VSMC phenotypic modulation according to a literature search in NCBI PubMed (http://www.ncbi.nlm.nih.gov/pubmed/) [11,32,33,34,35,36].

The raw data were normalized using reference miRNA by the 2^−∆∆CT^ method. The nonparametric Mann–Whitney U Test was applied to compare the profiling data of the two groups, i.e., MI and non-MI. In all analyses, a *p*-value < 0.05 was considered statistically significant. Hierarchical clustering of the MI and non-MI samples and heatmapping of the expression of the miRNA were performed with the R package (https://cran.r-project.org/) and Pvclust [37].

### 4.5. Transfection of miRNA Oligonucleotides

VSCM cell lines (human aortic smooth muscle cells, #CC-2916, Lonza, Basel, Switzerland) were propagated with SmGM™-2 Smooth Muscle Cell Growth Medium -2 BulletKit™ (#CC-3182, Lonza, Basel, Switzerland) as per the manufacturer’s instructions at 37 °C in a humidified atmosphere with 5% CO_2_.

The lyophilized oligonucleotides miRCURY LNA *hsa-miR-30b-5p* mimic, miRCURY LNA *hsa-miR-30b-5p* Power Inhibitor, and their relevant control miRNAs (mimic control and inhibitor control) (QIAGEN, Maryland, USA) were transfected by using the Lipofectamine RNAiMAX transfection reagent (Life Technologies, Carlsbad, CA, USA) according to the manufacturer’s instructions at a final concentration of 50 nM for 24 h. The RNA expressions were detected by RT-qPCR.

The Cell Proliferation ELISA, BrdU (colorimetric) kit (Roche Diagnostics, Mannheim, Germany) was used to measure cell proliferation according to the manufacturer’s protocol. VSMCs were seeded at 1000 cells per well into 96 well plates. The BrdU solution was added into the wells 24 h after transfection. Absorbance was measured at 370 nm (reference wavelength 492 nm).

### 4.6. Data Processing and Analysis

Hierarchical clustering analysis of differentially expressed miRNAs was conducted using ClustVis Software [38].

Significantly downregulated miRNAs were selected to investigate the relationship with SMC-associated genes from gene expression analysis [5]. Assessment of putative miRNA targets and SMC-associated genes was performed using the IPA software. The miRNA–mRNA networks between miRNA and SMC-associated genes were visualized using the GeneMANIA [15] plugin for Cytoscape [16] software.

GO was assessed using Cytoscape V3.6.0 with the ClueGO V2.5 plugin [14]. ClueGO allows determination of the distribution of the list of targeted mRNAs across GO terms. The adjusted *p*-value was calculated using right-sided hypergeometric tests, and the Benjamin–Hochberg adjustment was used for multiple test correction. An adjusted *p*-value < 0.05 indicates a statistically significant deviation from the expected distribution.

Identification of predicted miRNA target genes is critical for characterizing and understanding the functions of miRNAs. Computational bioinformatics tools, including the IPA software, the DIANA-MicroT-CDS V5.0 prediction algorithm, and the miEAA Tool, were used to predict the target genes of the differentially expressed miRNAs.

The GraphPad software (GraphPad Software, San Diego, CA, USA) was used for statistical analysis of gene expression BrdU cell proliferation assay results. Data are presented as median and were evaluated with Wilcoxon matched-pairs signed rank test.

## 5. Conclusions

Our predicted association analysis indicated that *miR-30b-5p* and *MBNL1* are negatively correlated in human VSMCs. The *hsa-miR-30b-5p* gain-of-function (mimic) study conducted via transfection experiments on human VSMC culture confirmed that overexpression of *hsa-miR-30b-5p* downregulates *MBNL1* expression level. To our knowledge, this is the first in human VSMC culture experimental confirmation of the regulatory role of *hsa-miR-30b-5p* on *MBNL1* transcript. However, additional experimental work should be conducted to confirm that an interaction between *miR-30b-5p* and *MBNL1* plays genuine role in VSMC differentiation and proliferation. 

## Figures and Tables

**Table 1:** Demographic characteristics of the myocardial infarction (MI) and non-MI groups selected for miRNA profiling.

Characteristics		MI (*n* = 16)	Non-MI (*n* = 13)	*p*-Value
Ethnicity	Chinese (%)	12 (75.0%)	7 (53.8%)	0.363
	Malay (%)	3 (18.8%)	3 (23.1%)	
	Others (%)	1 (6.3%)	3 (23.1%)	
Gender	Male (%)	14 (87.5%)	13 (100%)	0.186
	Female (%)	2 (12.5%)	0 (0%)	
Age (Mean ± SD)		62.19 ± 9.614	55.85 ± 6.401	0.052
Diabetes Mellitus	No (%)	6 (37.5%)	5 (38.5%)	0.958
	Yes (%)	10 (62.5%)	8 (61.5%)	
Hypertension	No (%)	1 (6.3%)	1 (7.7%)	0.879
	Yes (%)	15 (93.8%)	12 (92.3%)	
Hyperlipidemia	No (%)	0 (0%)	1 (7.7%)	0.259
	Yes (%)	16 (100%)	12 (92.3%)	
Smoking	No (%)	7 (43.8%)	6 (46.2%)	0.897
	Yes (%)	9 (56.2%)	7 (53.8%)	
Ejection Fraction	Good (>45%)	8 (50.0%)	9 (69.2%)	0.579
	Fair (30–45%)	6 (37.5%)	3 (23.1%)	
	Poor (<30%)	2 (12.5%)	1 (7.7%)	
Troponin I (µg/L) (Mean ± SD)		13.73 ± 4.768	3.377 ± 1.834	0.003

**Table 2:** The top 10 significantly enriched biological processes upregulated in MI patients (from gene ontology (GO) analysis of the 276 predicted miRNA target genes). The GO list revealed relationships with muscle tissue development, striated muscle tissue development, type I pneumocyte differentiation, muscle structure development, skeletal muscle organ development, heart development, regulation of muscle tissue development, regulation of striated muscle tissue development, regulation of muscle organ development, and regulation of skeletal muscle cell differentiation.

GOID	GO Term	Term *p-*value	Term *p-*value Corrected with Benjamini-Hochberg	% Associated	Associated mRNA
GO:0060537	muscle tissue development	0.000000	0.000021	5.56	*ACADM, AKAP6, ARID2, COL11A1, CREB1, DDX5, FOXP1, HNRNPU, HOMER1, IGF1, ITGB1, LEMD2, MEF2D, MYOCD, NLN, NR1D2, PDGFRA, PKD2, PRKAA1, RBFOX1, RPS6KB1, SKIL]*
GO:0060537	muscle tissue development	0.000000	0.000021	5.56	*[ACADM, AKAP6, ARID2, COL11A1, CREB1, DDX5, FOXP1, HNRNPU, HOMER1, IGF1, ITGB1, LEMD2, MEF2D, MYOCD, NLN, NR1D2, PDGFRA, PKD2, PRKAA1, RBFOX1, RPS6KB1, SKIL]*
GO:0014706	striated muscle tissue development	0.000000	0.000022	5.56	*[ACADM, AKAP6, ARID2, COL11A1, CREB1, DDX5, FOXP1, HNRNPU, HOMER1, IGF1, ITGB1, LEMD2, MEF2D, MYOCD, NLN, NR1D2, PDGFRA, PRKAA1, RBFOX1, RPS6KB1, SKIL]*
GO:0014706	striated muscle tissue development	0.000000	0.000022	5.56	*[ACADM, AKAP6, ARID2, COL11A1, CREB1, DDX5, FOXP1, HNRNPU, HOMER1, IGF1, ITGB1, LEMD2, MEF2D, MYOCD, NLN, NR1D2, PDGFRA, PRKAA1, RBFOX1, RPS6KB1, SKIL*
GO:0060509	type I pneumocyte differentiation	0.000000	0.000025	80.00	*CREB1, NFIB, THRA, THRB*
GO:0061061	muscle structure development	0.000002	0.000156	4.05	*ACADM, AKAP6, ANKRD17, BASP1, COL11A1, CREB1, DDX5, EREG, ETV1, FOXP1, HNRNPU, HOMER1, IGF1, ITGB1, LEMD2, MBNL1, MEF2D, MYOCD, NLN, NR1D2, PDGFRA, PRKAA1, RBFOX1, RPS6KB1, SKIL, THRA*
GO:0061061	muscle structure development	0.000002	0.000156	4.05	*ACADM, AKAP6, ANKRD17, BASP1, COL11A1, CREB1, DDX5, EREG, ETV1, FOXP1, HNRNPU, HOMER1, IGF1, ITGB1, LEMD2, MBNL1, MEF2D, MYOCD, NLN, NR1D2, PDGFRA, PRKAA1, RBFOX1, RPS6KB1, SKIL, THRA*
GO:0060538	skeletal muscle organ development	0.000002	0.000158	7.14	*BASP1, DDX5, FOXP1, HOMER1, LEMD2, MEF2D, MYOCD, NLN, NR1D2, PRKAA1, RBFOX1, RPS6KB1, SKIL*
GO:0016202	regulation of striated muscle tissue development	0.000003	0.000170	8.40	*AKAP6, CREB1, DDX5, FOXP1, IGF1, MYOCD, NLN, NR1D2, PRKAA1, RBFOX1, RPS6KB1*
GO:1901861	regulation of muscle tissue development	0.000003	0.000169	8.27	*AKAP6, CREB1, DDX5, FOXP1, IGF1, MYOCD, NLN, NR1D2, PRKAA1, RBFOX1, RPS6KB1*

**Table 3:** miRNA-targeted VSMC-related genes (based on novel 21-gene classifiers). The seven genes were negatively regulated in the group of MI patients.

miRNA	Target Gene Symbol	Description	Biological Processes
*hsa-miR-1,* *hsa-let-7b-5p*	*FOXP1*	forkhead box P1	heart development
*hsa-miR-1*	*MYOCD*	myocardin	regulation of smooth muscle contraction
*hsa-miR-20b-5p*	*PKD2*	polycystic kidney disease 2	heart development
*hsa-miR-30b-5p*	*MBNL1*	muscleblind-like	striated muscle tissue development
*hsa-miR-20b-5p*	*ATP1A2*	ATPase, Na+/K+ transporting, alpha 2 (+) polypeptide	regulation of smooth muscle contraction
*hsa-miR-20b-5p,* *hsa-miR-26a-5p*	*EREG*	epiregulin	regulation of muscle cell differentiation
*hsa-miR-1226-3p*	*ITGB1*	integrin, beta 1	heart development

## Supplementary Materials

Supplementary materials can be found at https://www.mdpi.com/1422-0067/21/1/11/s1.

## Abbreviations

VSMC	Vascular smooth muscle cells
miRNAs	microRNAs
SM	Smooth muscle
CAD	Coronary artery disease
MI	Myocardial infarction
RT-qPCR	Real-time polymerase chain reaction
IPA	Ingenuity Pathway Analysis
DIANA	DNA Intelligent Analysis
miEAA	miRNA Enrichment Analysis and Annotation
GO	Gene ontology
SMC	Smooth muscle cell
BrdU	Bromodeoxyuridine
*MYOCD*	Myocardin
*MBNL1*	Muscleblind-like splicing regulator 1
LCM	Laser capture microdissection
