# Supplementary material for: The Interaction between 30b-5p miRNA and MBNL1 mRNA is Involved in Vascular Smooth Muscle Cell Differentiation in Patients with Coronary Atherosclerosis
Source: Int J Mol Sci. 2019 Dec 18;21(1):11. doi: 10.3390/ijms21010011 (PMC6982107; doi:10.3390/ijms21010011)
Supplement: Supplementary file 1 [file ijms-21-00011-s001.zip › Supplementary Table S1.pdf]

Supplemetary Table 1: Predictive analysis generated 3,648 common potential predicted target mRNAs, which were further cross-matched with our transcriptomic profiling data to identify potential associating miRNA-mRNA targets.

Gene Symbol	Description
A1CF	APOBEC1 Complementation Factor
AAK1	AP2 Associated Kinase 1
AAMP	Angio Associated Migratory Cell Protein
AARD	Alanine And Arginine Rich Domain Containing Protein
AASDHPPT	Aminoadipate-Semialdehyde Dehydrogenase-Phosphopantetheinyl Transferase
AATK	Apoptosis Associated Tyrosine Kinase
ABCA12	ATP Binding Cassette Subfamily A Member 12
ABCB7	ATP Binding Cassette Subfamily B Member 7
ABCB9	ATP Binding Cassette Subfamily B Member 9
ABCC10	ATP Binding Cassette Subfamily C Member 10
ABCC5	ATP Binding Cassette Subfamily C Member 5
ABCC8	ATP Binding Cassette Subfamily C Member 8
ABCC9	ATP Binding Cassette Subfamily C Member 9
ABCD4	ATP Binding Cassette Subfamily D Member 4
ABCE1	ATP Binding Cassette Subfamily E Member 1
ABCG1	ATP Binding Cassette Subfamily G Member 1
ABCG2	ATP Binding Cassette Subfamily G Member 2 (Junior Blood Group)
ABHD13	Abhydrolase Domain Containing 13
ABHD14B	Abhydrolase Domain Containing 14B
ABI2	Abl Interactor 2
ABI3BP	ABI Family Member 3 Binding Protein
ABL1	ABL Proto-Oncogene 1, Non-Receptor Tyrosine Kinase
ABL2	ABL Proto-Oncogene 2, Non-Receptor Tyrosine Kinase
ABR	ABR, RhoGEF And GTPase Activating Protein
ABRA	Actin Binding Rho Activating Protein
ABT1	Activator Of Basal Transcription 1
ACADM	Acyl-CoA Dehydrogenase Medium Chain

ACADSB	Acyl-CoA Dehydrogenase Short/Branched Chain
ACADVL	Acyl-CoA Dehydrogenase Very Long Chain
ACAP2	ArfGAP With Coiled-Coil, Ankyrin Repeat And PH Domains 2
ACBD5	Acyl-CoA Binding Domain Containing 5
ACE2	Angiotensin I Converting Enzyme 2
ACER2	Alkaline Ceramidase 2
ACER3	Alkaline Ceramidase 3
ACKR4	Atypical Chemokine Receptor 4
ACOT11	Acyl-CoA Thioesterase 11
ACOT4	Acyl-CoA Thioesterase 4
ACP1	Acid Phosphatase 1
ACRV1	Acrosomal Vesicle Protein 1
ACSL3	Acyl-CoA Synthetase Long Chain Family Member 3
ACSL4	Acyl-CoA Synthetase Long Chain Family Member 4
ACSL6	Acyl-CoA Synthetase Long Chain Family Member 6
ACTA1	Actin, Alpha 1, Skeletal Muscle
ACTB	Actin Beta
ACTC1	Actin, Alpha, Cardiac Muscle 1
ACTG2	Actin, Gamma 2, Smooth Muscle, Enteric
ACTN1	Actinin Alpha 1
ACTR1A	ARP1 Actin Related Protein 1 Homolog A
ACTR3	ARP3 Actin Related Protein 3 Homolog
ACVR1	Activin A Receptor Type 1
ACVR1B	Activin A Receptor Type 1B
ACVR1C	Activin A Receptor Type 1C
ACVR2A	Activin A Receptor Type 2A
ACVR2B	Activin A Receptor Type 2B
ADA	Adenosine Deaminase
ADAM10	ADAM Metallopeptidase Domain 10
ADAM12	ADAM Metallopeptidase Domain 12
ADAM15	ADAM Metallopeptidase Domain 15
ADAM17	ADAM Metallopeptidase Domain 17

ADAM18	ADAM Metallopeptidase Domain 18
ADAM22	ADAM Metallopeptidase Domain 22
ADAM28	ADAM Metallopeptidase Domain 28
ADAMTS1	ADAM Metallopeptidase With Thrombospondin Type 1 Motif 1
ADAMTS12	ADAM Metallopeptidase With Thrombospondin Type 1 Motif 12
ADAMTS14	ADAM Metallopeptidase With Thrombospondin Type 1 Motif 14
ADAMTS15	ADAM Metallopeptidase With Thrombospondin Type 1 Motif 15
ADAMTS3	ADAM Metallopeptidase With Thrombospondin Type 1 Motif 3
ADAMTS4	ADAM Metallopeptidase With Thrombospondin Type 1 Motif 4
ADAMTS5	ADAM Metallopeptidase With Thrombospondin Type 1 Motif 5
ADAMTS6	ADAM Metallopeptidase With Thrombospondin Type 1 Motif 6
ADAMTS8	ADAM Metallopeptidase With Thrombospondin Type 1 Motif 8
ADAMTS9	ADAM Metallopeptidase With Thrombospondin Type 1 Motif 9
ADAP2	ArfGAP With Dual PH Domains 2
ADCY1	Adenylate Cyclase 1
ADCY9	Adenylate Cyclase 9
ADD3	Adducin 3
ADHFE1	Alcohol Dehydrogenase, Iron Containing 1
ADIPOR2	Alcohol Dehydrogenase, Iron Containing 1
ADM	Adrenomedullin
ADNP	Activity Dependent Neuroprotector Homeobox
ADPGK	ADP Dependent Glucokinase
ADPRH	ADP-Ribosylarginine Hydrolase
ADRA1D	Adrenoceptor Alpha 1D
ADRA2A	Adrenoceptor Alpha 2A
ADRB1	Adrenoceptor Beta 1
ADRB2	Adrenoceptor Beta 2
ADRB3	Adrenoceptor Beta 3
AEBP2	AE Binding Protein 2
AEN	Apoptosis Enhancing Nuclease
AFAP1L2	Actin Filament Associated Protein 1 Like 2
AFF2	AF4/FMR2 Family Member 2

AFF3	AF4/FMR2 Family Member 3
AGBL2	ATP/GTP Binding Protein Like 2
AGFG1	ArfGAP With FG Repeats 1
AGFG2	ArfGAP With FG Repeats 2
AGO1	Argonaute 1, RISC Catalytic Component
AGO2	Argonaute 2, RISC Catalytic Component
AGO3	Argonaute 3, RISC Catalytic Component
AGO4	Argonaute 4, RISC Catalytic Component
AGPAT5	1-Acylglycerol-3-Phosphate O-Acyltransferase 5
AGTPBP1	ATP/GTP Binding Protein 1
AHCTF1	AT-Hook Containing Transcription Factor 1
AHNAK	AHNAK Nucleoprotein
AHR	Aryl Hydrocarbon Receptor
AHRR	Aryl-Hydrocarbon Receptor Repressor
AIDA	Axin Interactor, Dorsalization Associated
AIFM1	Apoptosis Inducing Factor Mitochondria Associated 1
AK2	Adenylate Kinase 2
AKAP10	A-Kinase Anchoring Protein 10
AKAP11	A-Kinase Anchoring Protein 11
AKAP5	A-Kinase Anchoring Protein 5
AKAP6	A-Kinase Anchoring Protein 6
AKNA	AT-Hook Transcription Factor
AKR1B10	Aldo-Keto Reductase Family 1 Member B10
AKR1C4	Aldo-Keto Reductase Family 1 Member C4
AKT3	AKT Serine/Threonine Kinase 3
AKTIP	AKT Interacting Protein
ALDH5A1	Aldehyde Dehydrogenase 5 Family Member A1
ALG10B	ALG10B, Alpha-1,2-Glucosyltransferase
ALG2	ALG2, Alpha-1,3/1,6-Mannosyltransferase
ALG8	ALG8, Alpha-1,3-Glucosyltransferase
ALG9	ALG9, Alpha-1,2-Mannosyltransferase
ALKBH1	AlkB Homolog 1, Histone H2A Dioxygenase

ALPK1	Alpha Kinase 1
ALPK3	Alpha Kinase 3
ALPP	Alkaline Phosphatase, Placental
AMACR	Alpha-Methylacyl-CoA Racemase
AMER2	APC Membrane Recruitment Protein 2
AMER3	APC Membrane Recruitment Protein 3
AMFR	autocrine motility factor receptor
AMMECR1	Alport syndrome, mental retardation, midface hypoplasia and elliptocytosis chromosomal region gene 1
AMMECR1L	AMMECR1 like
AMOT	angiomotin
AMOTL2	angiomotin like 2
ANKFY1	ankyrin repeat and FYVE domain containing 1
ANKIB1	ankyrin repeat and IBR domain containing 1
ANKRA2	ankyrin repeat family A member 2
ANKRD12	ankyrin repeat domain 12
ANKRD13C	ankyrin repeat domain 13C
ANKRD17	ankyrin repeat domain 17
ANKRD20A4	ankyrin repeat domain 20 family member A4
ANKRD23	ankyrin repeat domain 23
ANKRD28	ankyrin repeat domain 28
ANKRD29	ankyrin repeat domain 29
ANKRD34B	ankyrin repeat domain 34B
ANKRD39	ankyrin repeat domain 39
ANKRD49	ankyrin repeat domain 49
ANKRD52	ankyrin repeat domain 52
ANKRD6	ankyrin repeat domain 6
ANKS1B	ankyrin repeat and sterile alpha motif domain containing 1B
ANKS4B	ankyrin repeat and sterile alpha motif domain containing 4B
ANLN	anillin actin binding protein
ANO4	anoctamin 4
ANO6	anoctamin 6
ANP32B	acidic nuclear phosphoprotein 32 family member B

ANTXR1	ANTXR cell adhesion molecule 1
ANXA13	annexin A13
ANXA2	annexin A2
ANXA4	annexin A4
AP1B1	adaptor related protein complex 1 subunit beta 1
AP1G1	adaptor related protein complex 1 subunit gamma 1
AP1S1	adaptor related protein complex 1 subunit sigma 1
AP1S2	adaptor related protein complex 1 subunit sigma 2
AP2A1	adaptor related protein complex 2 subunit alpha 1
AP3D1	adaptor related protein complex 3 subunit delta 1
AP3M1	adaptor related protein complex 3 subunit mu 1
AP3S1	adaptor related protein complex 3 subunit sigma 1
AP3S2	adaptor related protein complex 3 subunit sigma 2
AP4E1	adaptor related protein complex 4 subunit epsilon 1
APAF1	apoptotic peptidase activating factor 1
APBA1	amyloid beta precursor protein binding family A member 1
APC	APC, WNT signaling pathway regulator
APCDD1	APC down-regulated 1
API5	apoptosis inhibitor 5
APOL6	apolipoprotein L6
APOOL	apolipoprotein O like
APPL1	adaptor protein, phosphotyrosine interacting with PH domain and leucine zipper 1
AQP5	aquaporin 5
ARAF	A-Raf proto-oncogene, serine/threonine kinase
ARAP2	ArfGAP with RhoGAP domain, ankyrin repeat and PH domain 2
ARCN1	archain 1
ARF3	ADP ribosylation factor 3
ARF4	ADP ribosylation factor 4
ARFIP1	ADP ribosylation factor interacting protein 1
ARG2	arginase 2
ARGLU1	arginine and glutamate rich 1
ARHGAP12	Rho GTPase activating protein 12

ARHGAP20	Rho GTPase activating protein 20
ARHGAP21	Rho GTPase activating protein 21
ARHGAP26	Rho GTPase activating protein 26
ARHGAP28	Rho GTPase activating protein 28
ARHGAP29	Rho GTPase activating protein 29
ARHGAP32	Rho GTPase activating protein 32
ARHGAP6	Rho GTPase activating protein 6
ARHGAP8	Rho GTPase activating protein 8
ARHGEF10	Rho guanine nucleotide exchange factor 10
ARHGEF15	Rho guanine nucleotide exchange factor 15
ARHGEF18	Rho/Rac guanine nucleotide exchange factor 18
ARHGEF28	Rho guanine nucleotide exchange factor 28
ARHGEF38	Rho guanine nucleotide exchange factor 38
ARHGEF40	Rho guanine nucleotide exchange factor 40
ARID1A	AT-rich interaction domain 1A
ARID2	AT-rich interaction domain 2
ARID3A	AT-rich interaction domain 3A
ARID3B	AT-rich interaction domain 3B
ARID4A	AT-rich interaction domain 4A
ARID4B	AT-rich interaction domain 4B
ARID5B	AT-rich interaction domain 5B
ARIH1	ariadne RBR E3 ubiquitin protein ligase 1
ARL10	ADP ribosylation factor like GTPase 10
ARL15	ADP ribosylation factor like GTPase 15
ARL2BP	ADP ribosylation factor like GTPase 2 binding protein
ARL3	ADP ribosylation factor like GTPase 3
ARL4A	ADP ribosylation factor like GTPase 4A
ARL4C	ADP ribosylation factor like GTPase 4C
ARL4D	ADP ribosylation factor like GTPase 4D
ARL5A	ADP ribosylation factor like GTPase 5A
ARL5B	ADP ribosylation factor like GTPase 5B
ARL6IP6	ADP ribosylation factor like GTPase 6 interacting protein 6

ARMC8	armadillo repeat containing 8
ARMCX2	armadillo repeat containing X-linked 2
ARMS2	age-related maculopathy susceptibility 2
ARPC3	actin related protein 2/3 complex subunit 3
ARPC5	actin related protein 2/3 complex subunit 5
ARPC5L	actin related protein 2/3 complex subunit 5 like
ARPP19	cAMP regulated phosphoprotein 19
ARPP21	cAMP regulated phosphoprotein 21
ARRDC4	arrestin domain containing 4
ARSB	arylsulfatase B
ART3	ADP-ribosyltransferase 3
ASAH2	N-acylsphingosine amidohydrolase 2
ASAH2B	N-acylsphingosine amidohydrolase 2B
ASAP1	ArfGAP with SH3 domain, ankyrin repeat and PH domain 1
ASAP2	ArfGAP with SH3 domain, ankyrin repeat and PH domain 2
ASB2	ankyrin repeat and SOCS box containing 2
ASF1A	anti-silencing function 1A histone chaperone
ASH1L	ASH1 like histone lysine methyltransferase
ASIC1	acid sensing ion channel subunit 1
ASPH	aspartate beta-hydroxylase
ASPN	asporin
ASXL2	ASXL transcriptional regulator 2
ATAD2	ATPase family, AAA domain containing 2
ATAD2B	ATPase family, AAA domain containing 2B
ATE1	arginyltransferase 1
ATF1	activating transcription factor 1
ATF2	activating transcription factor 2
ATG13	autophagy related 13
ATG16L1	autophagy related 16 like 1
ATG2B	autophagy related 2B
ATG5	autophagy related 5
ATG9A	autophagy related 9A

ATL2	atlastin GTPase 2
ATM	ATM serine/threonine kinase
ATP10B	ATPase phospholipid transporting 10B (putative)
ATP10D	ATPase phospholipid transporting 10D (putative)
ATP11B	ATPase phospholipid transporting 11B (putative)
ATP11C	ATPase phospholipid transporting 11C
ATP12A	ATPase H+/K+ transporting non-gastric alpha2 subunit
ATP13A3	ATPase 13A3
ATP1A2	ATPase Na+/K+ transporting subunit alpha 2
ATP2A2	ATPase sarcoplasmic/endoplasmic reticulum Ca2+ transporting 2
ATP2B1	ATPase plasma membrane Ca2+ transporting 1
ATP2B2	ATPase plasma membrane Ca2+ transporting 2
ATP2B3	ATPase plasma membrane Ca2+ transporting 3
ATP2C1	ATPase secretory pathway Ca2+ transporting 1
ATP6V0A2	ATPase H+ transporting V0 subunit a2
ATP6V1A	ATPase H+ transporting V1 subunit A
ATP6V1B2	ATPase H+ transporting V1 subunit B2
ATP6V1C1	ATPase H+ transporting V1 subunit C1
ATP6V1G1	ATPase H+ transporting V1 subunit G1
ATP8A1	ATPase phospholipid transporting 8A1
ATP8A2	ATPase phospholipid transporting 8A2
ATP8B4	ATPase phospholipid transporting 8B4 (putative)
ATP9A	ATPase phospholipid transporting 9A (putative)
ATPAF1	ATP synthase mitochondrial F1 complex assembly factor 1
ATRNL1	attractin like 1
ATRX	ATRX, chromatin remodeler
ATXN1	ataxin 1
ATXN1L	ataxin 1 like
ATXN7	ataxin 7
ATXN7L1	ataxin 7 like 1
ATXN7L2	ataxin 7 like 2
ATXN7L3	ataxin 7 like 3

ATXN7L3B	ataxin 7 like 3B
AVEN	apoptosis and caspase activation inhibitor
AXDND1	axonemal dynein light chain domain containing 1
AXL	AXL receptor tyrosine kinase
AZIN1	antizyme inhibitor 1
B3GALT5	beta-1,3-galactosyltransferase 5
B3GAT1	beta-1,3-glucuronyltransferase 1
B3GAT3	beta-1,3-glucuronyltransferase 3
B3GNT2	UDP-GlcNAc:betaGal beta-1,3-N-acetylglucosaminyltransferase 2
B3GNT5	UDP-GlcNAc:betaGal beta-1,3-N-acetylglucosaminyltransferase 5
B3GNT7	UDP-GlcNAc:betaGal beta-1,3-N-acetylglucosaminyltransferase 7
B4GALT1	beta-1,4-galactosyltransferase 1
B4GALT4	beta-1,4-galactosyltransferase 4
B4GALT6	beta-1,4-galactosyltransferase 6
BAAT	bile acid-CoA:amino acid N-acyltransferase
BACE2	beta-secretase 2
BACH1	BTB domain and CNC homolog 1
BACH2	BTB domain and CNC homolog 2
BAG4	BCL2 associated athanogene 4
BAG5	BCL2 associated athanogene 5
BAG6	BCL2 associated athanogene 6
BAHD1	bromo adjacent homology domain containing 1
BAK1	BCL2 antagonist/killer 1
BAP1	BRCA1 associated protein 1
BASP1	brain abundant membrane attached signal protein 1
BAZ2B	bromodomain adjacent to zinc finger domain 2B
BBIP1	BBSome interacting protein 1
BBX	BBX, HMG-box containing
BCAP29	B cell receptor associated protein 29
BCAT1	branched chain amino acid transaminase 1
BCCIP	BRCA2 and CDKN1A interacting protein
BCL10	B cell CLL/lymphoma 10

BCL11B	B cell CLL/lymphoma 11B
BCL2	BCL2, apoptosis regulator
BCL2L1	BCL2 like 1
BCL2L11	BCL2 like 11
BCL2L15	BCL2 like 15
BCL6	B cell CLL/lymphoma 6
BCL7A	BCL tumor suppressor 7A
BCL7B	BCL tumor suppressor 7B
BCO2	beta-carotene oxygenase 2
BCOR	BCL6 corepressor
BCR	BCR, RhoGEF and GTPase activating protein
BDNF	brain derived neurotrophic factor
BDP1	B double prime 1, subunit of RNA polymerase III transcription initiation factor IIIB
BECN1	beclin 1
BEGAIN	brain enriched guanylate kinase associated
BEND4	BEN domain containing 4
BEND7	BEN domain containing 7
BEST4	bestrophin 4
BET1	Bet1 golgi vesicular membrane trafficking protein
BET1L	Bet1 golgi vesicular membrane trafficking protein like
BHLHE22	basic helix-loop-helix family member e22
BHLHE40	basic helix-loop-helix family member e40
BHLHE41	basic helix-loop-helix family member e41
BICC1	BicC family RNA binding protein 1
BID	BH3 interacting domain death agonist
BIN3	bridging integrator 3
BLOC1S2	biogenesis of lysosomal organelles complex 1 subunit 2
BNC1	basonuclin 1
BNC2	basonuclin 2
BNIP2	BCL2 interacting protein 2
BOC	BOC cell adhesion associated, oncogene regulated
BOD1	biorientation of chromosomes in cell division 1

BOD1L2	biorientation of chromosomes in cell division 1 like 2
BOLL	boule homolog, RNA binding protein
BPY2	basic charge Y-linked 2
BRCA2	BRCA2, DNA repair associated
BRCC3	BRCA1/BRCA2-containing complex subunit 3
BRD3	bromodomain containing 3
BRF2	BRF2, RNA polymerase III transcription initiation factor subunit
BRI3BP	BRI3 binding protein
BRWD1	bromodomain and WD repeat domain containing 1
BRWD3	bromodomain and WD repeat domain containing 3
BSCL2	BSCL2, seipin lipid droplet biogenesis associated
BSDC1	BSD domain containing 1
BSN	bassoon presynaptic cytomatrix protein
BSND	barttin CLCNK type accessory beta subunit
BTAF1	B-TFIID TATA-box binding protein associated factor 1
BTBD10	BTB domain containing 10
BTBD3	BTB domain containing 3
BTBD7	BTB domain containing 7
BTBD9	BTB domain containing 9
BTF3L4	basic transcription factor 3 like 4
BTG2	BTG anti-proliferation factor 2
BTLA	B and T lymphocyte associated
BUB3	BUB3, mitotic checkpoint protein
BZW1	basic leucine zipper and W2 domains 1
BZW2	basic leucine zipper and W2 domains 2
C10orf55	chromosome 10 open reading frame 55
C11orf21	chromosome 11 open reading frame 21
C11orf87	chromosome 11 open reading frame 87
C12orf4	chromosome 12 open reading frame 4
C12orf49	chromosome 12 open reading frame 49
C14orf178	chromosome 14 open reading frame 178
C14orf28	chromosome 14 open reading frame 28

C14orf39	chromosome 14 open reading frame 39
C15orf39	chromosome 15 open reading frame 39
C15orf41	chromosome 15 open reading frame 41
C16orf72	chromosome 16 open reading frame 72
C16orf87	chromosome 16 open reading frame 87
C18orf21	chromosome 18 open reading frame 21
C18orf63	chromosome 18 open reading frame 63
C19orf12	chromosome 19 open reading frame 12
C1GALT1	core 1 synthase, glycoprotein-N-acetylgalactosamine 3-beta-galactosyltransferase 1
C1orf112	chromosome 1 open reading frame 112
C1orf159	chromosome 1 open reading frame 159
C1orf174	chromosome 1 open reading frame 174
C1orf21	chromosome 1 open reading frame 21
C1orf226	chromosome 1 open reading frame 226
C1QTNF5	C1q and TNF related 5
C1QTNF9	C1q and TNF related 9
C1S	complement C1s
C20orf194	chromosome 20 open reading frame 194
C21orf91	chromosome 21 open reading frame 91
C22orf42	chromosome 22 open reading frame 42
C2CD2	C2 calcium dependent domain containing 2
C2CD5	C2 calcium dependent domain containing 5
C2orf69	chromosome 2 open reading frame 69
C2orf71	
C2orf88	chromosome 2 open reading frame 88
C2orf91	chromosome 2 open reading frame 91
C3orf38	chromosome 3 open reading frame 38
C3orf52	chromosome 3 open reading frame 52
C3orf58	chromosome 3 open reading frame 58
C3orf80	chromosome 3 open reading frame 80
C4orf19	chromosome 4 open reading frame 19
C4orf3	chromosome 4 open reading frame 3

C5orf24	chromosome 5 open reading frame 24
C5orf51	chromosome 5 open reading frame 51
C6orf120	chromosome 6 open reading frame 120
C6orf201	chromosome 6 open reading frame 201
C6orf223	chromosome 6 open reading frame 223
C7orf31	chromosome 7 open reading frame 31
C7orf43	chromosome 7 open reading frame 43
C8orf37	chromosome 8 open reading frame 37
C8orf44	chromosome 8 open reading frame 44
C8orf58	chromosome 8 open reading frame 58
C9orf152	chromosome 9 open reading frame 152
C9orf153	chromosome 9 open reading frame 153
C9orf40	chromosome 9 open reading frame 40
C9orf72	chromosome 9 open reading frame 72
C9orf85	chromosome 9 open reading frame 85
CA12	carbonic anhydrase 12
CA2	carbonic anhydrase 2
CA6	carbonic anhydrase 6
CAAP1	caspase activity and apoptosis inhibitor 1
CABLES1	Cdk5 and Abl enzyme substrate 1
CACHD1	cache domain containing 1
CACNA1C	calcium voltage-gated channel subunit alpha1 C
CACNA1D	calcium voltage-gated channel subunit alpha1 D
CACNA1I	calcium voltage-gated channel subunit alpha1 I
CACNB2	calcium voltage-gated channel auxiliary subunit beta 2
CACNB4	calcium voltage-gated channel auxiliary subunit beta 4
CACUL1	CDK2 associated cullin domain 1
CADM2	cell adhesion molecule 2
CADPS	calcium dependent secretion activator
CALB2	calbindin 2
CALCOCO2	calcium binding and coiled-coil domain 2
CALCR	calcitonin receptor

CALM1	calmodulin 1
CALM3	calmodulin 3
CALN1	calneuron 1
CAMK1	calcium/calmodulin dependent protein kinase I
CAMK2D	calcium/calmodulin dependent protein kinase II delta
CAMK2N1	calcium/calmodulin dependent protein kinase II inhibitor 1
CAMK2N2	calcium/calmodulin dependent protein kinase II inhibitor 2
CAMK4	calcium/calmodulin dependent protein kinase IV
CAMSAP2	calmodulin regulated spectrin associated protein family member 2
CAMTA1	calmodulin binding transcription activator 1
CAND1	cullin associated and neddylation dissociated 1
CAP1	cyclase associated actin cytoskeleton regulatory protein 1
CAPN3	calpain 3
CAPN5	calpain 5
CAPN7	calpain 7
CAPRIN1	cell cycle associated protein 1
CAPRIN2	caprin family member 2
CAPZA1	capping actin protein of muscle Z-line subunit alpha 1
CARF	calcium responsive transcription factor
CARS	cysteinyl-tRNA synthetase
CASC3	CASC3, exon junction complex subunit
CASKIN1	CASK interacting protein 1
CASP3	caspase 3
CAST	calpastatin
CASZ1	castor zinc finger 1
CAT	catalase
CATSPERG	cation channel sperm associated auxiliary subunit gamma
CBFA2T3	CBFA2/RUNX1 translocation partner 3
CBFB	core-binding factor subunit beta
CBL	Cbl proto-oncogene
CBLB	Cbl proto-oncogene B
CBR4	carbonyl reductase 4

CBX2	chromobox 2
CBX3	chromobox 3
CBX5	chromobox 5
CC2D1A	coiled-coil and C2 domain containing 1A
CCDC117	coiled-coil domain containing 117
CCDC120	coiled-coil domain containing 120
CCDC14	coiled-coil domain containing 14
CCDC141	coiled-coil domain containing 141
CCDC144NL	coiled-coil domain containing 144 family, N-terminal like
CCDC146	coiled-coil domain containing 146
CCDC148	coiled-coil domain containing 148
CCDC149	coiled-coil domain containing 149
CCDC28A	coiled-coil domain containing 28A
CCDC38	coiled-coil domain containing 38
CCDC43	coiled-coil domain containing 43
CCDC50	coiled-coil domain containing 50
CCDC6	coiled-coil domain containing 6
CCDC68	coiled-coil domain containing 68
CCDC7	coiled-coil domain containing 7
CCDC71L	coiled-coil domain containing 71 like
CCDC82	coiled-coil domain containing 82
CCDC93	coiled-coil domain containing 93
CCDC97	coiled-coil domain containing 97
CCL19	C-C motif chemokine ligand 19
CCL7	C-C motif chemokine ligand 7
CCNA1	cyclin A1
CCNA2	cyclin A2
CCNC	cyclin C
CCND1	cyclin D1
CCND2	cyclin D2
CCNE2	cyclin E2
CCNF	cyclin F

CCNG1	cyclin G1
CCNJ	cyclin J
CCNJL	cyclin J like
CCNK	cyclin K
CCNT2	cyclin T2
CCNY	cyclin Y
CCNYL1	cyclin Y like 1
CCR7	C-C motif chemokine receptor 7
CCSAP	centriole, cilia and spindle associated protein
CCSER1	coiled-coil serine rich protein 1
CCSER2	coiled-coil serine rich protein 2
CD200	CD200 molecule
CD200R1	CD200 receptor 1
CD276	CD276 molecule
CD28	CD28 molecule
CD300LG	CD300 molecule like family member g
CD55	CD55 molecule (Cromer blood group)
CD59	CD59 molecule (CD59 blood group)
CD80	CD80 molecule
CD86	CD86 molecule
CDC14B	cell division cycle 14B
CDC25A	cell division cycle 25A
CDC34	cell division cycle 34
CDC37L1	cell division cycle 37 like 1
CDC40	cell division cycle 40
CDC42	cell division cycle 42
CDC42BPA	CDC42 binding protein kinase alpha
CDC42SE1	CDC42 small effector 1
CDC6	cell division cycle 6
CDCA5	cell division cycle associated 5
CDCA7	cell division cycle associated 7
CDH1	cadherin 1

CDH11	cadherin 11
CDH13	cadherin 13
CDH20	cadherin 20
CDH26	cadherin 26
CDH8	cadherin 8
CDK14	cyclin dependent kinase 14
CDK15	cyclin dependent kinase 15
CDK17	cyclin dependent kinase 17
CDK19	cyclin dependent kinase 19
CDK2	cyclin dependent kinase 2
CDK2AP1	cyclin dependent kinase 2 associated protein 1
CDK6	cyclin dependent kinase 6
CDK8	cyclin dependent kinase 8
CDKN1A	cyclin dependent kinase inhibitor 1A
CDON	cell adhesion associated, oncogene regulated
CDV3	CDV3 homolog
CDX2	caudal type homeobox 2
CDYL	chromodomain Y like
CEACAM1	carcinoembryonic antigen related cell adhesion molecule 1
CEBPG	CCAAT enhancer binding protein gamma
CELF1	CUGBP Elav-like family member 1
CELF3	CUGBP Elav-like family member 3
CELF6	CUGBP Elav-like family member 6
CELSR1	cadherin EGF LAG seven-pass G-type receptor 1
CENPQ	centromere protein Q
CEP120	centrosomal protein 120
CEP128	centrosomal protein 128
CEP135	centrosomal protein 135
CEP164	centrosomal protein 164
CEP170B	centrosomal protein 170B
CEP350	centrosomal protein 350
CEP41	centrosomal protein 41

CEP44	centrosomal protein 44
CEP76	centrosomal protein 76
CEP85L	centrosomal protein 85 like
CEP95	centrosomal protein 95
CERCAM	cerebral endothelial cell adhesion molecule
CERS6	ceramide synthase 6
CFHR3	complement factor H related 3
CFL2	cofilin 2
CH25H	cholesterol 25-hydroxylase
CHAC1	ChaC glutathione specific gamma-glutamylcyclotransferase 1
CHAC2	ChaC cation transport regulator homolog 2
CHCHD3	coiled-coil-helix-coiled-coil-helix domain containing 3
CHD1	chromodomain helicase DNA binding protein 1
CHD3	chromodomain helicase DNA binding protein 3
CHD4	chromodomain helicase DNA binding protein 4
CHD7	chromodomain helicase DNA binding protein 7
CHD9	chromodomain helicase DNA binding protein 9
CHEK1	checkpoint kinase 1
CHI3L1	chitinase 3 like 1
CHIA	chitinase, acidic
CHIC1	cysteine rich hydrophobic domain 1
CHL1	cell adhesion molecule L1 like
CHN2	chimerin 2
CHORDC1	cysteine and histidine rich domain containing 1
CHRDL1	chordin like 1
CHRM2	cholinergic receptor muscarinic 2
CHRNA6	cholinergic receptor nicotinic alpha 6 subunit
CHRNA7	cholinergic receptor nicotinic alpha 7 subunit
CHST1	carbohydrate sulfotransferase 1
CHST11	carbohydrate sulfotransferase 11
CHST12	carbohydrate sulfotransferase 12
CHST2	carbohydrate sulfotransferase 2

CHST3	carbohydrate sulfotransferase 3
CHST6	carbohydrate sulfotransferase 6
CHSY1	chondroitin sulfate synthase 1
CHSY3	chondroitin sulfate synthase 3
CILP	cartilage intermediate layer protein
CISH	cytokine inducible SH2 containing protein
CITED2	Cbp/p300 interacting transactivator with Glu/Asp rich carboxy-terminal domain 2
CKAP4	cytoskeleton associated protein 4
CLASP1	cytoplasmic linker associated protein 1
CLCA2	chloride channel accessory 2
CLCC1	chloride channel CLIC like 1
CLCN3	chloride voltage-gated channel 3
CLCN5	chloride voltage-gated channel 5
CLDN1	claudin 1
CLDN12	claudin 12
CLDN16	claudin 16
CLEC7A	C-type lectin domain containing 7A
CLIC4	chloride intracellular channel 4
CLIP4	CAP-Gly domain containing linker protein family member 4
CLLU1	chronic lymphocytic leukemia up-regulated 1
CLN5	CLN5, intracellular trafficking protein
CLN8	CLN8, transmembrane ER and ERGIC protein
CLOCK	clock circadian regulator
CLP1	cleavage and polyadenylation factor I subunit 1
CLTC	clathrin heavy chain
CMPK1	cytidine/uridine monophosphate kinase 1
CNEP1R1	CTD nuclear envelope phosphatase 1 regulatory subunit 1
CNGB3	cyclic nucleotide gated channel beta 3
CNIH3	cornichon family AMPA receptor auxiliary protein 3
CNKSR2	connector enhancer of kinase suppressor of Ras 2
CNKSR3	CNKSR family member 3
CNN3	calponin 3

CNNM2	cyclin and CBS domain divalent metal cation transport mediator 2
CNOT2	CCR4-NOT transcription complex subunit 2
CNOT4	CCR4-NOT transcription complex subunit 4
CNOT6	CCR4-NOT transcription complex subunit 6
CNOT7	CCR4-NOT transcription complex subunit 7
CNST	consortin, connexin sorting protein
CNTD1	cyclin N-terminal domain containing 1
CNTFR	ciliary neurotrophic factor receptor
CNTN4	contactin 4
CNTN6	contactin 6
CNTNAP2	contactin associated protein like 2
CNTNAP3	contactin associated protein like 3
CNTRL	centriolin
COIL	coilin
COL10A1	collagen type X alpha 1 chain
COL11A1	collagen type XI alpha 1 chain
COL12A1	collagen type XII alpha 1 chain
COL13A1	collagen type XIII alpha 1 chain
COL15A1	collagen type XV alpha 1 chain
COL1A1	collagen type I alpha 1 chain
COL1A2	collagen type I alpha 2 chain
COL22A1	collagen type XXII alpha 1 chain
COL24A1	collagen type XXIV alpha 1 chain
COL25A1	collagen type XXV alpha 1 chain
COL27A1	collagen type XXVII alpha 1 chain
COL3A1	collagen type III alpha 1 chain
COL4A1	collagen type IV alpha 1 chain
COL4A2	collagen type IV alpha 2 chain
COL4A3BP	collagen type IV alpha 3 binding protein
COL4A5	collagen type IV alpha 5 chain
COL4A6	collagen type IV alpha 6 chain
COL5A2	collagen type V alpha 2 chain

COL6A3	collagen type VI alpha 3 chain
COL6A6	collagen type VI alpha 6 chain
COL9A3	collagen type IX alpha 3 chain
COMMD2	COMM domain containing 2
COMMD3	COMM domain containing 3
COMMD8	COMM domain containing 8
COPS2	COP9 signalosome subunit 2
COPS8	COP9 signalosome subunit 8
CORIN	corin, serine peptidase
CORO1C	coronin 1C
CORO7	coronin 7
COTL1	coactosin like F-actin binding protein 1
COX18	cytochrome c oxidase assembly factor COX18
COX8C	cytochrome c oxidase subunit 8C
CPA4	carboxypeptidase A4
CPD	carboxypeptidase D
CPE	carboxypeptidase E
CPEB3	cytoplasmic polyadenylation element binding protein 3
CPEB4	cytoplasmic polyadenylation element binding protein 4
CPED1	cadherin like and PC-esterase domain containing 1
CPLX2	complexin 2
CPM	carboxypeptidase M
CPNE8	copine 8
CPSF2	cleavage and polyadenylation specific factor 2
CPSF7	cleavage and polyadenylation specific factor 7
CRCP	CGRP receptor component
CRCT1	cysteine rich C-terminal 1
CREB1	cAMP responsive element binding protein 1
CREB5	cAMP responsive element binding protein 5
CREBBP	CREB binding protein
CREBL2	cAMP responsive element binding protein like 2
CREBZF	CREB/ATF bZIP transcription factor

CREG1	cellular repressor of E1A stimulated genes 1
CREG2	cellular repressor of E1A stimulated genes 2
CRIM1	cysteine rich transmembrane BMP regulator 1
CRISPLD1	cysteine rich secretory protein LCCL domain containing 1
CRK	CRK proto-oncogene, adaptor protein
CRKL	CRK like proto-oncogene, adaptor protein
CRTAM	cytotoxic and regulatory T cell molecule
CRTAP	cartilage associated protein
CRYBG3	crystallin beta-gamma domain containing 3
CS	citrate synthase
CSAD	cysteine sulfinic acid decarboxylase
CSDE1	cold shock domain containing E1
CSGALNACT1	chondroitin sulfate N-acetylgalactosaminyltransferase 1
CSMD1	CUB and Sushi multiple domains 1
CSNK1A1	casein kinase 1 alpha 1
CSNK1G1	casein kinase 1 gamma 1
CSNK2A1	casein kinase 2 alpha 1
CSRNP3	cysteine and serine rich nuclear protein 3
CSTA	cystatin A
CSTF2	cleavage stimulation factor subunit 2
CT62	cancer/testis antigen 62
CTAGE1	cutaneous T cell lymphoma-associated antigen 1
CTBP2	C-terminal binding protein 2
CTDSP2	CTD small phosphatase 2
CTDSPL2	CTD small phosphatase like 2
CTGF	connective tissue growth factor
CTHRC1	collagen triple helix repeat containing 1
CTNND2	catenin delta 2
CTNS	cystinosin, lysosomal cystine transporter
CTPS1	CTP synthase 1
CTPS2	CTP synthase 2
CTSA	cathepsin A

CTSC	cathepsin C
CTTNBP2NL	CTTNBP2 N-terminal like
CTXN3	cortexin 3
CUL2	cullin 2
CXCL14	C-X-C motif chemokine ligand 14
CXCL6	C-X-C motif chemokine ligand 6
CXCL9	C-X-C motif chemokine ligand 9
CXCR2	C-X-C motif chemokine receptor 2
CXCR4	C-X-C motif chemokine receptor 4
CXorf58	chromosome X open reading frame 58
CXXC4	CXXC finger protein 4
CYB561	cytochrome b561
CYB561D1	cytochrome b561 family member D1
CYCS	cytochrome c, somatic
CYLC2	cylicin 2
CYP19A1	cytochrome P450 family 19 subfamily A member 1
CYP20A1	cytochrome P450 family 20 subfamily A member 1
CYP24A1	cytochrome P450 family 24 subfamily A member 1
CYP2C8	cytochrome P450 family 2 subfamily C member 8
CYP2R1	cytochrome P450 family 2 subfamily R member 1
CYP46A1	cytochrome P450 family 46 subfamily A member 1
CYP4B1	cytochrome P450 family 4 subfamily B member 1
CYP4F2	cytochrome P450 family 4 subfamily F member 2
CYP4F3	cytochrome P450 family 4 subfamily F member 3
CYSLTR1	cysteinyl leukotriene receptor 1
CYTH1	cytohesin 1
CYTH3	cytohesin 3
CYYR1	cysteine and tyrosine rich 1
DAAM1	dishevelled associated activator of morphogenesis 1
DAAM2	dishevelled associated activator of morphogenesis 2
DAB1	DAB1, reelin adaptor protein
DAB2	DAB2, clathrin adaptor protein

DAG1	dystroglycan 1
DAGLA	diacylglycerol lipase alpha
DARS	aspartyl-tRNA synthetase
DAZ2	deleted in azoospermia 2
DAZ3	deleted in azoospermia 3
DAZAP2	DAZ associated protein 2
DAZL	deleted in azoospermia like
DBF4	DBF4 zinc finger
DBR1	debranching RNA lariats 1
DCAF12L1	DDB1 and CUL4 associated factor 12 like 1
DCAF15	DDB1 and CUL4 associated factor 15
DCAF4L1	DDB1 and CUL4 associated factor 4 like 1
DCAF4L2	DDB1 and CUL4 associated factor 4 like 2
DCAF5	DDB1 and CUL4 associated factor 5
DCAF6	DDB1 and CUL4 associated factor 6
DCAF8	DDB1 and CUL4 associated factor 8
DCBLD1	discoidin, CUB and LCCL domain containing 1
DCBLD2	discoidin, CUB and LCCL domain containing 2
DCDC2	doublecortin domain containing 2
DCLK3	doublecortin like kinase 3
DCLRE1B	DNA cross-link repair 1B
DCLRE1C	DNA cross-link repair 1C
DCP2	decapping mRNA 2
DCUN1D1	defective in cullin neddylation 1 domain containing 1
DCUN1D3	defective in cullin neddylation 1 domain containing 3
DCUN1D4	defective in cullin neddylation 1 domain containing 4
DCX	doublecortin
DDAH1	dimethylarginine dimethylaminohydrolase 1
DDHD1	DDHD domain containing 1
DDHD2	DDHD domain containing 2
DDI2	DNA damage inducible 1 homolog 2
DDIT4	DNA damage inducible transcript 4

DDTL	D-dopachrome tautomerase like
DDX17	DEAD-box helicase 17
DDX18	DEAD-box helicase 18
DDX19A	DEAD-box helicase 19A
DDX19B	DEAD-box helicase 19B
DDX20	DEAD-box helicase 20
DDX46	DEAD-box helicase 46
DDX5	DEAD-box helicase 5
DDX53	DEAD-box helicase 53
DDX55	DEAD-box helicase 55
DECR2	2,4-dienoyl-CoA reductase 2
DEFB119	defensin beta 119
DEFB132	defensin beta 132
DENND2C	DENN domain containing 2C
DENND4C	DENN domain containing 4C
DENND6A	DENN domain containing 6A
DEPDC1	DEP domain containing 1
DERL1	derlin 1
DESI2	desumoylating isopeptidase 2
DEXI	Dexi homolog
DFFB	DNA fragmentation factor subunit beta
DGKA	diacylglycerol kinase alpha
DGKE	diacylglycerol kinase epsilon
DGKH	diacylglycerol kinase eta
DGKZ	diacylglycerol kinase zeta
DHX15	DEAH-box helicase 15
DHX36	DEAH-box helicase 36
DIABLO	diablo IAP-binding mitochondrial protein
DIAPH2	diaphanous related formin 2
DIAPH3	diaphanous related formin 3
DICER1	dicer 1, ribonuclease III
DIEXF	

DIMT1	DIM1 dimethyladenosine transferase 1 homolog
DIP2B	disco interacting protein 2 homolog B
DIRAS2	DIRAS family GTPase 2
DKK1	dickkopf WNT signaling pathway inhibitor 1
DLC1	DLC1 Rho GTPase activating protein
DLG2	discs large MAGUK scaffold protein 2
DLG4	discs large MAGUK scaffold protein 4
DLGAP2	DLG associated protein 2
DLGAP4	DLG associated protein 4
DLL4	delta like canonical Notch ligand 4
DLST	dihydrolipoamide S-succinyltransferase
DMD	dystrophin
DMP1	dentin matrix acidic phosphoprotein 1
DMRT2	doublesex and mab-3 related transcription factor 2
DNA2	DNA replication helicase/nuclease 2
DNAJA2	DnaJ heat shock protein family (Hsp40) member A2
DNAJB9	DnaJ heat shock protein family (Hsp40) member B9
DNAJC13	DnaJ heat shock protein family (Hsp40) member C13
DNAJC21	DnaJ heat shock protein family (Hsp40) member C21
DNAJC25	DnaJ heat shock protein family (Hsp40) member C25
DNAJC25-GNG10	DNAJC25-GNG10 readthrough
DNAJC3	DnaJ heat shock protein family (Hsp40) member C3
DNAJC5	DnaJ heat shock protein family (Hsp40) member C5
DNAL1	dynein axonemal light chain 1
DNASE2B	deoxyribonuclease 2 beta
DNM3	dynamin 3
DNMBP	dynamin binding protein
DNMT3A	DNA methyltransferase 3 alpha
DOC2A	double C2 domain alpha
DOCK5	dedicator of cytokinesis 5
DOCK7	dedicator of cytokinesis 7
DOK3	docking protein 3

DOK5	docking protein 5
DOLPP1	dolichyldiphosphatase 1
DPAGT1	dolichyl-phosphate N-acetylglucosaminephosphotransferase 1
DPH1	diphthamide biosynthesis 1
DPH3	diphthamide biosynthesis 3
DPY19L1	dpy-19 like C-mannosyltransferase 1
DPY19L3	dpy-19 like C-mannosyltransferase 3
DPYSL2	dihydropyrimidinase like 2
DPYSL5	dihydropyrimidinase like 5
DRAM1	DNA damage regulated autophagy modulator 1
DRD1	dopamine receptor D1
DRP2	dystrophin related protein 2
DSC3	desmocollin 3
DSG1	desmoglein 1
DSG3	desmoglein 3
DST	dystonin
DTD2	D-tyrosyl-tRNA deacylase 2 (putative)
DTNA	dystrobrevin alpha
DTX2	deltex E3 ubiquitin ligase 2
DTX4	deltex E3 ubiquitin ligase 4
DUOX1	dual oxidase 1
DUSP1	dual specificity phosphatase 1
DUSP16	dual specificity phosphatase 16
DUSP22	dual specificity phosphatase 22
DUSP4	dual specificity phosphatase 4
DUSP6	dual specificity phosphatase 6
DUSP9	dual specificity phosphatase 9
DUT	deoxyuridine triphosphatase
DVL3	dishevelled segment polarity protein 3
DYNAP	dynactin associated protein
DYNC1LI2	dynein cytoplasmic 1 light intermediate chain 2
DYNC2LI1	dynein cytoplasmic 2 light intermediate chain 1

DYRK1A	dual specificity tyrosine phosphorylation regulated kinase 1A
DYRK2	dual specificity tyrosine phosphorylation regulated kinase 2
DZIP1	DAZ interacting zinc finger protein 1
E2F2	E2F transcription factor 2
E2F3	E2F transcription factor 3
E2F5	E2F transcription factor 5
E2F6	E2F transcription factor 6
E2F7	E2F transcription factor 7
EAF1	ELL associated factor 1
EBPL	EBP like
ECHDC2	enoyl-CoA hydratase domain containing 2
EDEM1	ER degradation enhancing alpha-mannosidase like protein 1
EDEM3	ER degradation enhancing alpha-mannosidase like protein 3
EDN1	endothelin 1
EEA1	early endosome antigen 1
EED	embryonic ectoderm development
EEF2K	eukaryotic elongation factor 2 kinase
EEPD1	endonuclease/exonuclease/phosphatase family domain containing 1
EFCAB14	EF-hand calcium binding domain 14
EFHD2	EF-hand domain family member D2
EFNA1	ephrin A1
EFNA3	ephrin A3
EFNB2	ephrin B2
EFR3A	EFR3 homolog A
EGFR	epidermal growth factor receptor
EGLN1	egl-9 family hypoxia inducible factor 1
EGLN2	egl-9 family hypoxia inducible factor 2
EGR2	early growth response 2
EHF	ETS homologous factor
EIF1AX	eukaryotic translation initiation factor 1A X-linked
EIF2AK2	eukaryotic translation initiation factor 2 alpha kinase 2
EIF2S1	eukaryotic translation initiation factor 2 subunit alpha

EIF3J	eukaryotic translation initiation factor 3 subunit J
EIF4E	eukaryotic translation initiation factor 4E
EIF4E3	eukaryotic translation initiation factor 4E family member 3
EIF4ENIF1	eukaryotic translation initiation factor 4E nuclear import factor 1
EIF4G2	eukaryotic translation initiation factor 4 gamma 2
EIF5A2	eukaryotic translation initiation factor 5A2
EIF5B	eukaryotic translation initiation factor 5B
ELAVL2	ELAV like RNA binding protein 2
ELAVL3	ELAV like RNA binding protein 3
ELAVL4	ELAV like RNA binding protein 4
ELF2	E74 like ETS transcription factor 2
ELF4	E74 like ETS transcription factor 4
ELFN2	extracellular leucine rich repeat and fibronectin type III domain containing 2
ELK3	ELK3, ETS transcription factor
ELK4	ELK4, ETS transcription factor
ELL2	elongation factor for RNA polymerase II 2
ELMOD1	ELMO domain containing 1
ELMOD2	ELMO domain containing 2
ELMSAN1	ELM2 and Myb/SANT domain containing 1
ELN	elastin
ELOVL2	ELOVL fatty acid elongase 2
ELOVL4	ELOVL fatty acid elongase 4
ELOVL5	ELOVL fatty acid elongase 5
EMCN	endomucin
EML1	echinoderm microtubule associated protein like 1
EML4	echinoderm microtubule associated protein like 4
EMX2	empty spiracles homeobox 2
EN2	engrailed homeobox 2
ENAH	ENAH, actin regulator
ENOX2	ecto-NOX disulfide-thiol exchanger 2
ENPEP	glutamyl aminopeptidase
ENPP5	ectonucleotide pyrophosphatase/phosphodiesterase 5 (putative)

ENTPD1	ectonucleoside triphosphate diphosphohydrolase 1
ENY2	ENY2, transcription and export complex 2 subunit
EOGT	EGF domain specific O-linked N-acetylglucosamine transferase
EP400	E1A binding protein p400
EPB41	erythrocyte membrane protein band 4.1
EPB41L1	erythrocyte membrane protein band 4.1 like 1
EPB41L4A	erythrocyte membrane protein band 4.1 like 4A
EPB41L4B	erythrocyte membrane protein band 4.1 like 4B
EPB41L5	erythrocyte membrane protein band 4.1 like 5
EPC1	enhancer of polycomb homolog 1
EPDR1	ependymin related 1
EPG5	ectopic P-granules autophagy protein 5 homolog
EPHA2	EPH receptor A2
EPHA3	EPH receptor A3
EPHA4	EPH receptor A4
EPHA5	EPH receptor A5
EPHA7	EPH receptor A7
EPHB1	EPH receptor B1
EPHB2	EPH receptor B2
EPPIN	epididymal peptidase inhibitor
EPS15	epidermal growth factor receptor pathway substrate 15
EPS8	epidermal growth factor receptor pathway substrate 8
ERBB3	erb-b2 receptor tyrosine kinase 3
ERBB4	erb-b2 receptor tyrosine kinase 4
ERCC4	ERCC excision repair 4, endonuclease catalytic subunit
ERCC6	ERCC excision repair 6, chromatin remodeling factor
ERCC6L2	ERCC excision repair 6 like 2
EREG	epiregulin
ERG	ETS transcription factor ERG
ERI1	exoribonuclease 1
ERI2	ERI1 exoribonuclease family member 2
ERLIN1	ER lipid raft associated 1

ERMP1	endoplasmic reticulum metallopeptidase 1
ERP29	endoplasmic reticulum protein 29
ERRFI1	ERBB receptor feedback inhibitor 1
ERVFRD-1	endogenous retrovirus group FRD member 1, envelope
ESCO1	establishment of sister chromatid cohesion N-acetyltransferase 1
ESM1	endothelial cell specific molecule 1
ESPL1	extra spindle pole bodies like 1, separase
ESR1	estrogen receptor 1
ESR2	estrogen receptor 2
ESRP1	epithelial splicing regulatory protein 1
ESRP2	epithelial splicing regulatory protein 2
ESYT1	extended synaptotagmin 1
ETNK1	ethanolamine kinase 1
ETNK2	ethanolamine kinase 2
ETS1	ETS proto-oncogene 1, transcription factor
ETS2	ETS proto-oncogene 2, transcription factor
ETV1	ETS variant 1
ETV3	ETS variant 3
EVA1C	eva-1 homolog C
EVI2B	ecotropic viral integration site 2B
EVI5	ecotropic viral integration site 5
EVX2	even-skipped homeobox 2
EXOC6	exocyst complex component 6
EXOG	exo/endonuclease G
EXTL2	exostosin like glycosyltransferase 2
EYA2	EYA transcriptional coactivator and phosphatase 2
EYA3	EYA transcriptional coactivator and phosphatase 3
EYA4	EYA transcriptional coactivator and phosphatase 4
EZH1	enhancer of zeste 1 polycomb repressive complex 2 subunit
EZH2	enhancer of zeste 2 polycomb repressive complex 2 subunit
F9	coagulation factor IX
FA2H	fatty acid 2-hydroxylase

FAHD1	fumarylacetoacetate hydrolase domain containing 1
FAIM	Fas apoptotic inhibitory molecule
FAM102A	family with sequence similarity 102 member A
FAM103A1	
FAM104A	family with sequence similarity 104 member A
FAM107B	family with sequence similarity 107 member B
FAM109A	
FAM110B	family with sequence similarity 110 member B
FAM118A	family with sequence similarity 118 member A
FAM118B	family with sequence similarity 118 member B
FAM120C	family with sequence similarity 120C
FAM122A	family with sequence similarity 122A
FAM122C	family with sequence similarity 122C
FAM126A	family with sequence similarity 126 member A
FAM126B	family with sequence similarity 126 member B
FAM129A	family with sequence similarity 129 member A
FAM133A	family with sequence similarity 133 member A
FAM135A	family with sequence similarity 135 member A
FAM135B	family with sequence similarity 135 member B
FAM13A	family with sequence similarity 13 member A
FAM155A	family with sequence similarity 155 member A
FAM160B1	family with sequence similarity 160 member B1
FAM168A	family with sequence similarity 168 member A
FAM168B	family with sequence similarity 168 member B
FAM169A	family with sequence similarity 169 member A
FAM169B	family with sequence similarity 169 member B
FAM170B	family with sequence similarity 170 member B
FAM171A2	family with sequence similarity 171 member A2
FAM172A	family with sequence similarity 172 member A
FAM184B	family with sequence similarity 184 member B
FAM189A1	family with sequence similarity 189 member A1
FAM189A2	family with sequence similarity 189 member A2

FAM199X	family with sequence similarity 199, X-linked
FAM19A2	family with sequence similarity 19 member A2, C-C motif chemokine like
FAM19A4	family with sequence similarity 19 member A4, C-C motif chemokine like
FAM19A5	family with sequence similarity 19 member A5, C-C motif chemokine like
FAM208A	family with sequence similarity 208 member A
FAM210A	family with sequence similarity 210 member A
FAM210B	family with sequence similarity 210 member B
FAM214A	family with sequence similarity 214 member A
FAM214B	family with sequence similarity 214 member B
FAM217B	family with sequence similarity 217 member B
FAM218A	family with sequence similarity 218 member A
FAM219A	family with sequence similarity 219 member A
FAM219B	family with sequence similarity 219 member B
FAM221A	family with sequence similarity 221 member A
FAM222B	family with sequence similarity 222 member B
FAM24A	family with sequence similarity 24 member A
FAM43A	family with sequence similarity 43 member A
FAM46A	
FAM46D	
FAM49A	family with sequence similarity 49 member A
FAM49B	family with sequence similarity 49 member B
FAM53C	family with sequence similarity 53 member C
FAM69A	family with sequence similarity 69 member A
FAM71D	family with sequence similarity 71 member D
FAM72A	family with sequence similarity 72 member A
FAM72B	family with sequence similarity 72 member B
FAM81A	family with sequence similarity 81 member A
FAM83A	family with sequence similarity 83 member A
FAM84A	family with sequence similarity 84 member A
FAM8A1	family with sequence similarity 8 member A1
FAM91A1	family with sequence similarity 91 member A1
FAM96A	

FAM98A	family with sequence similarity 98 member A
FAM98B	family with sequence similarity 98 member B
FAM9B	family with sequence similarity 9 member B
FAM9C	family with sequence similarity 9 member C
FAN1	FANCD2 and FANCI associated nuclease 1
FANCE	FA complementation group E
FANCF	FA complementation group F
FAP	fibroblast activation protein alpha
FAR2	fatty acyl-CoA reductase 2
FARP1	FERM, ARH/RhoGEF and pleckstrin domain protein 1
FAS	Fas cell surface death receptor
FAXC	failed axon connections homolog
FBLN2	fibulin 2
FBP1	fructose-bisphosphatase 1
FBXL12	F-box and leucine rich repeat protein 12
FBXL14	F-box and leucine rich repeat protein 14
FBXL17	F-box and leucine rich repeat protein 17
FBXL19	F-box and leucine rich repeat protein 19
FBXL3	F-box and leucine rich repeat protein 3
FBXO10	F-box protein 10
FBXO11	F-box protein 11
FBXO15	F-box protein 15
FBXO22	F-box protein 22
FBXO28	F-box protein 28
FBXO30	F-box protein 30
FBXO32	F-box protein 32
FBXO33	F-box protein 33
FBXO39	F-box protein 39
FBXO45	F-box protein 45
FBXO48	F-box protein 48
FBXW11	F-box and WD repeat domain containing 11
FBXW2	F-box and WD repeat domain containing 2

FBXW7	F-box and WD repeat domain containing 7
FCHO2	FCH domain only 2
FCRL5	Fc receptor like 5
FDXR	ferredoxin reductase
FEM1C	fem-1 homolog C
FEN1	flap structure-specific endonuclease 1
FER	FER tyrosine kinase
FERMT2	fermitin family member 2
FEZ2	fasciculation and elongation protein zeta 2
FGD1	FYVE, RhoGEF and PH domain containing 1
FGD4	FYVE, RhoGEF and PH domain containing 4
FGD6	FYVE, RhoGEF and PH domain containing 6
FGF11	fibroblast growth factor 11
FGF20	fibroblast growth factor 20
FGF21	fibroblast growth factor 21
FGFR1	fibroblast growth factor receptor 1
FGL2	fibrinogen like 2
FHL1	four and a half LIM domains 1
FICD	FIC domain containing
FIGN	fidgetin, microtubule severing factor
FJX1	four jointed box 1
FKBP4	FK506 binding protein 4
FKTN	fukutin
FLI1	Fli-1 proto-oncogene, ETS transcription factor
FLII	FLII, actin remodeling protein
FLNA	filamin A
FLVCR1	feline leukemia virus subgroup C cellular receptor 1
FLVCR2	feline leukemia virus subgroup C cellular receptor family member 2
FMNL2	formin like 2
FMNL3	formin like 3
FMR1	fragile X mental retardation 1
FMR1NB	FMR1 neighbor

FNBP1L	formin binding protein 1 like
FNBP4	formin binding protein 4
FNDC3A	fibronectin type III domain containing 3A
FNDC3B	fibronectin type III domain containing 3B
FNIP1	folliculin interacting protein 1
FNIP2	folliculin interacting protein 2
FNTB	farnesyltransferase, CAAX box, beta
FOPNL	FGFR1OP N-terminal like
FOS	Fos proto-oncogene, AP-1 transcription factor subunit
FOSB	FosB proto-oncogene, AP-1 transcription factor subunit
FOSL2	FOS like 2, AP-1 transcription factor subunit
FOXA1	forkhead box A1
FOXF1	forkhead box F1
FOXG1	forkhead box G1
FOXK2	forkhead box K2
FOXO3	forkhead box O3
FOXP1	forkhead box P1
FOXP2	forkhead box P2
FOXR2	forkhead box R2
FRAS1	Fraser extracellular matrix complex subunit 1
FRAT1	FRAT1, WNT signaling pathway regulator
FRAT2	FRAT2, WNT signaling pathway regulator
FREM2	FRAS1 related extracellular matrix protein 2
FRMD4B	FERM domain containing 4B
FRMD5	FERM domain containing 5
FRMD6	FERM domain containing 6
FRS2	fibroblast growth factor receptor substrate 2
FRZB	frizzled related protein
FSD1L	fibronectin type III and SPRY domain containing 1 like
FSIP1	fibrous sheath interacting protein 1
FSTL1	follistatin like 1
FUBP1	far upstream element binding protein 1

FUBP3	far upstream element binding protein 3
FUCA1	alpha-L-fucosidase 1
FUT3	fucosyltransferase 3 (Lewis blood group)
FUT4	fucosyltransferase 4
FUT9	fucosyltransferase 9
FXR1	FMR1 autosomal homolog 1
FXR2	FMR1 autosomal homolog 2
FYCO1	FYVE and coiled-coil domain containing 1
FZD3	frizzled class receptor 3
FZD4	frizzled class receptor 4
G6PC	glucose-6-phosphatase catalytic subunit
G6PC2	glucose-6-phosphatase catalytic subunit 2
G6PD	glucose-6-phosphate dehydrogenase
GAB1	GRB2 associated binding protein 1
GAB3	GRB2 associated binding protein 3
GABBR2	gamma-aminobutyric acid type B receptor subunit 2
GABPA	GA binding protein transcription factor subunit alpha
GABPB1	GA binding protein transcription factor subunit beta 1
GABRA2	gamma-aminobutyric acid type A receptor alpha2 subunit
GABRA4	gamma-aminobutyric acid type A receptor alpha4 subunit
GABRA5	gamma-aminobutyric acid type A receptor alpha5 subunit
GABRB1	gamma-aminobutyric acid type A receptor beta1 subunit
GABRG1	gamma-aminobutyric acid type A receptor gamma1 subunit
GABRP	gamma-aminobutyric acid type A receptor pi subunit
GAD1	glutamate decarboxylase 1
GADL1	glutamate decarboxylase like 1
GAL3ST1	galactose-3-O-sulfotransferase 1
GALC	galactosylceramidase
GALE	UDP-galactose-4-epimerase
GALK2	galactokinase 2
GALNT1	polypeptide N-acetylgalactosaminyltransferase 1
GALNT10	polypeptide N-acetylgalactosaminyltransferase 10

GALNT13	polypeptide N-acetylgalactosaminyltransferase 13
GALNT15	polypeptide N-acetylgalactosaminyltransferase 15
GALNT16	polypeptide N-acetylgalactosaminyltransferase 16
GALNT2	polypeptide N-acetylgalactosaminyltransferase 2
GALNT3	polypeptide N-acetylgalactosaminyltransferase 3
GALNT4	polypeptide N-acetylgalactosaminyltransferase 4
GALNT7	polypeptide N-acetylgalactosaminyltransferase 7
GAN	gigaxonin
GANC	glucosidase alpha, neutral C
GAS2L3	growth arrest specific 2 like 3
GAS7	growth arrest specific 7
GATA4	GATA binding protein 4
GATA5	GATA binding protein 5
GATM	glycine amidinotransferase
GBP1	guanylate binding protein 1
GBP3	guanylate binding protein 3
GBP6	guanylate binding protein family member 6
GCH1	GTP cyclohydrolase 1
GCLC	glutamate-cysteine ligase catalytic subunit
GCNT4	glucosaminyl (N-acetyl) transferase 4, core 2
GCSAM	germinal center associated signaling and motility
GCSAML	germinal center associated signaling and motility like
GDAP1	ganglioside induced differentiation associated protein 1
GDAP2	ganglioside induced differentiation associated protein 2
GDF6	growth differentiation factor 6
GDPD1	glycerophosphodiester phosphodiesterase domain containing 1
GEMIN7	gem nuclear organelle associated protein 7
GFM1	G elongation factor mitochondrial 1
GFPT2	glutamine-fructose-6-phosphate transaminase 2
GGCT	gamma-glutamylcyclotransferase
GHR	growth hormone receptor
GIMAP1	GTPase, IMAP family member 1

GIMAP6	GTPase, IMAP family member 6
GIN1	gypsy retrotransposon integrase 1
GIPC1	GIPC PDZ domain containing family member 1
GIPC2	GIPC PDZ domain containing family member 2
GIT2	GIT ArfGAP 2
GJA1	gap junction protein alpha 1
GJC1	gap junction protein gamma 1
GK5	glycerol kinase 5
GLCCI1	glucocorticoid induced 1
GLDC	glycine decarboxylase
GLDN	gliomedin
GLI3	GLI family zinc finger 3
GLIS2	GLIS family zinc finger 2
GLMN	glomulin, FKBP associated protein
GLRX	glutaredoxin
GLUD1	glutamate dehydrogenase 1
GM2A	GM2 ganglioside activator
GMDS	GDP-mannose 4,6-dehydratase
GMNC	geminin coiled-coil domain containing
GNA13	G protein subunit alpha 13
GNAI2	G protein subunit alpha i2
GNAQ	G protein subunit alpha q
GNG10	G protein subunit gamma 10
GNG2	G protein subunit gamma 2
GNG5	G protein subunit gamma 5
GNPDA1	glucosamine-6-phosphate deaminase 1
GNPDA2	glucosamine-6-phosphate deaminase 2
GNPNAT1	glucosamine-phosphate N-acetyltransferase 1
GOLGA1	golgin A1
GOLGA4	golgin A4
GOLGA7	golgin A7
GOLGB1	golgin B1

GOLPH3L	golgi phosphoprotein 3 like
GOLT1B	golgi transport 1B
GOPC	golgi associated PDZ and coiled-coil motif containing
GOSR1	golgi SNAP receptor complex member 1
GOSR2	golgi SNAP receptor complex member 2
GPALPP1	GPALPP motifs containing 1
GPATCH2	G-patch domain containing 2
GPATCH2L	G-patch domain containing 2 like
GPATCH8	G-patch domain containing 8
GPC4	glypican 4
GPCPD1	glycerophosphocholine phosphodiesterase 1
GPD2	glycerol-3-phosphate dehydrogenase 2
GPER1	G protein-coupled estrogen receptor 1
GPM6A	glycoprotein M6A
GPR137	G protein-coupled receptor 137
GPR137C	G protein-coupled receptor 137C
GPR150	G protein-coupled receptor 150
GPR157	G protein-coupled receptor 157
GPR158	G protein-coupled receptor 158
GPR173	G protein-coupled receptor 173
GPR26	G protein-coupled receptor 26
GPR27	G protein-coupled receptor 27
GPR52	G protein-coupled receptor 52
GPR6	G protein-coupled receptor 6
GPR61	G protein-coupled receptor 61
GPR63	G protein-coupled receptor 63
GPR75-ASB3	GPR75-ASB3 readthrough
GPRIN3	GPRIN family member 3
GPX7	glutathione peroxidase 7
GPX8	glutathione peroxidase 8 (putative)
GRAMD1C	GRAM domain containing 1C
GRB10	growth factor receptor bound protein 10

GREB1	growth regulating estrogen receptor binding 1
GRHL2	grainyhead like transcription factor 2
GRHL3	grainyhead like transcription factor 3
GRIA2	glutamate ionotropic receptor AMPA type subunit 2
GRIA4	glutamate ionotropic receptor AMPA type subunit 4
GRID2IP	Grid2 interacting protein
GRIK2	glutamate ionotropic receptor kainate type subunit 2
GRIN2B	glutamate ionotropic receptor NMDA type subunit 2B
GRM6	glutamate metabotropic receptor 6
GRM7	glutamate metabotropic receptor 7
GRSF1	G-rich RNA sequence binding factor 1
GSAP	gamma-secretase activating protein
GSKIP	GSK3B interacting protein
GSPT1	G1 to S phase transition 1
GSR	glutathione-disulfide reductase
GSTA4	glutathione S-transferase alpha 4
GTDC1	glycosyltransferase like domain containing 1
GTF2A1	general transcription factor IIA subunit 1
GTF2E1	general transcription factor IIE subunit 1
GTF2E2	general transcription factor IIE subunit 2
GTF2H1	general transcription factor IIH subunit 1
GUCY2C	guanylate cyclase 2C
GXYLT1	glucoside xylosyltransferase 1
GYG2	glycogenin 2
GYPE	glycophorin E (MNS blood group)
GZF1	GDNF inducible zinc finger protein 1
HABP4	hyaluronan binding protein 4
HACE1	HECT domain and ankyrin repeat containing E3 ubiquitin protein ligase 1
HAND1	heart and neural crest derivatives expressed 1
HAND2	heart and neural crest derivatives expressed 2
HAO1	hydroxyacid oxidase 1
HAS2	hyaluronan synthase 2

HBP1	HMG-box transcription factor 1
HCAR1	hydroxycarboxylic acid receptor 1
HCFC2	host cell factor C2
HCN1	hyperpolarization activated cyclic nucleotide gated potassium channel 1
HDAC4	histone deacetylase 4
HDAC5	histone deacetylase 5
HDLBP	high density lipoprotein binding protein
HDX	highly divergent homeobox
HECTD2	HECT domain E3 ubiquitin protein ligase 2
HECTD4	HECT domain E3 ubiquitin protein ligase 4
HELLS	helicase, lymphoid specific
HELZ	helicase with zinc finger
HELZ2	helicase with zinc finger 2
HEPHL1	hephaestin like 1
HFE	homeostatic iron regulator
HGF	hepatocyte growth factor
HHLA3	HERV-H LTR-associating 3
HIC2	HIC ZBTB transcriptional repressor 2
HIF1A	hypoxia inducible factor 1 subunit alpha
HIF1AN	hypoxia inducible factor 1 subunit alpha inhibitor
HIP1	huntingtin interacting protein 1
HIP1R	huntingtin interacting protein 1 related
HIPK1	homeodomain interacting protein kinase 1
HIPK2	homeodomain interacting protein kinase 2
HIPK3	homeodomain interacting protein kinase 3
HLA-DPA1	major histocompatibility complex, class II, DP alpha 1
HLCS	holocarboxylase synthetase
HLX	H2.0 like homeobox
HMBOX1	homeobox containing 1
HMGA1	high mobility group AT-hook 1
HMGA2	high mobility group AT-hook 2
HMGB3	high mobility group box 3

HMGN1	high mobility group nucleosome binding domain 1
HNF4A	hepatocyte nuclear factor 4 alpha
HNRNPA1	heterogeneous nuclear ribonucleoprotein A1
HNRNPA3	heterogeneous nuclear ribonucleoprotein A3
HNRNPC	heterogeneous nuclear ribonucleoprotein C (C1/C2)
HNRNPD	heterogeneous nuclear ribonucleoprotein D
HNRNPH1	heterogeneous nuclear ribonucleoprotein H1
HNRNPK	heterogeneous nuclear ribonucleoprotein K
HNRNPLL	heterogeneous nuclear ribonucleoprotein L like
HNRNPU	heterogeneous nuclear ribonucleoprotein U
HOMER1	homer scaffold protein 1
HOMER2	homer scaffold protein 2
HOOK1	hook microtubule tethering protein 1
HORMAD1	HORMA domain containing 1
HOXA1	homeobox A1
HOXA11	homeobox A11
HOXA3	homeobox A3
HOXA4	homeobox A4
HOXA5	homeobox A5
HOXA9	homeobox A9
HOXB4	homeobox B4
HOXB8	homeobox B8
HOXC10	homeobox C10
HOXC4	homeobox C4
HOXC9	homeobox C9
HOXD1	homeobox D1
HOXD13	homeobox D13
HOXD3	homeobox D3
HP1BP3	heterochromatin protein 1 binding protein 3
HPGD	15-hydroxyprostaglandin dehydrogenase
HPS4	HPS4, biogenesis of lysosomal organelles complex 3 subunit 2
HPS5	HPS5, biogenesis of lysosomal organelles complex 2 subunit 2

HRK	harakiri, BCL2 interacting protein
HS2ST1	heparan sulfate 2-O-sulfotransferase 1
HS3ST1	heparan sulfate-glucosamine 3-sulfotransferase 1
HS3ST3A1	heparan sulfate-glucosamine 3-sulfotransferase 3A1
HS3ST3B1	heparan sulfate-glucosamine 3-sulfotransferase 3B1
HSD17B13	hydroxysteroid 17-beta dehydrogenase 13
HSD17B6	hydroxysteroid 17-beta dehydrogenase 6
HSPA12A	heat shock protein family A (Hsp70) member 12A
HSPA13	heat shock protein family A (Hsp70) member 13
HSPA14	heat shock protein family A (Hsp70) member 14
HSPA4L	heat shock protein family A (Hsp70) member 4 like
HSPA8	heat shock protein family A (Hsp70) member 8
HSPA9	heat shock protein family A (Hsp70) member 9
HSPD1	heat shock protein family D (Hsp60) member 1
HSPE1	heat shock protein family E (Hsp10) member 1
HTR1E	5-hydroxytryptamine receptor 1E
HTR1F	5-hydroxytryptamine receptor 1F
HTR4	5-hydroxytryptamine receptor 4
HTRA3	HtrA serine peptidase 3
IAH1	isoamyl acetate hydrolyzing esterase 1 (putative)
ICA1L	islet cell autoantigen 1 like
ICK	intestinal cell kinase
ICOS	inducible T cell costimulator
IDH1	isocitrate dehydrogenase (NADP(+)) 1, cytosolic
IDH2	isocitrate dehydrogenase (NADP(+)) 2, mitochondrial
IER5	immediate early response 5
IFI44L	interferon induced protein 44 like
IFIT5	interferon induced protein with tetratricopeptide repeats 5
IFITM10	interferon induced transmembrane protein 10
IFNG	interferon gamma
IFNGR2	interferon gamma receptor 2
IFNLR1	interferon lambda receptor 1

IFRD1	interferon related developmental regulator 1
IFT52	intraflagellar transport 52
IFT80	intraflagellar transport 80
IGDCC3	immunoglobulin superfamily DCC subclass member 3
IGDCC4	immunoglobulin superfamily DCC subclass member 4
IGF1	insulin like growth factor 1
IGF1R	insulin like growth factor 1 receptor
IGF2BP1	insulin like growth factor 2 mRNA binding protein 1
IGF2BP2	insulin like growth factor 2 mRNA binding protein 2
IGF2BP3	insulin like growth factor 2 mRNA binding protein 3
IGFL1	IGF like family member 1
IGHMBP2	immunoglobulin mu DNA binding protein 2
IGLON5	IgLON family member 5
IGSF1	immunoglobulin superfamily member 1
IGSF10	immunoglobulin superfamily member 10
IGSF3	immunoglobulin superfamily member 3
IKBKB	inhibitor of nuclear factor kappa B kinase subunit beta
IKZF2	IKAROS family zinc finger 2
IKZF4	IKAROS family zinc finger 4
IL10	interleukin 10
IL10RB	interleukin 10 receptor subunit beta
IL11RA	interleukin 11 receptor subunit alpha
IL12RB2	interleukin 12 receptor subunit beta 2
IL13	interleukin 13
IL18R1	interleukin 18 receptor 1
IL1A	interleukin 1 alpha
IL1RAPL2	interleukin 1 receptor accessory protein like 2
IL22RA1	interleukin 22 receptor subunit alpha 1
IL2RA	interleukin 2 receptor subunit alpha
IL36G	interleukin 36 gamma
IL6R	interleukin 6 receptor
ILDR2	immunoglobulin like domain containing receptor 2

IMPACT	impact RWD domain protein
IMPDH1	inosine monophosphate dehydrogenase 1
IMPG2	interphotoreceptor matrix proteoglycan 2
ING2	inhibitor of growth family member 2
ING3	inhibitor of growth family member 3
ING5	inhibitor of growth family member 5
INHBB	inhibin subunit beta B
INMT	indolethylamine N-methyltransferase
INO80D	INO80 complex subunit D
INPP5F	inositol polyphosphate-5-phosphatase F
INSIG2	insulin induced gene 2
INSL4	insulin like 4
INSM2	INSM transcriptional repressor 2
INSR	insulin receptor
INTS2	integrator complex subunit 2
INTS6	integrator complex subunit 6
INTS8	integrator complex subunit 8
IP6K3	inositol hexakisphosphate kinase 3
IPO7	importin 7
IPPK	inositol-pentakisphosphate 2-kinase
IQCB1	IQ motif containing B1
IRF1	interferon regulatory factor 1
IRF2BPL	interferon regulatory factor 2 binding protein like
IRF4	interferon regulatory factor 4
IRGQ	immunity related GTPase Q
IRS1	insulin receptor substrate 1
IRS2	insulin receptor substrate 2
IRX4	iroquois homeobox 4
ISM1	isthmin 1
ITCH	itchy E3 ubiquitin protein ligase
ITFG2	integrin alpha FG-GAP repeat containing 2
ITGA1	integrin subunit alpha 1

ITGA10	integrin subunit alpha 10
ITGA4	integrin subunit alpha 4
ITGA7	integrin subunit alpha 7
ITGA8	integrin subunit alpha 8
ITGAV	integrin subunit alpha V
ITGB1	integrin subunit beta 1
ITGB3	integrin subunit beta 3
ITGB6	integrin subunit beta 6
ITGB8	integrin subunit beta 8
ITK	IL2 inducible T cell kinase
ITM2B	integral membrane protein 2B
ITPK1	inositol-tetrakisphosphate 1-kinase
ITPR2	inositol 1,4,5-trisphosphate receptor type 2
ITSN1	intersectin 1
IYD	iodotyrosine deiodinase
JAK1	Janus kinase 1
JAKMIP2	janus kinase and microtubule interacting protein 2
JAKMIP3	Janus kinase and microtubule interacting protein 3
JARID2	jumonji and AT-rich interaction domain containing 2
JAZF1	JAZF zinc finger 1
JDP2	Jun dimerization protein 2
JKAMP	JNK1/MAPK8 associated membrane protein
JMY	junction mediating and regulatory protein, p53 cofactor
JOSD1	Josephin domain containing 1
JOSD2	Josephin domain containing 2
JPH4	junctophilin 4
JUN	Jun proto-oncogene, AP-1 transcription factor subunit
JUNB	JunB proto-oncogene, AP-1 transcription factor subunit
KANK3	KN motif and ankyrin repeat domains 3
KANK4	KN motif and ankyrin repeat domains 4
KAT2B	lysine acetyltransferase 2B
KAT6A	lysine acetyltransferase 6A

KAT6B	lysine acetyltransferase 6B
KATNAL1	katanin catalytic subunit A1 like 1
KATNBL1	katanin regulatory subunit B1 like 1
KBTBD6	kelch repeat and BTB domain containing 6
KCMF1	potassium channel modulatory factor 1
KCNA4	potassium voltage-gated channel subfamily A member 4
KCNA5	potassium voltage-gated channel subfamily A member 5
KCNA6	potassium voltage-gated channel subfamily A member 6
KCNB1	potassium voltage-gated channel subfamily B member 1
KCNC1	potassium voltage-gated channel subfamily C member 1
KCNC2	potassium voltage-gated channel subfamily C member 2
KCND2	potassium voltage-gated channel subfamily D member 2
KCNE3	potassium voltage-gated channel subfamily E regulatory subunit 3
KCNIP3	potassium voltage-gated channel interacting protein 3
KCNIP4	potassium voltage-gated channel interacting protein 4
KCNJ10	potassium voltage-gated channel subfamily J member 10
KCNJ12	potassium voltage-gated channel subfamily J member 12
KCNJ13	potassium voltage-gated channel subfamily J member 13
KCNJ15	potassium voltage-gated channel subfamily J member 15
KCNJ2	potassium voltage-gated channel subfamily J member 2
KCNJ3	potassium voltage-gated channel subfamily J member 3
KCNK1	potassium two pore domain channel subfamily K member 1
KCNK12	potassium two pore domain channel subfamily K member 12
KCNK5	potassium two pore domain channel subfamily K member 5
KCNMB1	potassium calcium-activated channel subfamily M regulatory beta subunit 1
KCNMB2	potassium calcium-activated channel subfamily M regulatory beta subunit 2
KCNQ4	potassium voltage-gated channel subfamily Q member 4
KCNS2	potassium voltage-gated channel modifier subfamily S member 2
KCNT1	potassium sodium-activated channel subfamily T member 1
KCTD10	potassium channel tetramerization domain containing 10
KCTD14	potassium channel tetramerization domain containing 14
KCTD16	potassium channel tetramerization domain containing 16

KCTD17	potassium channel tetramerization domain containing 17
KCTD18	potassium channel tetramerization domain containing 18
KCTD19	potassium channel tetramerization domain containing 19
KCTD2	potassium channel tetramerization domain containing 2
KCTD21	potassium channel tetramerization domain containing 21
KCTD7	potassium channel tetramerization domain containing 7
KCTD8	potassium channel tetramerization domain containing 8
KCTD9	potassium channel tetramerization domain containing 9
KDELC1	KDEL motif containing 1
KDM3A	lysine demethylase 3A
KDM3B	lysine demethylase 3B
KDR	kinase insert domain receptor
KHDRBS1	KH RNA binding domain containing, signal transduction associated 1
KHK	ketohexokinase
KHNYN	KH and NYN domain containing
KIAA0355	KIAA0355
KIAA0754	KIAA0754
KIAA0895	KIAA0895
KIAA0930	KIAA0930
KIAA1024	
KIAA1147	KIAA1147
KIAA1328	KIAA1328
KIAA1468	
KIAA1522	KIAA1522
KIAA1549	KIAA1549
KIAA1958	KIAA1958
KIAA2013	KIAA2013
KIDINS220	kinase D interacting substrate 220
KIF11	kinesin family member 11
KIF13A	kinesin family member 13A
KIF14	kinesin family member 14
KIF1B	kinesin family member 1B

KIF24	kinesin family member 24
KIF2A	kinesin family member 2A
KIF3A	kinesin family member 3A
KIF3B	kinesin family member 3B
KIN	Kin17 DNA and RNA binding protein
KLF10	Kruppel like factor 10
KLF11	Kruppel like factor 11
KLF12	Kruppel like factor 12
KLF13	Kruppel like factor 13
KLF3	Kruppel like factor 3
KLF4	Kruppel like factor 4
KLF8	Kruppel like factor 8
KLF9	Kruppel like factor 9
KLHDC8B	kelch domain containing 8B
KLHL14	kelch like family member 14
KLHL15	kelch like family member 15
KLHL18	kelch like family member 18
KLHL2	kelch like family member 2
KLHL20	kelch like family member 20
KLHL23	kelch like family member 23
KLHL28	kelch like family member 28
KLHL3	kelch like family member 3
KLHL31	kelch like family member 31
KLHL34	kelch like family member 34
KLHL42	kelch like family member 42
KLHL5	kelch like family member 5
KLHL6	kelch like family member 6
KLRF1	killer cell lectin like receptor F1
KMT2A	lysine methyltransferase 2A
KMT2C	lysine methyltransferase 2C
KMT2D	lysine methyltransferase 2D
KMT2E	lysine methyltransferase 2E

KPNA1	karyopherin subunit alpha 1
KPNA3	karyopherin subunit alpha 3
KPNA4	karyopherin subunit alpha 4
KPNA5	karyopherin subunit alpha 5
KPNA6	karyopherin subunit alpha 6
KRAS	KRAS proto-oncogene, GTPase
KREMEN1	kringle containing transmembrane protein 1
KRT5	keratin 5
KRT77	keratin 77
KSR2	kinase suppressor of ras 2
KXD1	KxDL motif containing 1
KYNU	kynureninase
LAMA1	laminin subunit alpha 1
LAMC1	laminin subunit gamma 1
LAMC2	laminin subunit gamma 2
LAPTM4A	lysosomal protein transmembrane 4 alpha
LARP4	La ribonucleoprotein domain family member 4
LARP4B	La ribonucleoprotein domain family member 4B
LASP1	LIM and SH3 protein 1
LBR	lamin B receptor
LCA5	LCA5, lebercilin
LCLAT1	lysocardiolipin acyltransferase 1
LCOR	ligand dependent nuclear receptor corepressor
LCORL	ligand dependent nuclear receptor corepressor like
LCP1	lymphocyte cytosolic protein 1
LCP2	lymphocyte cytosolic protein 2
LDHC	lactate dehydrogenase C
LEF1	lymphoid enhancer binding factor 1
LEMD2	LEM domain containing 2
LEPROTL1	leptin receptor overlapping transcript like 1
LETM2	leucine zipper and EF-hand containing transmembrane protein 2
LGI1	leucine rich glioma inactivated 1

LGR4	leucine rich repeat containing G protein-coupled receptor 4
LHFPL2	LHFPL tetraspan subfamily member 2
LHX6	LIM homeobox 6
LHX8	LIM homeobox 8
LHX9	LIM homeobox 9
LIFR	LIF receptor alpha
LILRA6	leukocyte immunoglobulin like receptor A6
LIMCH1	LIM and calponin homology domains 1
LIMD1	LIM domains containing 1
LIMD2	LIM domain containing 2
LIMS1	LIM zinc finger domain containing 1
LIN28A	lin-28 homolog A
LIN28B	lin-28 homolog B
LIN7C	lin-7 homolog C, crumbs cell polarity complex component
LINGO1	leucine rich repeat and Ig domain containing 1
LIPH	lipase H
LIPI	lipase I
LIX1	limb and CNS expressed 1
LMAN1	lectin, mannose binding 1
LMBR1	limb development membrane protein 1
LMBR1L	limb development membrane protein 1 like
LMBRD2	LMBR1 domain containing 2
LMLN	leishmanolysin like peptidase
LMO3	LIM domain only 3
LMO4	LIM domain only 4
LMO7	LIM domain 7
LMOD3	leiomodin 3
LMX1A	LIM homeobox transcription factor 1 alpha
LONRF1	LON peptidase N-terminal domain and ring finger 1
LONRF2	LON peptidase N-terminal domain and ring finger 2
LOR	loricrin
LOX	lysyl oxidase

LOXL2	lysyl oxidase like 2
LOXL3	lysyl oxidase like 3
LOXL4	lysyl oxidase like 4
LPAR1	lysophosphatidic acid receptor 1
LPCAT2	lysophosphatidylcholine acyltransferase 2
LPGAT1	lysophosphatidylglycerol acyltransferase 1
LRAT	lecithin retinol acyltransferase
LRCH1	leucine rich repeats and calponin homology domain containing 1
LRCH2	leucine rich repeats and calponin homology domain containing 2
LRFN2	leucine rich repeat and fibronectin type III domain containing 2
LRGUK	leucine rich repeats and guanylate kinase domain containing
LRIG1	leucine rich repeats and immunoglobulin like domains 1
LRIG2	leucine rich repeats and immunoglobulin like domains 2
LRP1B	LDL receptor related protein 1B
LRP4	LDL receptor related protein 4
LRP6	LDL receptor related protein 6
LRP8	LDL receptor related protein 8
LRR1	leucine rich repeat protein 1
LRRC17	leucine rich repeat containing 17
LRRC19	leucine rich repeat containing 19
LRRC2	leucine rich repeat containing 2
LRRC23	leucine rich repeat containing 23
LRRC40	leucine rich repeat containing 40
LRRC58	leucine rich repeat containing 58
LRRC59	leucine rich repeat containing 59
LRRC8A	leucine rich repeat containing 8 VRAC subunit A
LRRC8B	leucine rich repeat containing 8 VRAC subunit B
LRRC8C	leucine rich repeat containing 8 VRAC subunit C
LRRC8D	leucine rich repeat containing 8 VRAC subunit D
LRRFIP1	LRR binding FLII interacting protein 1
LRTM2	leucine rich repeats and transmembrane domains 2
LRTOMT	leucine rich transmembrane and O-methyltransferase domain containing

LSM11	LSM11, U7 small nuclear RNA associated
LSM12	LSM12 homolog
LSMEM1	leucine rich single-pass membrane protein 1
LTN1	listerin E3 ubiquitin protein ligase 1
LUC7L3	LUC7 like 3 pre-mRNA splicing factor
LYN	LYN proto-oncogene, Src family tyrosine kinase
LYPD6	LY6/PLAUR domain containing 6
LYRM2	LYR motif containing 2
LYRM7	LYR motif containing 7
LYSMD3	LysM domain containing 3
LYVE1	lymphatic vessel endothelial hyaluronan receptor 1
M1AP	meiosis 1 associated protein
MAB21L1	mab-21 like 1
MAB21L3	mab-21 like 3
MACF1	microtubule-actin crosslinking factor 1
MAEA	macrophage erythroblast attacher
MAFG	MAF bZIP transcription factor G
MAGEA3	MAGE family member A3
MAGEC1	MAGE family member C1
MAGI2	membrane associated guanylate kinase, WW and PDZ domain containing 2
MAGOHB	mago homolog B, exon junction complex subunit
MAGT1	magnesium transporter 1
MAMDC2	MAM domain containing 2
MAML1	mastermind like transcriptional coactivator 1
MAML2	mastermind like transcriptional coactivator 2
MAMLD1	mastermind like domain containing 1
MAN1A2	mannosidase alpha class 1A member 2
MAN1C1	mannosidase alpha class 1C member 1
MAN2A2	mannosidase alpha class 2A member 2
MAP10	microtubule associated protein 10
MAP1B	microtubule associated protein 1B
MAP2	microtubule associated protein 2

MAP3K1	mitogen-activated protein kinase kinase kinase 1
MAP3K12	mitogen-activated protein kinase kinase kinase 12
MAP3K13	mitogen-activated protein kinase kinase kinase 13
MAP3K2	mitogen-activated protein kinase kinase kinase 2
MAP3K5	mitogen-activated protein kinase kinase kinase 5
MAP3K9	mitogen-activated protein kinase kinase kinase 9
MAP4K2	mitogen-activated protein kinase kinase kinase kinase 2
MAP4K3	mitogen-activated protein kinase kinase kinase kinase 3
MAP4K4	mitogen-activated protein kinase kinase kinase kinase 4
MAP4K5	mitogen-activated protein kinase kinase kinase kinase 5
MAP7	microtubule associated protein 7
MAP9	microtubule associated protein 9
MAPK1	mitogen-activated protein kinase 1
MAPK1IP1L	mitogen-activated protein kinase 1 interacting protein 1 like
MAPK6	mitogen-activated protein kinase 6
MAPK8	mitogen-activated protein kinase 8
MAPK9	mitogen-activated protein kinase 9
MAPRE1	microtubule associated protein RP/EB family member 1
MAPRE3	microtubule associated protein RP/EB family member 3
MARK3	microtubule affinity regulating kinase 3
MARS2	methionyl-tRNA synthetase 2, mitochondrial
MASP1	mannan binding lectin serine peptidase 1
MAST3	microtubule associated serine/threonine kinase 3
MAT2A	methionine adenosyltransferase 2A
MATN1	matrilin 1
MATR3	matrin 3
MAU2	MAU2 sister chromatid cohesion factor
MB21D2	Mab-21 domain containing 2
MBD1	methyl-CpG binding domain protein 1
MBD2	methyl-CpG binding domain protein 2
MBD6	methyl-CpG binding domain protein 6
MBLAC2	metallo-beta-lactamase domain containing 2

MBNL1	muscleblind like splicing regulator 1
MBNL2	muscleblind like splicing regulator 2
MBNL3	muscleblind like splicing regulator 3
MBOAT2	membrane bound O-acyltransferase domain containing 2
MBTD1	mbt domain containing 1
MBTPS2	membrane bound transcription factor peptidase, site 2
MC2R	melanocortin 2 receptor
MCC	MCC, WNT signaling pathway regulator
MCF2L	MCF.2 cell line derived transforming sequence like
MCFD2	multiple coagulation factor deficiency 2
MCHR1	melanin concentrating hormone receptor 1
MCOLN2	mucolipin 2
MDM4	MDM4, p53 regulator
MECOM	MDS1 and EVI1 complex locus
MECP2	methyl-CpG binding protein 2
MED12L	mediator complex subunit 12 like
MED13L	mediator complex subunit 13 like
MED23	mediator complex subunit 23
MED28	mediator complex subunit 28
MED31	mediator complex subunit 31
MED6	mediator complex subunit 6
MED8	mediator complex subunit 8
MEF2C	myocyte enhancer factor 2C
MEF2D	myocyte enhancer factor 2D
MEIS2	Meis homeobox 2
MEOX2	mesenchyme homeobox 2
MET	MET proto-oncogene, receptor tyrosine kinase
METAP1	methionyl aminopeptidase 1
METAP2	methionyl aminopeptidase 2
METTL21A	methyltransferase like 21A
METTL9	methyltransferase like 9
MEX3A	mex-3 RNA binding family member A

MEX3B	mex-3 RNA binding family member B
MEX3C	mex-3 RNA binding family member C
MFAP3L	microfibril associated protein 3 like
MFAP5	microfibril associated protein 5
MFN2	mitofusin 2
MFSD11	major facilitator superfamily domain containing 11
MFSD12	major facilitator superfamily domain containing 12
MFSD8	major facilitator superfamily domain containing 8
MGA	MGA, MAX dimerization protein
MGAT2	mannosyl (alpha-1,6-)-glycoprotein beta-1,2-N-acetylglucosaminyltransferase
MGAT4A	alpha-1,3-mannosyl-glycoprotein 4-beta-N-acetylglucosaminyltransferase A
MIA3	MIA SH3 domain ER export factor 3
MIB1	mindbomb E3 ubiquitin protein ligase 1
MICAL1	microtubule associated monooxygenase, calponin and LIM domain containing 1
MICB	MHC class I polypeptide-related sequence B
MIER1	MIER1 transcriptional regulator
MIER2	MIER family member 2
MIER3	MIER family member 3
MINK1	misshapen like kinase 1
MINOS1	mitochondrial inner membrane organizing system 1
MINPP1	multiple inositol-polyphosphate phosphatase 1
MIOX	myo-inositol oxygenase
MIS12	MIS12, kinetochore complex component
MKL1	Megakaryoblastic Leukemia (Translocation) 1
MKL2	MKL1/Myocardin Like 2
MKLN1	Muskelin 1
MKNK2	MAP Kinase Interacting Serine/Threonine Kinase 2
MKRN3	makorin ring finger protein 3
MKX	mohawk homeobox
MLANA	melan-A
MLIP	muscular LMNA interacting protein
MLLT10	MLLT10, histone lysine methyltransferase DOT1L cofactor

MLXIP	MLX interacting protein
MMD	monocyte to macrophage differentiation associated
MMD2	monocyte to macrophage differentiation associated 2
MMP1	matrix metallopeptidase 1
MMP11	matrix metallopeptidase 11
MMP16	matrix metallopeptidase 16
MMP2	matrix metallopeptidase 2
MMP21	matrix metallopeptidase 21
MMP8	matrix metallopeptidase 8
MMRN1	multimerin 1
MMS22L	MMS22 like, DNA repair protein
MNT	MAX network transcriptional repressor
MNX1	motor neuron and pancreas homeobox 1
MOB3B	MOB kinase activator 3B
MOB4	MOB family member 4, phocein
MOG	myelin oligodendrocyte glycoprotein
MON2	MON2 homolog, regulator of endosome-to-Golgi trafficking
MORC2	MORC family CW-type zinc finger 2
MOXD1	monooxygenase DBH like 1
MPLKIP	M-phase specific PLK1 interacting protein
MPP5	membrane palmitoylated protein 5
MPP7	membrane palmitoylated protein 7
MPV17L	MPV17 mitochondrial inner membrane protein like
MPZ	myelin protein zero
MPZL2	myelin protein zero like 2
MRO	maestro
MRPL1	mitochondrial ribosomal protein L1
MRPS25	mitochondrial ribosomal protein S25
MRS2	magnesium transporter MRS2
MS4A1	membrane spanning 4-domains A1
MS4A7	membrane spanning 4-domains A7
MSANTD2	Myb/SANT DNA binding domain containing 2

MSANTD4	Myb/SANT DNA binding domain containing 4 with coiled-coils
MSI2	musashi RNA binding protein 2
MSL2	MSL complex subunit 2
MSMO1	methylsterol monooxygenase 1
MSN	moesin
MSR1	macrophage scavenger receptor 1
MSS51	MSS51 mitochondrial translational activator
MSTN	myostatin
MT1H	metallothionein 1H
MTAP	methylthioadenosine phosphorylase
MTBP	MDM2 binding protein
MTCP1	mature T cell proliferation 1
MTDH	metadherin
MTF2	metal response element binding transcription factor 2
MTHFD2	methylenetetrahydrofolate dehydrogenase (NADP+ dependent) 2, methenyltetrahydrofolate cyclohydrolase
MTM1	myotubularin 1
MTMR12	myotubularin related protein 12
MTMR3	myotubularin related protein 3
MTMR4	myotubularin related protein 4
MTMR9	myotubularin related protein 9
MTPN	myotrophin
MTR	5-methyltetrahydrofolate-homocysteine methyltransferase
MTSS1L	MTSS1L, I-BAR domain containing
MTTP	microsomal triglyceride transfer protein
MTUS1	microtubule associated scaffold protein 1
MTX2	metaxin 2
MTX3	metaxin 3
MUC1	mucin 1, cell surface associated
MUC17	mucin 17, cell surface associated
MUC20	mucin 20, cell surface associated
MXD1	MAX dimerization protein 1
MXD4	MAX dimerization protein 4

MXI1	MAX interactor 1, dimerization protein
MYB	MYB proto-oncogene, transcription factor
MYBL2	MYB proto-oncogene like 2
MYCBP	MYC binding protein
MYCBP2	MYC binding protein 2, E3 ubiquitin protein ligase
MYCN	MYCN proto-oncogene, bHLH transcription factor
MYH10	myosin heavy chain 10
MYO18A	myosin XVIIIA
MYO1F	myosin IF
MYO1H	myosin IH
MYO5B	myosin VB
MYO6	myosin VI
MYOCD	myocardin
MYOZ2	myozenin 2
MYRF	myelin regulatory factor
MYT1	myelin transcription factor 1
MZT1	mitotic spindle organizing protein 1
N4BP2	NEDD4 binding protein 2
NAA15	N(alpha)-acetyltransferase 15, NatA auxiliary subunit
NAA30	N(alpha)-acetyltransferase 30, NatC catalytic subunit
NAA50	N(alpha)-acetyltransferase 50, NatE catalytic subunit
NAALADL2	N-acetylated alpha-linked acidic dipeptidase like 2
NAB1	NGFI-A binding protein 1
NABP1	nucleic acid binding protein 1
NADK	NAD kinase
NAGPA	N-acetylglucosamine-1-phosphodiester alpha-N-acetylglucosaminidase
NAMPT	nicotinamide phosphoribosyltransferase
NANOS1	nanos C2HC-type zinc finger 1
NANOS2	nanos C2HC-type zinc finger 2
NANP	N-acetylneuraminic acid phosphatase
NAP1L1	nucleosome assembly protein 1 like 1
NAP1L2	nucleosome assembly protein 1 like 2

NAP1L5	nucleosome assembly protein 1 like 5
NAPG	NSF attachment protein gamma
NBEA	neurobeachin
NBR1	NBR1, autophagy cargo receptor
NCEH1	neutral cholesterol ester hydrolase 1
NCK2	NCK adaptor protein 2
NCKAP5	NCK associated protein 5
NCL	nucleolin
NCOA1	nuclear receptor coactivator 1
NCOA2	nuclear receptor coactivator 2
NCOA3	nuclear receptor coactivator 3
NCOA7	nuclear receptor coactivator 7
NCOR2	nuclear receptor corepressor 2
NCR3LG1	natural killer cell cytotoxicity receptor 3 ligand 1
NDEL1	nudE neurodevelopment protein 1 like 1
NDFIP2	Nedd4 family interacting protein 2
NDN	necdin, MAGE family member
NDRG2	NDRG family member 2
NDRG3	NDRG family member 3
NDRG4	NDRG family member 4
NDST2	N-deacetylase and N-sulfotransferase 2
NDST3	N-deacetylase and N-sulfotransferase 3
NDUFA4	NDUFA4, mitochondrial complex associated
NDUFA5	NADH:ubiquinone oxidoreductase subunit A5
NDUFC2	NADH:ubiquinone oxidoreductase subunit C2
NDUFS1	NADH:ubiquinone oxidoreductase core subunit S1
NECAB1	N-terminal EF-hand calcium binding protein 1
NECAP1	NECAP endocytosis associated 1
NEDD1	neural precursor cell expressed, developmentally down-regulated 1
NEDD4	neural precursor cell expressed, developmentally down-regulated 4, E3 ubiquitin protein ligase
NEFM	neurofilament medium
NEGR1	neuronal growth regulator 1

NEK1	NIMA related kinase 1
NEK10	NIMA related kinase 10
NEK7	NIMA related kinase 7
NEK9	NIMA related kinase 9
NEO1	neogenin 1
NET1	neuroepithelial cell transforming 1
NETO2	neuropilin and tolloid like 2
NEURL1B	neuralized E3 ubiquitin protein ligase 1B
NEURL4	neuralized E3 ubiquitin protein ligase 4
NEUROD1	neuronal differentiation 1
NEUROD4	neuronal differentiation 4
NEUROG1	neurogenin 1
NEUROG2	neurogenin 2
NFASC	neurofascin
NFAT5	nuclear factor of activated T cells 5
NFATC2	nuclear factor of activated T cells 2
NFATC3	nuclear factor of activated T cells 3
NFIA	nuclear factor I A
NFIB	nuclear factor I B
NFYA	nuclear transcription factor Y subunit alpha
NGF	nerve growth factor
NHLH2	nescient helix-loop-helix 2
NHLRC2	NHL repeat containing 2
NHLRC3	NHL repeat containing 3
NHS	NHS actin remodeling regulator
NICN1	nicolin 1
NINJ1	ninjurin 1
NIPA1	NIPA magnesium transporter 1
NIPAL2	NIPA like domain containing 2
NIPAL4	NIPA like domain containing 4
NKAIN2	sodium/potassium transporting ATPase interacting 2
NKD1	naked cuticle homolog 1

NKX2-2	NK2 homeobox 2
NKX3-2	NK3 homeobox 2
NLGN1	neuroligin 1
NLGN4X	neuroligin 4 X-linked
NLK	nemo like kinase
NLN	neurolysin
NLRP11	NLR family pyrin domain containing 11
NME4	NME/NM23 nucleoside diphosphate kinase 4
NME6	NME/NM23 nucleoside diphosphate kinase 6
NME9	NME/NM23 family member 9
NMNAT1	nicotinamide nucleotide adenylyltransferase 1
NMT1	N-myristoyltransferase 1
NMT2	N-myristoyltransferase 2
NNT	nicotinamide nucleotide transhydrogenase
NOG	noggin
NOTCH3	notch 3
NOVA1	NOVA alternative splicing regulator 1
NPAP1	nuclear pore associated protein 1
NPEPL1	aminopeptidase like 1
NPY1R	neuropeptide Y receptor Y1
NPY2R	neuropeptide Y receptor Y2
NR1D2	nuclear receptor subfamily 1 group D member 2
NR2F2	nuclear receptor subfamily 2 group F member 2
NR4A2	nuclear receptor subfamily 4 group A member 2
NR4A3	nuclear receptor subfamily 4 group A member 3
NR5A2	nuclear receptor subfamily 5 group A member 2
NR6A1	nuclear receptor subfamily 6 group A member 1
NRAS	NRAS proto-oncogene, GTPase
NRBF2	nuclear receptor binding factor 2
NRBP1	nuclear receptor binding protein 1
NRK	Nik related kinase
NRP1	neuropilin 1

NRXN1	neurexin 1
NRXN3	neurexin 3
NSD1	nuclear receptor binding SET domain protein 1
NSMAF	neutral sphingomyelinase activation associated factor
NSUN5	NOP2/Sun RNA methyltransferase family member 5
NT5C2	5'-nucleotidase, cytosolic II
NT5C3B	5'-nucleotidase, cytosolic IIIB
NT5DC1	5'-nucleotidase domain containing 1
NT5E	5'-nucleotidase ecto
NTF3	neurotrophin 3
NTN4	netrin 4
NTNG1	netrin G1
NTRK2	neurotrophic receptor tyrosine kinase 2
NTRK3	neurotrophic receptor tyrosine kinase 3
NUAK2	NUAK family kinase 2
NUCKS1	nuclear casein kinase and cyclin dependent kinase substrate 1
NUDT11	nudix hydrolase 11
NUDT4	nudix hydrolase 4
NUFIP2	nuclear FMR1 interacting protein 2
NUMBL	NUMB like, endocytic adaptor protein
NUP153	nucleoporin 153
NUP155	nucleoporin 155
NUS1	NUS1, dehydrodolichyl diphosphate synthase subunit
NWD1	NACHT and WD repeat domain containing 1
NXT2	nuclear transport factor 2 like export factor 2
NYNRIN	NYN domain and retroviral integrase containing
OARD1	O-acyl-ADP-ribose deacylase 1
OAS2	2'-5'-oligoadenylate synthetase 2
OAT	ornithine aminotransferase
OCLN	occludin
ODF2L	outer dense fiber of sperm tails 2 like
OGT	O-linked N-acetylglucosamine (GlcNAc) transferase

OLFM4	olfactomedin 4
OLFML2A	olfactomedin like 2A
OMG	oligodendrocyte myelin glycoprotein
ONECUT2	one cut homeobox 2
ONECUT3	one cut homeobox 3
OPCML	opioid binding protein/cell adhesion molecule like
OR11A1	olfactory receptor family 11 subfamily A member 1
OR7D2	olfactory receptor family 7 subfamily D member 2
ORC2	origin recognition complex subunit 2
ORC6	origin recognition complex subunit 6
ORMDL3	ORMDL sphingolipid biosynthesis regulator 3
OSBP	oxysterol binding protein
OSBPL11	oxysterol binding protein like 11
OSBPL2	oxysterol binding protein like 2
OSBPL3	oxysterol binding protein like 3
OSBPL5	oxysterol binding protein like 5
OSBPL7	oxysterol binding protein like 7
OSGIN2	oxidative stress induced growth inhibitor family member 2
OSMR	oncostatin M receptor
OSTF1	osteoclast stimulating factor 1
OSTM1	osteoclastogenesis associated transmembrane protein 1
OTOF	otoferlin
OTOGL	otogelin like
OTUB2	OTU deubiquitinase, ubiquitin aldehyde binding 2
OTUD1	OTU deubiquitinase 1
OTUD4	OTU deubiquitinase 4
OTUD5	OTU deubiquitinase 5
OTUD6B	OTU domain containing 6B
OTX2	orthodenticle homeobox 2
OVOL1	ovo like transcriptional repressor 1
OXCT1	3-oxoacid CoA-transferase 1
OXR1	oxidation resistance 1

P2RY10	P2Y receptor family member 10
P2RY13	purinergic receptor P2Y13
P4HA1	prolyl 4-hydroxylase subunit alpha 1
P4HA2	prolyl 4-hydroxylase subunit alpha 2
P4HA3	prolyl 4-hydroxylase subunit alpha 3
PAAF1	proteasomal ATPase associated factor 1
PABPC1L2A	poly(A) binding protein cytoplasmic 1 like 2A
PABPC1L2B	poly(A) binding protein cytoplasmic 1 like 2B
PAFAH1B2	platelet activating factor acetylhydrolase 1b catalytic subunit 2
PAFAH2	platelet activating factor acetylhydrolase 2
PAG1	phosphoprotein membrane anchor with glycosphingolipid microdomains 1
PAIP2	poly(A) binding protein interacting protein 2
PAK1	p21 (RAC1) activated kinase 1
PALD1	phosphatase domain containing, paladin 1
PALLD	palladin, cytoskeletal associated protein
PALM3	paralemmin 3
PAM	peptidylglycine alpha-amidating monooxygenase
PANK3	pantothenate kinase 3
PAPD4	
PAPD5	
PAPOLA	poly(A) polymerase alpha
PAPOLB	poly(A) polymerase beta
PAPPA	pappalysin 1
PAQR5	progestin and adipoQ receptor family member 5
PARD6B	par-6 family cell polarity regulator beta
PARM1	prostate androgen-regulated mucin-like protein 1
PARP11	poly(ADP-ribose) polymerase family member 11
PARP14	poly(ADP-ribose) polymerase family member 14
PARP16	poly(ADP-ribose) polymerase family member 16
PARP6	poly(ADP-ribose) polymerase family member 6
PARS2	prolyl-tRNA synthetase 2, mitochondrial
PARVB	parvin beta

PATE4	prostate and testis expressed 4
PATL1	PAT1 homolog 1, processing body mRNA decay factor
PAWR	pro-apoptotic WT1 regulator
PAX3	paired box 3
PAX7	paired box 7
PBRM1	polybromo 1
PBX1	PBX homeobox 1
PBX2	PBX homeobox 2
PBX3	PBX homeobox 3
PCBP4	poly(rC) binding protein 4
PCDH11Y	protocadherin 11 Y-linked
PCDH17	protocadherin 17
PCDH18	protocadherin 18
PCDH19	protocadherin 19
PCDH20	protocadherin 20
PCDH8	protocadherin 8
PCDHA1	protocadherin alpha 1
PCDHA10	protocadherin alpha 10
PCDHA11	protocadherin alpha 11
PCDHA12	protocadherin alpha 12
PCDHA13	protocadherin alpha 13
PCDHA2	protocadherin alpha 2
PCDHA3	protocadherin alpha 3
PCDHA4	protocadherin alpha 4
PCDHA5	protocadherin alpha 5
PCDHA6	protocadherin alpha 6
PCDHA7	protocadherin alpha 7
PCDHA8	protocadherin alpha 8
PCDHA9	protocadherin alpha 9
PCDHAC1	protocadherin alpha subfamily C, 1
PCDHAC2	protocadherin alpha subfamily C, 2
PCGF3	polycomb group ring finger 3

PCGF5	polycomb group ring finger 5
PCMTD1	protein-L-isoaspartate (D-aspartate) O-methyltransferase domain containing 1
PCMTD2	protein-L-isoaspartate (D-aspartate) O-methyltransferase domain containing 2
PCNA	proliferating cell nuclear antigen
PCNP	PEST proteolytic signal containing nuclear protein
PCSK2	proprotein convertase subtilisin/kexin type 2
PCTP	phosphatidylcholine transfer protein
PCYOX1L	prenylcysteine oxidase 1 like
PDCD10	programmed cell death 10
PDCD2L	programmed cell death 2 like
PDCD4	programmed cell death 4
PDE12	phosphodiesterase 12
PDE2A	phosphodiesterase 2A
PDE4B	phosphodiesterase 4B
PDE4D	phosphodiesterase 4D
PDE5A	phosphodiesterase 5A
PDE7A	phosphodiesterase 7A
PDGFA	platelet derived growth factor subunit A
PDGFRA	platelet derived growth factor receptor alpha
PDHX	pyruvate dehydrogenase complex component X
PDIA3	protein disulfide isomerase family A member 3
PDIK1L	PDLIM1 interacting kinase 1 like
PDK4	pyruvate dehydrogenase kinase 4
PDP2	pyruvate dehyrogenase phosphatase catalytic subunit 2
PDPR	pyruvate dehydrogenase phosphatase regulatory subunit
PDXK	pyridoxal kinase
PDZD9	PDZ domain containing 9
PEG10	paternally expressed 10
PELO	pelota mRNA surveillance and ribosome rescue factor
PEX11B	peroxisomal biogenesis factor 11 beta
PEX5L	peroxisomal biogenesis factor 5 like
PFAS	phosphoribosylformylglycinamidine synthase

PFDN1	prefoldin subunit 1
PFDN4	prefoldin subunit 4
PFKFB3	6-phosphofructo-2-kinase/fructose-2,6-biphosphatase 3
PFN2	profilin 2
PFN4	profilin family member 4
PGAP1	post-GPI attachment to proteins 1
PGAP2	post-GPI attachment to proteins 2
PGBD2	piggyBac transposable element derived 2
PGBD5	piggyBac transposable element derived 5
PGD	phosphogluconate dehydrogenase
PGM1	phosphoglucomutase 1
PGM2	phosphoglucomutase 2
PGM2L1	phosphoglucomutase 2 like 1
PGM3	phosphoglucomutase 3
PGP	phosphoglycolate phosphatase
PGPEP1	pyroglutamyl-peptidase I
PGRMC1	progesterone receptor membrane component 1
PHACTR2	phosphatase and actin regulator 2
PHACTR3	phosphatase and actin regulator 3
PHAX	phosphorylated adaptor for RNA export
PHC2	polyhomeotic homolog 2
PHC3	polyhomeotic homolog 3
PHF21B	PHD finger protein 21B
PHF6	PHD finger protein 6
PHIP	pleckstrin homology domain interacting protein
PHLDB2	pleckstrin homology like domain family B member 2
PHLPP1	PH domain and leucine rich repeat protein phosphatase 1
PHLPP2	PH domain and leucine rich repeat protein phosphatase 2
PHOX2A	paired like homeobox 2A
PHTF2	putative homeodomain transcription factor 2
PI15	peptidase inhibitor 15
PI4K2B	phosphatidylinositol 4-kinase type 2 beta

PICALM	phosphatidylinositol binding clathrin assembly protein
PIEZO2	piezo type mechanosensitive ion channel component 2
PIGA	phosphatidylinositol glycan anchor biosynthesis class A
PIGF	phosphatidylinositol glycan anchor biosynthesis class F
PIGM	phosphatidylinositol glycan anchor biosynthesis class M
PIGX	phosphatidylinositol glycan anchor biosynthesis class X
PIH1D3	PIH1 domain containing 3
PIK3C2A	phosphatidylinositol-4-phosphate 3-kinase catalytic subunit type 2 alpha
PIK3C2B	phosphatidylinositol-4-phosphate 3-kinase catalytic subunit type 2 beta
PIK3CA	phosphatidylinositol-4,5-bisphosphate 3-kinase catalytic subunit alpha
PIK3IP1	phosphoinositide-3-kinase interacting protein 1
PIM1	Pim-1 proto-oncogene, serine/threonine kinase
PIM2	Pim-2 proto-oncogene, serine/threonine kinase
PIN1	peptidylprolyl cis/trans isomerase, NIMA-interacting 1
PIP4K2A	phosphatidylinositol-5-phosphate 4-kinase type 2 alpha
PIRT	phosphoinositide interacting regulator of transient receptor potential channels
PITPNA	phosphatidylinositol transfer protein alpha
PITPNB	phosphatidylinositol transfer protein beta
PITPNC1	phosphatidylinositol transfer protein cytoplasmic 1
PITPNM2	phosphatidylinositol transfer protein membrane associated 2
PKD1	polycystin 1, transient receptor potential channel interacting
PKD2	polycystin 2, transient receptor potential cation channel
PKHD1	PKHD1, fibrocystin/polyductin
PKIA	cAMP-dependent protein kinase inhibitor alpha
PKN2	protein kinase N2
PLA2G12A	phospholipase A2 group XIIA
PLA2G2C	phospholipase A2 group IIC
PLA2G2F	phospholipase A2 group IIF
PLA2G3	phospholipase A2 group III
PLA2G4E	phospholipase A2 group IVE
PLAG1	PLAG1 zinc finger
PLAGL2	PLAG1 like zinc finger 2

PLBD2	phospholipase B domain containing 2
PLCB4	phospholipase C beta 4
PLCD4	phospholipase C delta 4
PLCG1	phospholipase C gamma 1
PLCL1	phospholipase C like 1 (inactive)
PLCXD3	phosphatidylinositol specific phospholipase C X domain containing 3
PLD5	phospholipase D family member 5
PLEKHA3	pleckstrin homology domain containing A3
PLEKHA7	pleckstrin homology domain containing A7
PLEKHA8	pleckstrin homology domain containing A8
PLEKHB2	pleckstrin homology domain containing B2
PLEKHG2	pleckstrin homology and RhoGEF domain containing G2
PLEKHG6	pleckstrin homology and RhoGEF domain containing G6
PLEKHG7	pleckstrin homology and RhoGEF domain containing G7
PLEKHH1	pleckstrin homology, MyTH4 and FERM domain containing H1
PLEKHO1	pleckstrin homology domain containing O1
PLEKHO2	pleckstrin homology domain containing O2
PLGLB1	plasminogen-like B1
PLGLB2	plasminogen-like B2
PLGRKT	plasminogen receptor with a C-terminal lysine
PLLP	plasmolipin
PLS3	plastin 3
PLXNA1	plexin A1
PLXNA2	plexin A2
PLXNA4	plexin A4
PLXNC1	plexin C1
PM20D2	peptidase M20 domain containing 2
PMAIP1	phorbol-12-myristate-13-acetate-induced protein 1
PMM1	phosphomannomutase 1
PMP2	peripheral myelin protein 2
PNKD	PNKD, MBL domain containing
PNMA1	PNMA family member 1

PNO1	partner of NOB1 homolog
PNP	purine nucleoside phosphorylase
PNPLA1	patatin like phospholipase domain containing 1
PNRC2	proline rich nuclear receptor coactivator 2
POC1B-GALNT4	POC1B-GALNT4 readthrough
POF1B	POF1B, actin binding protein
POGK	pogo transposable element derived with KRAB domain
POGZ	pogo transposable element derived with ZNF domain
POLD3	DNA polymerase delta 3, accessory subunit
POLE	DNA polymerase epsilon, catalytic subunit
POLE3	DNA polymerase epsilon 3, accessory subunit
POLQ	DNA polymerase theta
POLR1D	RNA polymerase I and III subunit D
POLR2D	RNA polymerase II subunit D
POLR2G	RNA polymerase II subunit G
POLR2J3	RNA polymerase II subunit J3
POLR3G	RNA polymerase III subunit G
PON2	paraoxonase 2
POP1	POP1 homolog, ribonuclease P/MRP subunit
PORCN	porcupine O-acyltransferase
POTEE	POTE ankyrin domain family member E
POU2F1	POU class 2 homeobox 1
POU2F2	POU class 2 homeobox 2
POU6F1	POU class 6 homeobox 1
POU6F2	POU class 6 homeobox 2
PPARA	peroxisome proliferator activated receptor alpha
PPARG	peroxisome proliferator activated receptor gamma
PPARGC1A	PPARG coactivator 1 alpha
PPARGC1B	PPARG coactivator 1 beta
PPFIA1	PTPRF interacting protein alpha 1
PPFIBP1	PPFIA binding protein 1
PPHLN1	periphilin 1

PPIB	peptidylprolyl isomerase B
PPIL2	peptidylprolyl isomerase like 2
PPIL3	peptidylprolyl isomerase like 3
PPIL4	peptidylprolyl isomerase like 4
PPM1F	protein phosphatase, Mg2+/Mn2+ dependent 1F
PPP1CB	protein phosphatase 1 catalytic subunit beta
PPP1R12B	protein phosphatase 1 regulatory subunit 12B
PPP1R14C	protein phosphatase 1 regulatory inhibitor subunit 14C
PPP1R16B	protein phosphatase 1 regulatory subunit 16B
PPP1R18	protein phosphatase 1 regulatory subunit 18
PPP2CA	protein phosphatase 2 catalytic subunit alpha
PPP2R2A	protein phosphatase 2 regulatory subunit Balpha
PPP2R2B	protein phosphatase 2 regulatory subunit Bbeta
PPP2R3C	protein phosphatase 2 regulatory subunit B''gamma
PPP3CA	protein phosphatase 3 catalytic subunit alpha
PPP3CB	protein phosphatase 3 catalytic subunit beta
PPP3R1	protein phosphatase 3 regulatory subunit B, alpha
PPP3R2	protein phosphatase 3 regulatory subunit B, beta
PPP4C	protein phosphatase 4 catalytic subunit
PPP4R1	protein phosphatase 4 regulatory subunit 1
PPP4R2	protein phosphatase 4 regulatory subunit 2
PPP6C	protein phosphatase 6 catalytic subunit
PPP6R1	protein phosphatase 6 regulatory subunit 1
PPP6R2	protein phosphatase 6 regulatory subunit 2
PPP6R3	protein phosphatase 6 regulatory subunit 3
PPT1	palmitoyl-protein thioesterase 1
PPT2	palmitoyl-protein thioesterase 2
PPTC7	PTC7 protein phosphatase homolog
PPWD1	peptidylprolyl isomerase domain and WD repeat containing 1
PQLC2	PQ loop repeat containing 2
PRDM1	PR/SET domain 1
PRDM12	PR/SET domain 12

PRDM13	PR/SET domain 13
PRDM16	PR/SET domain 16
PRDM2	PR/SET domain 2
PRDM5	PR/SET domain 5
PREB	prolactin regulatory element binding
PRG4	proteoglycan 4
PRKAA1	protein kinase AMP-activated catalytic subunit alpha 1
PRKAA2	protein kinase AMP-activated catalytic subunit alpha 2
PRKAB2	protein kinase AMP-activated non-catalytic subunit beta 2
PRKACB	protein kinase cAMP-activated catalytic subunit beta
PRKAR2A	protein kinase cAMP-dependent type II regulatory subunit alpha
PRKAR2B	protein kinase cAMP-dependent type II regulatory subunit beta
PRKCD	protein kinase C delta
PRKCE	protein kinase C epsilon
PRKCQ	protein kinase C theta
PRMT3	protein arginine methyltransferase 3
PROK2	prokineticin 2
PROP1	PROP paired-like homeobox 1
PRPF38B	pre-mRNA processing factor 38B
PRPF40A	pre-mRNA processing factor 40 homolog A
PRR15	proline rich 15
PRR18	proline rich 18
PRR22	proline rich 22
PRR23A	proline rich 23A
PRR9	proline rich 9
PRRC2C	proline rich coiled-coil 2C
PRRG1	proline rich and Gla domain 1
PRRG4	proline rich and Gla domain 4
PRRT2	proline rich transmembrane protein 2
PRRX1	paired related homeobox 1
PRTG	protogenin
PSAP	prosaposin

PSAT1	phosphoserine aminotransferase 1
PSD3	pleckstrin and Sec7 domain containing 3
PSIP1	PC4 and SFRS1 interacting protein 1
PSKH1	protein serine kinase H1
PSME4	proteasome activator subunit 4
PSPH	phosphoserine phosphatase
PSTK	phosphoseryl-tRNA kinase
PSTPIP2	proline-serine-threonine phosphatase interacting protein 2
PTAFR	platelet activating factor receptor
PTAR1	protein prenyltransferase alpha subunit repeat containing 1
PTBP1	polypyrimidine tract binding protein 1
PTBP2	polypyrimidine tract binding protein 2
PTCH1	patched 1
PTCHD4	patched domain containing 4
PTDSS1	phosphatidylserine synthase 1
PTEN	phosphatase and tensin homolog
PTGER3	prostaglandin E receptor 3
PTGER4	prostaglandin E receptor 4
PTGFRN	prostaglandin F2 receptor inhibitor
PTGS2	prostaglandin-endoperoxide synthase 2
PTMA	prothymosin alpha
PTP4A1	protein tyrosine phosphatase type IVA, member 1
PTPDC1	protein tyrosine phosphatase domain containing 1
PTPN12	protein tyrosine phosphatase, non-receptor type 12
PTPN13	protein tyrosine phosphatase, non-receptor type 13
PTPN18	protein tyrosine phosphatase, non-receptor type 18
PTPN21	protein tyrosine phosphatase, non-receptor type 21
PTPN3	protein tyrosine phosphatase, non-receptor type 3
PTPN4	protein tyrosine phosphatase, non-receptor type 4
PTPRD	protein tyrosine phosphatase, receptor type D
PTPRG	protein tyrosine phosphatase, receptor type G
PTPRK	protein tyrosine phosphatase, receptor type K

PTPRO	protein tyrosine phosphatase, receptor type O
PTPRT	protein tyrosine phosphatase, receptor type T
PTPRZ1	protein tyrosine phosphatase, receptor type Z1
PUM2	pumilio RNA binding family member 2
PURA	purine rich element binding protein A
PURB	purine rich element binding protein B
PURG	purine rich element binding protein G
PUS7L	pseudouridine synthase 7 like
PXDN	peroxidasin
PXT1	peroxisomal testis enriched protein 1
PYCR1	pyrroline-5-carboxylate reductase 1
PYGO2	pygopus family PHD finger 2
QARS	glutaminyl-tRNA synthetase
QKI	QKI, KH domain containing RNA binding
R3HDM1	R3H domain containing 1
RAB11FIP1	RAB11 family interacting protein 1
RAB11FIP2	RAB11 family interacting protein 2
RAB11FIP5	RAB11 family interacting protein 5
RAB14	RAB14, member RAS oncogene family
RAB15	RAB15, member RAS oncogene family
RAB18	RAB18, member RAS oncogene family
RAB21	RAB21, member RAS oncogene family
RAB22A	RAB22A, member RAS oncogene family
RAB23	RAB23, member RAS oncogene family
RAB27B	RAB27B, member RAS oncogene family
RAB2B	RAB2B, member RAS oncogene family
RAB31	RAB31, member RAS oncogene family
RAB32	RAB32, member RAS oncogene family
RAB33B	RAB33B, member RAS oncogene family
RAB35	RAB35, member RAS oncogene family
RAB37	RAB37, member RAS oncogene family
RAB38	RAB38, member RAS oncogene family

RAB3D	RAB3D, member RAS oncogene family
RAB3GAP2	RAB3 GTPase activating non-catalytic protein subunit 2
RAB3IP	RAB3A interacting protein
RAB43	RAB43, member RAS oncogene family
RAB5A	RAB5A, member RAS oncogene family
RAB7A	RAB7A, member RAS oncogene family
RAB8A	RAB8A, member RAS oncogene family
RAB8B	RAB8B, member RAS oncogene family
RABEPK	Rab9 effector protein with kelch motifs
RABGAP1L	RAB GTPase activating protein 1 like
RAD23B	RAD23 homolog B, nucleotide excision repair protein
RAD54B	RAD54 homolog B
RAD9A	RAD9 checkpoint clamp component A
RAG1	recombination activating 1
RALB	RAS like proto-oncogene B
RALGDS	ral guanine nucleotide dissociation stimulator
RALGPS1	Ral GEF with PH domain and SH3 binding motif 1
RALYL	RALY RNA binding protein like
RANBP10	RAN binding protein 10
RANBP17	RAN binding protein 17
RANBP2	RAN binding protein 2
RANBP9	RAN binding protein 9
RANGRF	RAN guanine nucleotide release factor
RAP1A	RAP1A, member of RAS oncogene family
RAP1B	RAP1B, member of RAS oncogene family
RAP2B	RAP2B, member of RAS oncogene family
RAP2C	RAP2C, member of RAS oncogene family
RAPGEF2	Rap guanine nucleotide exchange factor 2
RAPGEF4	Rap guanine nucleotide exchange factor 4
RAPH1	Ras association (RalGDS/AF-6) and pleckstrin homology domains 1
RARB	retinoic acid receptor beta
RARG	retinoic acid receptor gamma

RARRES1	retinoic acid receptor responder 1
RASA1	RAS p21 protein activator 1
RASGRF2	Ras protein specific guanine nucleotide releasing factor 2
RASGRP1	RAS guanyl releasing protein 1
RASL11B	RAS like family 11 member B
RASSF2	Ras association domain family member 2
RASSF6	Ras association domain family member 6
RASSF8	Ras association domain family member 8
RAVER2	ribonucleoprotein, PTB binding 2
RB1	RB transcriptional corepressor 1
RBBP9	RB binding protein 9, serine hydrolase
RBFOX1	RNA binding fox-1 homolog 1
RBFOX2	RNA binding fox-1 homolog 2
RBFOX3	RNA binding fox-1 homolog 3
RBM12	RNA binding motif protein 12
RBM12B	RNA binding motif protein 12B
RBM24	RNA binding motif protein 24
RBM38	RNA binding motif protein 38
RBM43	RNA binding motif protein 43
RBM46	RNA binding motif protein 46
RBM47	RNA binding motif protein 47
RBM48	RNA binding motif protein 48
RBMS1	RNA binding motif single stranded interacting protein 1
RBMXL2	RBMX like 2
RC3H1	ring finger and CCCH-type domains 1
RCAN2	regulator of calcineurin 2
RCBTB1	RCC1 and BTB domain containing protein 1
RCN1	reticulocalbin 1
RCN2	reticulocalbin 2
RDH10	retinol dehydrogenase 10
RDH14	retinol dehydrogenase 14
RDX	radixin

RECK	reversion inducing cysteine rich protein with kazal motifs
REEP1	receptor accessory protein 1
REEP3	receptor accessory protein 3
REEP4	receptor accessory protein 4
RELN	reelin
REPIN1	replication initiator 1
REPS2	RALBP1 associated Eps domain containing 2
REST	RE1 silencing transcription factor
REV1	REV1, DNA directed polymerase
REV3L	REV3 like, DNA directed polymerase zeta catalytic subunit
RFC3	replication factor C subunit 3
RFFL	ring finger and FYVE like domain containing E3 ubiquitin protein ligase
RFTN2	raftlin family member 2
RFWD3	ring finger and WD repeat domain 3
RFX2	regulatory factor X2
RFX6	regulatory factor X6
RGL1	ral guanine nucleotide dissociation stimulator like 1
RGMB	repulsive guidance molecule BMP co-receptor b
RGS16	regulator of G protein signaling 16
RGS22	regulator of G protein signaling 22
RGS4	regulator of G protein signaling 4
RGS7	regulator of G protein signaling 7
RGS7BP	regulator of G protein signaling 7 binding protein
RHD	Rh blood group D antigen
RHEBL1	RHEB like 1
RHOB	ras homolog family member B
RHOQ	ras homolog family member Q
RHOT1	ras homolog family member T1
RHOU	ras homolog family member U
RHPN2	rhophilin Rho GTPase binding protein 2
RICTOR	RPTOR independent companion of MTOR complex 2
RIMBP2	RIMS binding protein 2

RIMKLA	ribosomal modification protein rimK like family member A
RIMKLB	ribosomal modification protein rimK like family member B
RIMS1	regulating synaptic membrane exocytosis 1
RIMS2	regulating synaptic membrane exocytosis 2
RIMS3	regulating synaptic membrane exocytosis 3
RIOK3	RIO kinase 3
RIT2	Ras like without CAAX 2
RLIM	ring finger protein, LIM domain interacting
RMDN2	regulator of microtubule dynamics 2
RMDN3	regulator of microtubule dynamics 3
RMI2	RecQ mediated genome instability 2
RMND5A	required for meiotic nuclear division 5 homolog A
RNASEH2C	ribonuclease H2 subunit C
RND3	Rho family GTPase 3
RNF111	ring finger protein 111
RNF122	ring finger protein 122
RNF125	ring finger protein 125
RNF138	ring finger protein 138
RNF139	ring finger protein 139
RNF141	ring finger protein 141
RNF145	ring finger protein 145
RNF157	ring finger protein 157
RNF165	ring finger protein 165
RNF170	ring finger protein 170
RNF185	ring finger protein 185
RNF186	ring finger protein 186
RNF2	ring finger protein 2
RNF20	ring finger protein 20
RNF213	ring finger protein 213
RNF217	ring finger protein 217
RNF220	ring finger protein 220
RNF24	ring finger protein 24

RNF31	ring finger protein 31
RNF38	ring finger protein 38
RNF44	ring finger protein 44
RNF6	ring finger protein 6
RNF8	ring finger protein 8
RNFT1	ring finger protein, transmembrane 1
RNGTT	RNA guanylyltransferase and 5'-phosphatase
RNMT	RNA guanine-7 methyltransferase
ROBO1	roundabout guidance receptor 1
ROCK1	Rho associated coiled-coil containing protein kinase 1
ROGDI	rogdi homolog
ROR1	receptor tyrosine kinase like orphan receptor 1
RORA	RAR related orphan receptor A
RORB	RAR related orphan receptor B
RORC	RAR related orphan receptor C
RP2	RP2, ARL3 GTPase activating protein
RPA2	replication protein A2
RPAP2	RNA polymerase II associated protein 2
RPE	ribulose-5-phosphate-3-epimerase
RPL10	ribosomal protein L10
RPL22L1	ribosomal protein L22 like 1
RPL23	ribosomal protein L23
RPL26	ribosomal protein L26
RPL37	ribosomal protein L37
RPP25	ribonuclease P and MRP subunit p25
RPRD1A	regulation of nuclear pre-mRNA domain containing 1A
RPRD2	regulation of nuclear pre-mRNA domain containing 2
RPS15A	ribosomal protein S15a
RPS6KA1	ribosomal protein S6 kinase A1
RPS6KA2	ribosomal protein S6 kinase A2
RPS6KA3	ribosomal protein S6 kinase A3
RPS6KA6	ribosomal protein S6 kinase A6

RPS6KB1	ribosomal protein S6 kinase B1
RPS6KB2	ribosomal protein S6 kinase B2
RRAD	RRAD, Ras related glycolysis inhibitor and calcium channel regulator
RRAGD	Ras related GTP binding D
RRBP1	ribosome binding protein 1
RRM2	ribonucleotide reductase regulatory subunit M2
RRP15	ribosomal RNA processing 15 homolog
RRP1B	ribosomal RNA processing 1B
RSBN1	round spermatid basic protein 1
RSBN1L	round spermatid basic protein 1 like
RSPO3	R-spondin 3
RSPRY1	ring finger and SPRY domain containing 1
RTKN2	rhotekin 2
RTN1	reticulon 1
RTN4R	reticulon 4 receptor
RTTN	rotatin
RUFY2	RUN and FYVE domain containing 2
RUFY3	RUN and FYVE domain containing 3
RUNDC3B	RUN domain containing 3B
RUNX1T1	RUNX1 translocation partner 1
RUNX2	runt related transcription factor 2
RUSC2	RUN and SH3 domain containing 2
RWDD2A	RWD domain containing 2A
RWDD4	RWD domain containing 4
S100A7A	S100 calcium binding protein A7A
S100A8	S100 calcium binding protein A8
S100PBP	S100P binding protein
SACM1L	SAC1 like phosphatidylinositide phosphatase
SACS	sacsin molecular chaperone
SAMD12	sterile alpha motif domain containing 12
SAMSN1	SAM domain, SH3 domain and nuclear localization signals 1
SAP130	Sin3A associated protein 130

SAR1B	secretion associated Ras related GTPase 1B
SARNP	SAP domain containing ribonucleoprotein
SASS6	SAS-6 centriolar assembly protein
SBK1	SH3 domain binding kinase 1
SBNO1	strawberry notch homolog 1
SCAF4	SR-related CTD associated factor 4
SCARA5	scavenger receptor class A member 5
SCARF1	scavenger receptor class F member 1
SCD	stearoyl-CoA desaturase
SCHIP1	schwannomin interacting protein 1
SCML1	Scm polycomb group protein like 1
SCML2	Scm polycomb group protein like 2
SCML4	Scm polycomb group protein like 4
SCN1A	sodium voltage-gated channel alpha subunit 1
SCN2A	sodium voltage-gated channel alpha subunit 2
SCN3A	sodium voltage-gated channel alpha subunit 3
SCN3B	sodium voltage-gated channel beta subunit 3
SCN5A	sodium voltage-gated channel alpha subunit 5
SCN8A	sodium voltage-gated channel alpha subunit 8
SCN9A	sodium voltage-gated channel alpha subunit 9
SCOC	short coiled-coil protein
SCRT2	scratch family transcriptional repressor 2
SCYL2	SCY1 like pseudokinase 2
SCYL3	SCY1 like pseudokinase 3
SDAD1	SDA1 domain containing 1
SDR42E1	short chain dehydrogenase/reductase family 42E, member 1
SEC22A	SEC22 homolog A, vesicle trafficking protein
SEC22C	SEC22 homolog C, vesicle trafficking protein
SEC23A	Sec23 homolog A, coat complex II component
SEC23IP	SEC23 interacting protein
SEC24C	SEC24 homolog C, COPII coat complex component
SEC31A	SEC31 homolog A, COPII coat complex component

SEC61A2	Sec61 translocon alpha 2 subunit
SEC62	SEC62 homolog, preprotein translocation factor
SEC63	SEC63 homolog, protein translocation regulator
SECISBP2L	SECIS binding protein 2 like
SELP	selectin P
SEMA3A	semaphorin 3A
SEMA3F	semaphorin 3F
SEMA4B	semaphorin 4B
SEMA4C	semaphorin 4C
SEMA4G	semaphorin 4G
SEMA5A	semaphorin 5A
SEMA6A	semaphorin 6A
SEMA6D	semaphorin 6D
SENP2	SUMO specific peptidase 2
SENP5	SUMO specific peptidase 5
SENP7	SUMO specific peptidase 7
SERBP1	SERPINE1 mRNA binding protein 1
SERINC1	serine incorporator 1
SERINC3	serine incorporator 3
SERP1	stress associated endoplasmic reticulum protein 1
SERPINA10	serpin family A member 10
SERPINB9	serpin family B member 9
SERPINI1	serpin family I member 1
SERTAD2	SERTA domain containing 2
SESN1	sestrin 1
SESTD1	SEC14 and spectrin domain containing 1
SETD3	SET domain containing 3
SETD7	SET domain containing lysine methyltransferase 7
SETDB1	SET domain bifurcated 1
SF3A1	splicing factor 3a subunit 1
SFMBT1	Scm like with four mbt domains 1
SFPQ	splicing factor proline and glutamine rich

SFR1	SWI5 dependent homologous recombination repair protein 1
SFSWAP	splicing factor SWAP
SFT2D2	SFT2 domain containing 2
SFTA3	surfactant associated 3
SFXN1	sideroflexin 1
SGCB	sarcoglycan beta
SGMS2	sphingomyelin synthase 2
SGPL1	sphingosine-1-phosphate lyase 1
SGPP1	sphingosine-1-phosphate phosphatase 1
SGTB	small glutamine rich tetratricopeptide repeat containing beta
SH2B3	SH2B adaptor protein 3
SH2D4A	SH2 domain containing 4A
SH2D5	SH2 domain containing 5
SH3BGRL3	SH3 domain binding glutamate rich protein like 3
SH3GL1	SH3 domain containing GRB2 like 1, endophilin A2
SH3GLB1	SH3 domain containing GRB2 like, endophilin B1
SH3PXD2A	SH3 and PX domains 2A
SH3PXD2B	SH3 and PX domains 2B
SH3RF1	SH3 domain containing ring finger 1
SH3TC1	SH3 domain and tetratricopeptide repeats 1
SH3TC2	SH3 domain and tetratricopeptide repeats 2
SH3YL1	SH3 and SYLF domain containing 1
SHANK2	SH3 and multiple ankyrin repeat domains 2
SHB	SH2 domain containing adaptor protein B
SHC4	SHC adaptor protein 4
SHISA2	shisa family member 2
SHISA3	shisa family member 3
SHOC2	SHOC2, leucine rich repeat scaffold protein
SIGLEC14	sialic acid binding Ig like lectin 14
SIKE1	suppressor of IKBKE 1
SIM1	SIM bHLH transcription factor 1
SIRT1	sirtuin 1

SIX1	SIX homeobox 1
SIX3	SIX homeobox 3
SIX4	SIX homeobox 4
SKA2	spindle and kinetochore associated complex subunit 2
SKA3	spindle and kinetochore associated complex subunit 3
SKIDA1	SKI/DACH domain containing 1
SKIL	SKI like proto-oncogene
SKP2	S-phase kinase associated protein 2
SLA2	Src like adaptor 2
SLAIN2	SLAIN motif family member 2
SLAMF6	SLAM family member 6
SLC10A7	solute carrier family 10 member 7
SLC11A1	solute carrier family 11 member 1
SLC12A2	solute carrier family 12 member 2
SLC12A5	solute carrier family 12 member 5
SLC12A9	solute carrier family 12 member 9
SLC13A1	solute carrier family 13 member 1
SLC14A1	solute carrier family 14 member 1 (Kidd blood group)
SLC15A5	solute carrier family 15 member 5
SLC16A10	solute carrier family 16 member 10
SLC16A14	solute carrier family 16 member 14
SLC16A2	solute carrier family 16 member 2
SLC16A6	solute carrier family 16 member 6
SLC16A9	solute carrier family 16 member 9
SLC17A7	solute carrier family 17 member 7
SLC17A9	solute carrier family 17 member 9
SLC19A2	solute carrier family 19 member 2
SLC19A3	solute carrier family 19 member 3
SLC1A2	solute carrier family 1 member 2
SLC20A1	solute carrier family 20 member 1
SLC22A14	solute carrier family 22 member 14
SLC22A23	solute carrier family 22 member 23

SLC22A5	solute carrier family 22 member 5
SLC24A1	solute carrier family 24 member 1
SLC24A2	solute carrier family 24 member 2
SLC24A4	solute carrier family 24 member 4
SLC25A16	solute carrier family 25 member 16
SLC25A18	solute carrier family 25 member 18
SLC25A20	solute carrier family 25 member 20
SLC25A22	solute carrier family 25 member 22
SLC25A24	solute carrier family 25 member 24
SLC25A25	solute carrier family 25 member 25
SLC25A27	solute carrier family 25 member 27
SLC25A30	solute carrier family 25 member 30
SLC25A32	solute carrier family 25 member 32
SLC25A4	solute carrier family 25 member 4
SLC25A40	solute carrier family 25 member 40
SLC25A45	solute carrier family 25 member 45
SLC26A2	solute carrier family 26 member 2
SLC26A4	solute carrier family 26 member 4
SLC28A1	solute carrier family 28 member 1
SLC28A3	solute carrier family 28 member 3
SLC29A3	solute carrier family 29 member 3
SLC2A12	solute carrier family 2 member 12
SLC30A1	solute carrier family 30 member 1
SLC30A4	solute carrier family 30 member 4
SLC30A5	solute carrier family 30 member 5
SLC30A6	solute carrier family 30 member 6
SLC30A7	solute carrier family 30 member 7
SLC31A1	solute carrier family 31 member 1
SLC31A2	solute carrier family 31 member 2
SLC35A3	solute carrier family 35 member A3
SLC35A5	solute carrier family 35 member A5
SLC35B1	solute carrier family 35 member B1

SLC35B2	solute carrier family 35 member B2
SLC35B4	solute carrier family 35 member B4
SLC35C1	solute carrier family 35 member C1
SLC35D2	solute carrier family 35 member D2
SLC35D3	solute carrier family 35 member D3
SLC35E1	solute carrier family 35 member E1
SLC35F1	solute carrier family 35 member F1
SLC35F3	solute carrier family 35 member F3
SLC35F5	solute carrier family 35 member F5
SLC35F6	solute carrier family 35 member F6
SLC35G2	solute carrier family 35 member G2
SLC36A1	solute carrier family 36 member 1
SLC36A4	solute carrier family 36 member 4
SLC38A1	solute carrier family 38 member 1
SLC38A2	solute carrier family 38 member 2
SLC38A7	solute carrier family 38 member 7
SLC39A10	solute carrier family 39 member 10
SLC39A14	solute carrier family 39 member 14
SLC39A6	solute carrier family 39 member 6
SLC39A9	solute carrier family 39 member 9
SLC41A1	solute carrier family 41 member 1
SLC41A2	solute carrier family 41 member 2
SLC44A1	solute carrier family 44 member 1
SLC45A4	solute carrier family 45 member 4
SLC46A3	solute carrier family 46 member 3
SLC4A1	solute carrier family 4 member 1 (Diego blood group)
SLC4A3	solute carrier family 4 member 3
SLC4A4	solute carrier family 4 member 4
SLC4A7	solute carrier family 4 member 7
SLC52A3	solute carrier family 52 member 3
SLC5A11	solute carrier family 5 member 11
SLC5A6	solute carrier family 5 member 6

SLC5A9	solute carrier family 5 member 9
SLC6A1	solute carrier family 6 member 1
SLC6A11	solute carrier family 6 member 11
SLC6A17	solute carrier family 6 member 17
SLC6A5	solute carrier family 6 member 5
SLC6A9	solute carrier family 6 member 9
SLC7A11	solute carrier family 7 member 11
SLC7A2	solute carrier family 7 member 2
SLC7A6	solute carrier family 7 member 6
SLC8A1	solute carrier family 8 member A1
SLC8A2	solute carrier family 8 member A2
SLC9A1	solute carrier family 9 member A1
SLC9A2	solute carrier family 9 member A2
SLCO5A1	solute carrier organic anion transporter family member 5A1
SLCO6A1	solute carrier organic anion transporter family member 6A1
SLFN12	schlafen family member 12
SLIT2	slit guidance ligand 2
SLITRK1	SLIT and NTRK like family member 1
SLITRK2	SLIT and NTRK like family member 2
SLK	STE20 like kinase
SLMAP	sarcolemma associated protein
SLTM	SAFB like transcription modulator
SLX1A	SLX1 homolog A, structure-specific endonuclease subunit
SLX1B	SLX1 homolog B, structure-specific endonuclease subunit
SMAD1	SMAD family member 1
SMAD2	SMAD family member 2
SMAD5	SMAD family member 5
SMAP1	small ArfGAP 1
SMAP2	small ArfGAP2
SMARCAD1	SWI/SNF-related, matrix-associated actin-dependent regulator of chromatin, subfamily a, containing DEAD/H box 1
SMARCB1	SWI/SNF related, matrix associated, actin dependent regulator of chromatin, subfamily b, member 1
SMARCC1	SWI/SNF related, matrix associated, actin dependent regulator of chromatin subfamily c member 1

SMARCC2	SWI/SNF related, matrix associated, actin dependent regulator of chromatin subfamily c member 2
SMC1A	structural maintenance of chromosomes 1A
SMCHD1	structural maintenance of chromosomes flexible hinge domain containing 1
SMDT1	single-pass membrane protein with aspartate rich tail 1
SMG1	SMG1, nonsense mediated mRNA decay associated PI3K related kinase
SMG7	SMG7, nonsense mediated mRNA decay factor
SMIM13	small integral membrane protein 13
SMIM14	small integral membrane protein 14
SMIM3	small integral membrane protein 3
SMIM5	small integral membrane protein 5
SMUG1	single-strand-selective monofunctional uracil-DNA glycosylase 1
SMURF1	SMAD specific E3 ubiquitin protein ligase 1
SNAI1	snail family transcriptional repressor 1
SNAI2	snail family transcriptional repressor 2
SNAP23	synaptosome associated protein 23
SNAP25	synaptosome associated protein 25
SNAP91	synaptosome associated protein 91
SNAPC1	small nuclear RNA activating complex polypeptide 1
SNAPIN	SNAP associated protein
SNN	stannin
SNTB2	syntrophin beta 2
SNTG1	syntrophin gamma 1
SNX1	sorting nexin 1
SNX10	sorting nexin 10
SNX12	sorting nexin 12
SNX16	sorting nexin 16
SNX18	sorting nexin 18
SNX19	sorting nexin 19
SNX20	sorting nexin 20
SNX25	sorting nexin 25
SNX27	sorting nexin family member 27
SNX29	sorting nexin 29

SNX3	sorting nexin 3
SNX30	sorting nexin family member 30
SNX33	sorting nexin 33
SNX6	sorting nexin 6
SOCS1	suppressor of cytokine signaling 1
SOCS3	suppressor of cytokine signaling 3
SOCS4	suppressor of cytokine signaling 4
SOCS6	suppressor of cytokine signaling 6
SON	SON DNA binding protein
SORBS2	sorbin and SH3 domain containing 2
SOS1	SOS Ras/Rac guanine nucleotide exchange factor 1
SOWAHC	sosondowah ankyrin repeat domain family member C
SOX1	SRY-box 1
SOX11	SRY-box 11
SOX12	SRY-box 12
SOX13	SRY-box 13
SOX2	SRY-box 2
SOX4	SRY-box 4
SOX9	SRY-box 9
SP2	Sp2 transcription factor
SP4	Sp4 transcription factor
SPAST	spastin
SPATA2L	spermatogenesis associated 2 like
SPATA5	spermatogenesis associated 5
SPATA7	spermatogenesis associated 7
SPATA8	spermatogenesis associated 8
SPATC1	spermatogenesis and centriole associated 1
SPATS2L	spermatogenesis associated serine rich 2 like
SPCS3	signal peptidase complex subunit 3
SPEF2	sperm flagellar 2
SPEG	SPEG complex locus
SPEN	spen family transcriptional repressor

SPESP1	sperm equatorial segment protein 1
SPHKAP	SPHK1 interactor, AKAP domain containing
SPIN1	spindlin 1
SPOCK3	SPARC (osteonectin), cwcv and kazal like domains proteoglycan 3
SPRED1	sprouty related EVH1 domain containing 1
SPRED2	sprouty related EVH1 domain containing 2
SPRYD4	SPRY domain containing 4
SPRYD7	SPRY domain containing 7
SPTBN1	spectrin beta, non-erythrocytic 1
SPTBN4	spectrin beta, non-erythrocytic 4
SPTLC3	serine palmitoyltransferase long chain base subunit 3
SPTSSA	serine palmitoyltransferase small subunit A
SPTY2D1	SPT2 chromatin protein domain containing 1
SQSTM1	sequestosome 1
SRD5A3	steroid 5 alpha-reductase 3
SREK1	splicing regulatory glutamic acid and lysine rich protein 1
SREK1IP1	SREK1 interacting protein 1
SRGAP3	SLIT-ROBO Rho GTPase activating protein 3
SRI	sorcin
SRL	sarcalumenin
SRP19	signal recognition particle 19
SRPK2	SRSF protein kinase 2
SRSF1	serine and arginine rich splicing factor 1
SRSF10	serine and arginine rich splicing factor 10
SRSF3	serine and arginine rich splicing factor 3
SRSF7	serine and arginine rich splicing factor 7
SRSF9	serine and arginine rich splicing factor 9
SRXN1	sulfiredoxin 1
SS18L1	SS18L1, nBAF chromatin remodeling complex subunit
SSBP2	single stranded DNA binding protein 2
SSFA2	
SSH1	slingshot protein phosphatase 1

SSH2	slingshot protein phosphatase 2
SSR3	signal sequence receptor subunit 3
SSX2IP	SSX family member 2 interacting protein
ST3GAL6	ST3 beta-galactoside alpha-2,3-sialyltransferase 6
ST6GALNAC3	ST6 N-acetylgalactosaminide alpha-2,6-sialyltransferase 3
ST8SIA1	ST8 alpha-N-acetyl-neuraminide alpha-2,8-sialyltransferase 1
ST8SIA2	ST8 alpha-N-acetyl-neuraminide alpha-2,8-sialyltransferase 2
ST8SIA4	ST8 alpha-N-acetyl-neuraminide alpha-2,8-sialyltransferase 4
STAC	SH3 and cysteine rich domain
STAM2	signal transducing adaptor molecule 2
STAP1	signal transducing adaptor family member 1
STARD13	StAR related lipid transfer domain containing 13
STARD3	StAR related lipid transfer domain containing 3
STARD3NL	STARD3 N-terminal like
STARD7	StAR related lipid transfer domain containing 7
STARD9	StAR related lipid transfer domain containing 9
STAT3	signal transducer and activator of transcription 3
STC1	stanniocalcin 1
STC2	stanniocalcin 2
STEAP3	STEAP3 metalloreductase
STIM2	stromal interaction molecule 2
STK11	serine/threonine kinase 11
STK17B	serine/threonine kinase 17b
STK19	serine/threonine kinase 19
STK24	serine/threonine kinase 24
STK32A	serine/threonine kinase 32A
STK35	serine/threonine kinase 35
STK39	serine/threonine kinase 39
STK4	serine/threonine kinase 4
STK40	serine/threonine kinase 40
STOX2	storkhead box 2
STRADB	STE20-related kinase adaptor beta

STRBP	spermatid perinuclear RNA binding protein
STRIP1	striatin interacting protein 1
STRN	striatin
STS	steroid sulfatase
STT3B	STT3B, catalytic subunit of the oligosaccharyltransferase complex
STX12	syntaxin 12
STX16	syntaxin 16
STX2	syntaxin 2
STX5	syntaxin 5
STX6	syntaxin 6
STYX	serine/threonine/tyrosine interacting protein
SUB1	SUB1 homolog, transcriptional regulator
SUGT1	SGT1 homolog, MIS12 kinetochore complex assembly cochaperone
SULF1	sulfatase 1
SULF2	sulfatase 2
SULT1A3	sulfotransferase family 1A member 3
SUPT20H	SPT20 homolog, SAGA complex component
SURF4	surfeit 4
SUSD3	sushi domain containing 3
SUSD5	sushi domain containing 5
SVIL	supervillin
SWT1	SWT1, RNA endoribonuclease homolog
SYBU	syntabulin
SYDE1	synapse defective Rho GTPase homolog 1
SYNC	syncoilin, intermediate filament protein
SYNCRIP	synaptotagmin binding cytoplasmic RNA interacting protein
SYNE1	spectrin repeat containing nuclear envelope protein 1
SYNGR3	synaptogyrin 3
SYNJ1	synaptojanin 1
SYPL1	synaptophysin like 1
SYT1	synaptotagmin 1
SYT10	synaptotagmin 10

SYT11	synaptotagmin 11
SYT2	synaptotagmin 2
SYT7	synaptotagmin 7
SYT9	synaptotagmin 9
SYTL4	synaptotagmin like 4
TAB2	TGF-beta activated kinase 1 (MAP3K7) binding protein 2
TAB3	TGF-beta activated kinase 1 (MAP3K7) binding protein 3
TACR1	tachykinin receptor 1
TACR3	tachykinin receptor 3
TAF12	TATA-box binding protein associated factor 12
TAF2	TATA-box binding protein associated factor 2
TAF4B	TATA-box binding protein associated factor 4b
TAF5	TATA-box binding protein associated factor 5
TAF5L	TATA-box binding protein associated factor 5 like
TAF9B	TATA-box binding protein associated factor 9b
TAGAP	T cell activation RhoGTPase activating protein
TAGLN2	transgelin 2
TANC2	tetratricopeptide repeat, ankyrin repeat and coiled-coil containing 2
TAOK1	TAO kinase 1
TAOK3	TAO kinase 3
TAP1	transporter 1, ATP binding cassette subfamily B member
TARBP2	TARBP2, RISC loading complex RNA binding subunit
TASP1	taspase 1
TAT	tyrosine aminotransferase
TBC1D10B	TBC1 domain family member 10B
TBC1D12	TBC1 domain family member 12
TBC1D13	TBC1 domain family member 13
TBC1D15	TBC1 domain family member 15
TBC1D22A	TBC1 domain family member 22A
TBC1D8B	TBC1 domain family member 8B
TBC1D9B	TBC1 domain family member 9B
TBCA	tubulin folding cofactor A

TBCC	tubulin folding cofactor C
TBCK	TBC1 domain containing kinase
TBK1	TANK binding kinase 1
TBKBP1	TBK1 binding protein 1
TBL1XR1	transducin beta like 1 X-linked receptor 1
TBPL1	TATA-box binding protein like 1
TBX18	T-box 18
TBX5	T-box 5
TC2N	tandem C2 domains, nuclear
TCAIM	T cell activation inhibitor, mitochondrial
TCF21	transcription factor 21
TCF24	transcription factor 24
TCF4	transcription factor 4
TDGF1	teratocarcinoma-derived growth factor 1
TDP1	tyrosyl-DNA phosphodiesterase 1
TDRD7	tudor domain containing 7
TDRP	testis development related protein
TEC	tec protein tyrosine kinase
TECPR2	tectonin beta-propeller repeat containing 2
TECRL	trans-2,3-enoyl-CoA reductase like
TERF1	telomeric repeat binding factor 1
TET1	tet methylcytosine dioxygenase 1
TET3	tet methylcytosine dioxygenase 3
TEX2	testis expressed 2
TEX22	testis expressed 22
TFAP2A	transcription factor AP-2 alpha
TFAP2B	transcription factor AP-2 beta
TFCP2L1	transcription factor CP2 like 1
TFDP1	transcription factor Dp-1
TFEC	transcription factor EC
TFR2	transferrin receptor 2
TGDS	TDP-glucose 4,6-dehydratase

TGFB1	transforming growth factor beta 1
TGFBR1	transforming growth factor beta receptor 1
TGFBR3	transforming growth factor beta receptor 3
TGIF1	TGFB induced factor homeobox 1
TGIF2	TGFB induced factor homeobox 2
TGOLN2	trans-golgi network protein 2
THAP1	THAP domain containing 1
THAP11	THAP domain containing 11
THAP2	THAP domain containing 2
THAP5	THAP domain containing 5
THBS1	thrombospondin 1
THEM4	thioesterase superfamily member 4
THOC2	THO complex 2
THRA	thyroid hormone receptor alpha
THRAP3	thyroid hormone receptor associated protein 3
THRB	thyroid hormone receptor beta
THRSP	thyroid hormone responsive
THSD7A	thrombospondin type 1 domain containing 7A
THUMPD3	THUMP domain containing 3
TIA1	TIA1 cytotoxic granule associated RNA binding protein
TICAM1	toll like receptor adaptor molecule 1
TIFA	TRAF interacting protein with forkhead associated domain
TIGD6	tigger transposable element derived 6
TIMM17A	translocase of inner mitochondrial membrane 17A
TIMP2	TIMP metallopeptidase inhibitor 2
TIMP3	TIMP metallopeptidase inhibitor 3
TJP1	tight junction protein 1
TK2	thymidine kinase 2, mitochondrial
TLDC1	TBC/LysM-Associated Domain Containing 1
TLE4	transducin like enhancer of split 4
TLK2	tousled like kinase 2
TLL1	tolloid like 1

TLL2	tolloid like 2
TLR4	toll like receptor 4
TM4SF18	transmembrane 4 L six family member 18
TM4SF20	transmembrane 4 L six family member 20
TM9SF4	transmembrane 9 superfamily member 4
TMA16	translation machinery associated 16 homolog
TMC7	transmembrane channel like 7
TMCC1	transmembrane and coiled-coil domain family 1
TMED10	transmembrane p24 trafficking protein 10
TMED2	transmembrane p24 trafficking protein 2
TMED4	transmembrane p24 trafficking protein 4
TMED5	transmembrane p24 trafficking protein 5
TMEFF1	transmembrane protein with EGF like and two follistatin like domains 1
TMEM106B	transmembrane protein 106B
TMEM123	transmembrane protein 123
TMEM129	transmembrane protein 129
TMEM135	transmembrane protein 135
TMEM143	transmembrane protein 143
TMEM144	transmembrane protein 144
TMEM154	transmembrane protein 154
TMEM167A	transmembrane protein 167A
TMEM169	transmembrane protein 169
TMEM17	transmembrane protein 17
TMEM170A	transmembrane protein 170A
TMEM170B	transmembrane protein 170B
TMEM178A	transmembrane protein 178A
TMEM178B	transmembrane protein 178B
TMEM181	transmembrane protein 181
TMEM184B	transmembrane protein 184B
TMEM189-UBE2V1	TMEM189-UBE2V1 readthrough
TMEM192	transmembrane protein 192
TMEM196	transmembrane protein 196

TMEM2	
TMEM211	transmembrane protein 211
TMEM222	transmembrane protein 222
TMEM229A	transmembrane protein 229A
TMEM229B	transmembrane protein 229B
TMEM232	transmembrane protein 232
TMEM243	transmembrane protein 243
TMEM246	transmembrane protein 246
TMEM254	transmembrane protein 254
TMEM255A	transmembrane protein 255A
TMEM26	transmembrane protein 26
TMEM260	transmembrane protein 260
TMEM33	transmembrane protein 33
TMEM41A	transmembrane protein 41A
TMEM47	transmembrane protein 47
TMEM51	transmembrane protein 51
TMEM56	transmembrane protein 56
TMEM64	transmembrane protein 64
TMEM65	transmembrane protein 65
TMEM68	transmembrane protein 68
TMEM69	transmembrane protein 69
TMEM87A	transmembrane protein 87A
TMEM98	transmembrane protein 98
TMOD2	tropomodulin 2
TMOD3	tropomodulin 3
TMPPE	transmembrane protein with metallophosphoesterase domain
TMPRSS11F	transmembrane serine protease 11F
TMTC1	transmembrane and tetratricopeptide repeat containing 1
TMTC2	transmembrane and tetratricopeptide repeat containing 2
TMTC3	transmembrane and tetratricopeptide repeat containing 3
TNFAIP8	TNF alpha induced protein 8
TNFAIP8L1	TNF alpha induced protein 8 like 1

TNFRSF10B	TNF receptor superfamily member 10b
TNFRSF10D	TNF receptor superfamily member 10d
TNFRSF11B	TNF receptor superfamily member 11b
TNFRSF1B	TNF receptor superfamily member 1B
TNFRSF9	TNF receptor superfamily member 9
TNFSF10	TNF superfamily member 10
TNFSF9	TNF superfamily member 9
TNIK	TRAF2 and NCK interacting kinase
TNKS	tankyrase
TNKS1BP1	tankyrase 1 binding protein 1
TNKS2	tankyrase 2
TNPO1	transportin 1
TNRC6A	trinucleotide repeat containing 6A
TNRC6B	trinucleotide repeat containing 6B
TNRC6C	trinucleotide repeat containing 6C
TNS4	tensin 4
TOB1	transducer of ERBB2, 1
TOMM20	translocase of outer mitochondrial membrane 20
TOPORS	TOP1 binding arginine/serine rich protein
TOR1AIP2	torsin 1A interacting protein 2
TOX3	TOX high mobility group box family member 3
TP53	tumor protein p53
TP53INP1	tumor protein p53 inducible nuclear protein 1
TP53TG3D	TP53 target 3D
TP73	tumor protein p73
TPBG	trophoblast glycoprotein
TPCN2	two pore segment channel 2
TPM2	tropomyosin 2
TPM3	tropomyosin 3
TPM4	tropomyosin 4
TPP1	tripeptidyl peptidase 1
TPPP	tubulin polymerization promoting protein

TPRX1	tetrapeptide repeat homeobox 1
TRABD	TraB domain containing
TRAF5	TNF receptor associated factor 5
TRAM2	translocation associated membrane protein 2
TRAPPC1	trafficking protein particle complex 1
TRAPPC2	trafficking protein particle complex 2
TRAPPC3	trafficking protein particle complex 3
TRAPPC6B	trafficking protein particle complex 6B
TRAPPC8	trafficking protein particle complex 8
TRDMT1	tRNA aspartic acid methyltransferase 1
TRHDE	thyrotropin releasing hormone degrading enzyme
TRIB1	tribbles pseudokinase 1
TRIB2	tribbles pseudokinase 2
TRIM13	tripartite motif containing 13
TRIM2	tripartite motif containing 2
TRIM23	tripartite motif containing 23
TRIM31	tripartite motif containing 31
TRIM33	tripartite motif containing 33
TRIM35	tripartite motif containing 35
TRIM37	tripartite motif containing 37
TRIM41	tripartite motif containing 41
TRIM59	tripartite motif containing 59
TRIM67	tripartite motif containing 67
TRIM71	tripartite motif containing 71
TRIML1	tripartite motif family like 1
TRIML2	tripartite motif family like 2
TRIQK	triple QxxK/R motif containing
TRMT10A	tRNA methyltransferase 10A
TRMT13	tRNA methyltransferase 13 homolog
TRMT5	tRNA methyltransferase 5
TRO	trophinin
TROVE2	TROVE domain family member 2

TRPA1	transient receptor potential cation channel subfamily A member 1
TRPC5	transient receptor potential cation channel subfamily C member 5
TRPC6	transient receptor potential cation channel subfamily C member 6
TRPM7	transient receptor potential cation channel subfamily M member 7
TRPS1	transcriptional repressor GATA binding 1
TRPV3	transient receptor potential cation channel subfamily V member 3
TSC1	TSC complex subunit 1
TSC22D1	TSC22 domain family member 1
TSC22D2	TSC22 domain family member 2
TSC22D3	TSC22 domain family member 3
TSEN15	tRNA splicing endonuclease subunit 15
TSG101	tumor susceptibility 101
TSHZ3	teashirt zinc finger homeobox 3
TSPAN13	tetraspanin 13
TSPAN18	tetraspanin 18
TSPAN2	tetraspanin 2
TSPAN4	tetraspanin 4
TSPAN9	tetraspanin 9
TSPEAR	thrombospondin type laminin G domain and EAR repeats
TSPYL4	TSPY like 4
TSTD2	thiosulfate sulfurtransferase like domain containing 2
TTC13	tetratricopeptide repeat domain 13
TTC14	tetratricopeptide repeat domain 14
TTC17	tetratricopeptide repeat domain 17
TTC21B	tetratricopeptide repeat domain 21B
TTC26	tetratricopeptide repeat domain 26
TTC31	tetratricopeptide repeat domain 31
TTC39C	tetratricopeptide repeat domain 39C
TTC8	tetratricopeptide repeat domain 8
TTC9	tetratricopeptide repeat domain 9
TTC9C	tetratricopeptide repeat domain 9C
TTL	tubulin tyrosine ligase

TTLL4	tubulin tyrosine ligase like 4
TTLL5	tubulin tyrosine ligase like 5
TTLL6	tubulin tyrosine ligase like 6
TTLL7	tubulin tyrosine ligase like 7
TTPA	alpha tocopherol transfer protein
TTPAL	alpha tocopherol transfer protein like
TTR	transthyretin
TUBB6	tubulin beta 6 class V
TUBGCP3	tubulin gamma complex associated protein 3
TUBGCP6	tubulin gamma complex associated protein 6
TUSC2	tumor suppressor 2, mitochondrial calcium regulator
TUSC3	tumor suppressor candidate 3
TVP23B	trans-golgi network vesicle protein 23 homolog B
TWF1	twinfilin actin binding protein 1
TWISTNB	TWIST neighbor
TWSG1	twisted gastrulation BMP signaling modulator 1
TXLNG	taxilin gamma
TYW5	tRNA-yW synthesizing protein 5
UACA	uveal autoantigen with coiled-coil domains and ankyrin repeats
UAP1	UDP-N-acetylglucosamine pyrophosphorylase 1
UBA5	ubiquitin like modifier activating enzyme 5
UBA6	ubiquitin like modifier activating enzyme 6
UBAC1	UBA domain containing 1
UBE2B	ubiquitin conjugating enzyme E2 B
UBE2D1	ubiquitin conjugating enzyme E2 D1
UBE2D2	ubiquitin conjugating enzyme E2 D2
UBE2D3	ubiquitin conjugating enzyme E2 D3
UBE2F	ubiquitin conjugating enzyme E2 F (putative)
UBE2G1	ubiquitin conjugating enzyme E2 G1
UBE2H	ubiquitin conjugating enzyme E2 H
UBE2I	ubiquitin conjugating enzyme E2 I
UBE2J1	ubiquitin conjugating enzyme E2 J1

UBE2K	ubiquitin conjugating enzyme E2 K
UBE2R2	ubiquitin conjugating enzyme E2 R2
UBE2V1	ubiquitin conjugating enzyme E2 V1
UBE2V2	ubiquitin conjugating enzyme E2 V2
UBE2W	ubiquitin conjugating enzyme E2 W
UBE3A	ubiquitin protein ligase E3A
UBE3C	ubiquitin protein ligase E3C
UBE4A	ubiquitination factor E4A
UBN1	ubinuclein 1
UBN2	ubinuclein 2
UBP1	upstream binding protein 1
UBR5	ubiquitin protein ligase E3 component n-recognin 5
UBTD2	ubiquitin domain containing 2
UBXN10	UBX domain protein 10
UBXN2A	UBX domain protein 2A
UBXN2B	UBX domain protein 2B
UBXN7	UBX domain protein 7
UCHL5	ubiquitin C-terminal hydrolase L5
UCP3	uncoupling protein 3
UFL1	UFM1 specific ligase 1
UFM1	ubiquitin fold modifier 1
UGCG	UDP-glucose ceramide glucosyltransferase
UGGT1	UDP-glucose glycoprotein glucosyltransferase 1
UGT2A3	UDP glucuronosyltransferase family 2 member A3
UGT3A1	UDP glycosyltransferase family 3 member A1
UGT8	UDP glycosyltransferase 8
UHMK1	U2AF homology motif kinase 1
UHRF1BP1	UHRF1 binding protein 1
UHRF2	ubiquitin like with PHD and ring finger domains 2
ULK1	unc-51 like autophagy activating kinase 1
ULK2	unc-51 like autophagy activating kinase 2
ULK3	unc-51 like kinase 3

UNC119	unc-119 lipid binding chaperone
UNC119B	unc-119 lipid binding chaperone B
UNC50	unc-50 inner nuclear membrane RNA binding protein
UNKL	unkempt family like zinc finger
UPRT	uracil phosphoribosyltransferase homolog
USP12	ubiquitin specific peptidase 12
USP13	ubiquitin specific peptidase 13
USP15	ubiquitin specific peptidase 15
USP18	ubiquitin specific peptidase 18
USP2	ubiquitin specific peptidase 2
USP21	ubiquitin specific peptidase 21
USP22	ubiquitin specific peptidase 22
USP24	ubiquitin specific peptidase 24
USP25	ubiquitin specific peptidase 25
USP28	ubiquitin specific peptidase 28
USP3	ubiquitin specific peptidase 3
USP33	ubiquitin specific peptidase 33
USP37	ubiquitin specific peptidase 37
USP38	ubiquitin specific peptidase 38
USP44	ubiquitin specific peptidase 44
USP45	ubiquitin specific peptidase 45
USP46	ubiquitin specific peptidase 46
USP47	ubiquitin specific peptidase 47
USP48	ubiquitin specific peptidase 48
USP49	ubiquitin specific peptidase 49
USP6	ubiquitin specific peptidase 6
USP6NL	USP6 N-terminal like
USP9X	ubiquitin specific peptidase 9 X-linked
UST	uronyl 2-sulfotransferase
UTRN	utrophin
VAMP1	vesicle associated membrane protein 1
VAMP3	vesicle associated membrane protein 3

VAMP4	vesicle associated membrane protein 4
VANGL2	VANGL planar cell polarity protein 2
VAPA	VAMP associated protein A
VASH1	vasohibin 1
VASH2	vasohibin 2
VAT1	vesicle amine transport 1
VAT1L	vesicle amine transport 1 like
VAV3	vav guanine nucleotide exchange factor 3
VCPIP1	valosin containing protein interacting protein 1
VDAC1	voltage dependent anion channel 1
VEGFA	vascular endothelial growth factor A
VENTX	VENT homeobox
VEZT	vezatin, adherens junctions transmembrane protein
VGLL3	vestigial like family member 3
VGLL4	vestigial like family member 4
VIM	vimentin
VKORC1L1	vitamin K epoxide reductase complex subunit 1 like 1
VLDLR	very low density lipoprotein receptor
VOPP1	VOPP1, WBP1/VOPP1 family member
VPS13A	vacuolar protein sorting 13 homolog A
VPS13C	vacuolar protein sorting 13 homolog C
VPS25	vacuolar protein sorting 25 homolog
VPS26A	VPS26, retromer complex component A
VPS26B	VPS26, retromer complex component B
VPS33A	VPS33A, CORVET/HOPS core subunit
VSIG1	V-set and immunoglobulin domain containing 1
VSTM5	V-set and transmembrane domain containing 5
VTI1B	vesicle transport through interaction with t-SNAREs 1B
VWA3B	von Willebrand factor A domain containing 3B
WARS2	tryptophanyl tRNA synthetase 2, mitochondrial
WASF3	WAS protein family member 3
WASL	Wiskott-Aldrich syndrome like

WBP1L	WW domain binding protein 1 like
WDR1	WD repeat domain 1
WDR20	WD repeat domain 20
WDR26	WD repeat domain 26
WDR33	WD repeat domain 33
WDR35	WD repeat domain 35
WDR37	WD repeat domain 37
WDR44	WD repeat domain 44
WDR48	WD repeat domain 48
WDR7	WD repeat domain 7
WDR73	WD repeat domain 73
WDR82	WD repeat domain 82
WFDC3	WAP four-disulfide core domain 3
WIF1	WNT inhibitory factor 1
WIPF2	WAS/WASL interacting protein family member 2
WIPF3	WAS/WASL interacting protein family member 3
WIPI2	WD repeat domain, phosphoinositide interacting 2
WNK3	WNK lysine deficient protein kinase 3
WNT16	Wnt family member 16
WNT2B	Wnt family member 2B
WNT5A	Wnt family member 5A
WNT8B	Wnt family member 8B
WNT9A	Wnt family member 9A
WT1	Wilms tumor 1
WWC2	WW and C2 domain containing 2
WWC3	WWC family member 3
WWP1	WW domain containing E3 ubiquitin protein ligase 1
XAF1	XIAP associated factor 1
XIAP	X-linked inhibitor of apoptosis
XK	X-linked Kx blood group
XKR4	XK related 4
XKR6	XK related 6

XKR8	XK related 8
XPNPEP3	X-prolyl aminopeptidase 3
XPO1	exportin 1
XPO4	exportin 4
XPO6	exportin 6
XPOT	exportin for tRNA
XPR1	xenotropic and polytropic retrovirus receptor 1
XRN1	5'-3' exoribonuclease 1
XYLT1	xylosyltransferase 1
XYLT2	xylosyltransferase 2
YAF2	YY1 associated factor 2
YAP1	Yes associated protein 1
YBX1	Y-box binding protein 1
YBX3	Y-box binding protein 3
YES1	YES proto-oncogene 1, Src family tyrosine kinase
YIPF1	Yip1 domain family member 1
YIPF3	Yip1 domain family member 3
YLPM1	YLP motif containing 1
YOD1	YOD1 deubiquitinase
YPEL2	yippee like 2
YPEL5	yippee like 5
YTHDC1	YTH domain containing 1
YWHAE	tyrosine 3-monooxygenase/tryptophan 5-monooxygenase activation protein epsilon
YWHAG	tyrosine 3-monooxygenase/tryptophan 5-monooxygenase activation protein gamma
YWHAQ	tyrosine 3-monooxygenase/tryptophan 5-monooxygenase activation protein theta
YWHAZ	tyrosine 3-monooxygenase/tryptophan 5-monooxygenase activation protein zeta
YY1	YY1 transcription factor
YY2	YY2 transcription factor
ZBTB10	zinc finger and BTB domain containing 10
ZBTB11	zinc finger and BTB domain containing 11
ZBTB18	zinc finger and BTB domain containing 18
ZBTB21	zinc finger and BTB domain containing 21

ZBTB24	zinc finger and BTB domain containing 24
ZBTB26	zinc finger and BTB domain containing 26
ZBTB33	zinc finger and BTB domain containing 33
ZBTB37	zinc finger and BTB domain containing 37
ZBTB39	zinc finger and BTB domain containing 39
ZBTB4	zinc finger and BTB domain containing 4
ZBTB40	zinc finger and BTB domain containing 40
ZBTB41	zinc finger and BTB domain containing 41
ZBTB49	zinc finger and BTB domain containing 49
ZBTB5	zinc finger and BTB domain containing 5
ZBTB7A	zinc finger and BTB domain containing 7A
ZBTB7C	zinc finger and BTB domain containing 7C
ZBTB8A	zinc finger and BTB domain containing 8A
ZBTB8B	zinc finger and BTB domain containing 8B
ZC2HC1C	zinc finger C2HC-type containing 1C
ZC3H11A	zinc finger CCCH-type containing 11A
ZC3H3	zinc finger CCCH-type containing 3
ZC3H6	zinc finger CCCH-type containing 6
ZC3H7B	zinc finger CCCH-type containing 7B
ZC3H8	zinc finger CCCH-type containing 8
ZCCHC11	
ZCCHC14	zinc finger CCHC-type containing 14
ZCCHC24	zinc finger CCHC-type containing 24
ZCCHC9	zinc finger CCHC-type containing 9
ZCRB1	zinc finger CCHC-type and RNA binding motif containing 1
ZDHHC15	zinc finger DHHC-type containing 15
ZDHHC17	zinc finger DHHC-type containing 17
ZDHHC21	zinc finger DHHC-type containing 21
ZDHHC6	zinc finger DHHC-type containing 6
ZDHHC9	zinc finger DHHC-type containing 9
ZEB1	zinc finger E-box binding homeobox 1
ZEB2	zinc finger E-box binding homeobox 2

ZFAND5	zinc finger AN1-type containing 5
ZFAND6	zinc finger AN1-type containing 6
ZFC3H1	zinc finger C3H1-type containing
ZFHX2	zinc finger homeobox 2
ZFHX4	zinc finger homeobox 4
ZFP1	ZFP1 zinc finger protein
ZFP3	ZFP3 zinc finger protein
ZFP36L1	ZFP36 ring finger protein like 1
ZFP36L2	ZFP36 ring finger protein like 2
ZFPM2	zinc finger protein, FOG family member 2
ZFX	zinc finger protein X-linked
ZFY	zinc finger protein Y-linked
ZFYVE16	zinc finger FYVE-type containing 16
ZFYVE26	zinc finger FYVE-type containing 26
ZIC1	Zic family member 1
ZIC5	Zic family member 5
ZKSCAN2	zinc finger with KRAB and SCAN domains 2
ZKSCAN8	zinc finger with KRAB and SCAN domains 8
ZMAT3	zinc finger matrin-type 3
ZMYND11	zinc finger MYND-type containing 11
ZNF10	zinc finger protein 10
ZNF100	zinc finger protein 100
ZNF117	zinc finger protein 117
ZNF12	zinc finger protein 12
ZNF124	zinc finger protein 124
ZNF141	zinc finger protein 141
ZNF148	zinc finger protein 148
ZNF154	zinc finger protein 154
ZNF197	zinc finger protein 197
ZNF200	zinc finger protein 200
ZNF202	zinc finger protein 202
ZNF217	zinc finger protein 217

ZNF226	zinc finger protein 226
ZNF227	zinc finger protein 227
ZNF235	zinc finger protein 235
ZNF24	zinc finger protein 24
ZNF250	zinc finger protein 250
ZNF260	zinc finger protein 260
ZNF268	zinc finger protein 268
ZNF275	zinc finger protein 275
ZNF280C	zinc finger protein 280C
ZNF281	zinc finger protein 281
ZNF286B	zinc finger protein 286B
ZNF302	zinc finger protein 302
ZNF318	zinc finger protein 318
ZNF326	zinc finger protein 326
ZNF329	zinc finger protein 329
ZNF331	zinc finger protein 331
ZNF341	zinc finger protein 341
ZNF343	zinc finger protein 343
ZNF345	zinc finger protein 345
ZNF347	zinc finger protein 347
ZNF354B	zinc finger protein 354B
ZNF365	zinc finger protein 365
ZNF367	zinc finger protein 367
ZNF382	zinc finger protein 382
ZNF385B	zinc finger protein 385B
ZNF394	zinc finger protein 394
ZNF395	zinc finger protein 395
ZNF410	zinc finger protein 410
ZNF423	zinc finger protein 423
ZNF429	zinc finger protein 429
ZNF430	zinc finger protein 430
ZNF431	zinc finger protein 431

ZNF436	zinc finger protein 436
ZNF44	zinc finger protein 44
ZNF451	zinc finger protein 451
ZNF461	zinc finger protein 461
ZNF462	zinc finger protein 462
ZNF471	zinc finger protein 471
ZNF473	zinc finger protein 473
ZNF474	zinc finger protein 474
ZNF484	zinc finger protein 484
ZNF512	zinc finger protein 512
ZNF516	zinc finger protein 516
ZNF521	zinc finger protein 521
ZNF527	zinc finger protein 527
ZNF529	zinc finger protein 529
ZNF532	zinc finger protein 532
ZNF543	zinc finger protein 543
ZNF548	zinc finger protein 548
ZNF550	zinc finger protein 550
ZNF557	zinc finger protein 557
ZNF566	zinc finger protein 566
ZNF577	zinc finger protein 577
ZNF580	zinc finger protein 580
ZNF582	zinc finger protein 582
ZNF583	zinc finger protein 583
ZNF585A	zinc finger protein 585A
ZNF592	zinc finger protein 592
ZNF606	zinc finger protein 606
ZNF618	zinc finger protein 618
ZNF620	zinc finger protein 620
ZNF621	zinc finger protein 621
ZNF644	zinc finger protein 644
ZNF652	zinc finger protein 652

ZNF655	zinc finger protein 655
ZNF677	zinc finger protein 677
ZNF679	zinc finger protein 679
ZNF689	zinc finger protein 689
ZNF692	zinc finger protein 692
ZNF697	zinc finger protein 697
ZNF704	zinc finger protein 704
ZNF708	zinc finger protein 708
ZNF710	zinc finger protein 710
ZNF711	zinc finger protein 711
ZNF763	zinc finger protein 763
ZNF772	zinc finger protein 772
ZNF773	zinc finger protein 773
ZNF774	zinc finger protein 774
ZNF780A	zinc finger protein 780A
ZNF781	zinc finger protein 781
ZNF784	zinc finger protein 784
ZNF789	zinc finger protein 789
ZNF792	zinc finger protein 792
ZNF800	zinc finger protein 800
ZNF827	zinc finger protein 827
ZNF83	zinc finger protein 83
ZNF831	zinc finger protein 831
ZNF843	zinc finger protein 843
ZNF850	zinc finger protein 850
ZNF879	zinc finger protein 879
ZNFX1	zinc finger NFX1-type containing 1
ZNRF1	zinc and ring finger 1
ZNRF3	zinc and ring finger 3
ZPBP2	zona pellucida binding protein 2
ZPLD1	zona pellucida like domain containing 1
ZRANB2	zinc finger RANBP2-type containing 2

ZSCAN29	zinc finger and SCAN domain containing 29
ZSWIM4	zinc finger SWIM-type containing 4
ZSWIM5	zinc finger SWIM-type containing 5
ZXDA	zinc finger X-linked duplicated A
ZZZ3	zinc finger ZZ-type containing 3
