# Supplementary material for: The Interaction between 30b-5p miRNA and MBNL1 mRNA is Involved in Vascular Smooth Muscle Cell Differentiation in Patients with Coronary Atherosclerosis
Source: Int J Mol Sci. 2019 Dec 18;21(1):11. doi: 10.3390/ijms21010011 (PMC6982107; doi:10.3390/ijms21010011)
Supplement: Supplementary file 1 [file ijms-21-00011-s001.zip › Supplementary Table S2.pdf]

Supplementary Table 2: The common predicted target genes obtained from the 3 databases and differentially expressed genes from microarray study were selected for further analysis.

Gene Symbol	Description
AASDHPPT	Aminoadipate-Semialdehyde Dehydrogenase-Phosphopantetheinyl Transferase
ACADM	Acyl-CoA Dehydrogenase Medium Chain
ACSL3	Acyl-CoA Synthetase Long Chain Family Member 3
ADAM22	ADAM Metallopeptidase Domain 22
ADAM28	ADAM Metallopeptidase Domain 28
ADAMTS5	ADAM Metallopeptidase With Thrombospondin Type 1 Motif 5
ADD3	Adducin 3
AGFG2	ArfGAP With FG Repeats 2
AHCTF1	AT-Hook Containing Transcription Factor 1
AKAP11	A-Kinase Anchoring Protein 11
AKAP6	A-Kinase Anchoring Protein 6
AKR1B10	Aldo-Keto Reductase Family 1 Member B10
AKTIP	AKT Interacting Protein
ANKRD12	Ankyrin Repeat Domain 12
ANKRD17	Ankyrin Repeat Domain 17
API5	Apoptosis Inhibitor 5
ARFIP1	ADP Ribosylation Factor Interacting Protein 1
ARGLU1	Arginine And Glutamate Rich 1
ARHGAP21	Rho GTPase Activating Protein 21
ARID2	AT-Rich Interaction Domain 2
ARL2BP	ADP Ribosylation Factor Like GTPase 2 Binding Protein
ARL5A	ADP Ribosylation Factor Like GTPase 5A
ARL5B	ADP Ribosylation Factor Like GTPase 5B
ASAH2B	N-Acylsphingosine Amidohydrolase 2B
ASXL2	ASXL Transcriptional Regulator 2
ATP1A2	ATPase Na+/K+ Transporting Subunit Alpha 2
ATP2B3	ATPase Plasma Membrane Ca2+ Transporting 3
AXL	AXL Receptor Tyrosine Kinase
BAG4	BCL2 Associated Athanogene 4
BASP1	Brain Abundant Membrane Attached Signal Protein 1
BECN1	Beclin 1
BRCC3	BRCA1/BRCA2-Containing Complex Subunit 3
BTBD10	BTB Domain Containing 10
BZW1	Basic Leucine Zipper And W2 Domains 1
C5orf51	Chromosome 5 Open Reading Frame 51
CACNB2	Calcium Voltage-Gated Channel Auxiliary Subunit Beta 2
CADM2	Cell Adhesion Molecule 2
CAMSAP2	Calmodulin Regulated Spectrin Associated Protein Family Member 2
CAMTA1	Calmodulin Binding Transcription Activator 1
CAPRIN1	Cell Cycle Associated Protein 1
CBX3	Chromobox 3
CCDC148	Coiled-Coil Domain Containing 148
CCDC68	Coiled-Coil Domain Containing 68
CCDC7	Coiled-Coil Domain Containing 7
CCNJ	Cyclin J
CD55	CD55 Molecule (Cromer Blood Group)
CDC40	Cell Division Cycle 40
CDH1	Cadherin 1
CEP128	Centrosomal Protein 128
CEP350	Centrosomal Protein 350
CFHR3	Complement Factor H Related 3
CHN2	Chimerin 2
CHSY1	Chondroitin Sulfate Synthase 1
CNOT7	CCR4-NOT Transcription Complex Subunit 7
CNTNAP3	Contactin Associated Protein Like 3
COL11A1	Collagen Type XI Alpha 1 Chain
COL4A3BP	Collagen Type IV Alpha 3 Binding Protein
COL5A2	Collagen Type V Alpha 2 Chain
CPEB3	Cytoplasmic Polyadenylation Element Binding Protein 3
CREB1	CAMP Responsive Element Binding Protein 1
CREBZF	CREB/ATF BZIP Transcription Factor
CRKL	CRK Like Proto-Oncogene, Adaptor Protein
CTBP2	C-Terminal Binding Protein 2
CTDSP2	CTD Small Phosphatase 2
CTNS	Cystinosin, Lysosomal Cystine Transporter
CTPS2	CTP Synthase 2
CXXC4	CXXC Finger Protein 4
CYLC2	Cylicin 2
DCAF4L2	DB1 And CUL4 Associated Factor 4 Like 2

DCUN1D1	Defective In Cullin Neddylation 1 Domain Containing 1
DDX5	DEAD-Box Helicase 5
DEXI	Dexi Homolog
DHX36	DEAH-Box Helicase 36
DICER1	Dicer 1, Ribonuclease III
DLC1	DLC1 Rho GTPase Activating Protein
DLST	Dihydrolipoamide S-Succinyltransferase
DNASE2B	Deoxyribonuclease 2 Beta
DOCK7	Dedicator Of Cytokinesis 7
DST	Dystonin
DYRK2	Dual Specificity Tyrosine Phosphorylation Regulated Kinase 2
EDEM3	ER Degradation Enhancing Alpha-Mannosidase Like Protein 3
ELAVL3	ELAV Like RNA Binding Protein 3
ENAH	ENAH, Actin Regulator
ENY2	ENY2, Transcription And Export Complex 2 Subunit
EPDR1	Ependymin Related 1
EPHA5	EPH Receptor A5
EREG	Epiregulin
ERG	ERG, ETS Transcription Factor
ETV1	ETS Variant 1
FAIM	Fas Apoptotic Inhibitory Molecule
FAM107B	Family With Sequence Similarity 107 Member B
FAM126B	Family With Sequence Similarity 126 Member B
FAM160B1	Family With Sequence Similarity 160 Member B1
FAM19A2	amily With Sequence Similarity 19 Member A2, C-C Motif Chemokine Like
FAM210B	Family With Sequence Similarity 210 Member B
FAM218A	Family With Sequence Similarity 218 Member A
FAM8A1	Family With Sequence Similarity 8 Member A1
FBXW11	F-Box And WD Repeat Domain Containing 11
FBXW7	F-Box And WD Repeat Domain Containing 7
FOXO3	Forkhead Box O3
FOXP1	Forkhead Box P1
FSD1L	Fibronectin Type III And SPRY Domain Containing 1 Like
GIT2	GIT ArfGAP 2
GNAQ	G Protein Subunit Alpha Q
GNPNAT1	Glucosamine-Phosphate N-Acetyltransferase 1
GOLPH3L	Golgi Phosphoprotein 3 Like
GOSR1	Golgi SNAP Receptor Complex Member 1
GPALPP1	GPALPP Motifs Containing 1
GPD2	Glycerol-3-Phosphate Dehydrogenase 2
GYG2	Glycogenin 2
GYPE	Glycophorin E (MNS Blood Group)
HIF1A	Hypoxia Inducible Factor 1 Subunit Alpha
HMGN1	High Mobility Group Nucleosome Binding Domain 1
HNRNPA1	Heterogeneous Nuclear Ribonucleoprotein A1
HNRNPC	Heterogeneous Nuclear Ribonucleoprotein C (C1/C2)
HNRNPU	Heterogeneous Nuclear Ribonucleoprotein U
HOMER1	Homer Scaffold Protein 1
HOOK1	Hook Microtubule Tethering Protein 1
HSPA9	Heat Shock Protein Family A (Hsp70) Member 9
HSPD1	Heat Shock Protein Family D (Hsp60) Member 1
HTR1F	5-Hydroxytryptamine Receptor 1F
IGF1	Insulin Like Growth Factor 1
IGF2BP3	Insulin Like Growth Factor 2 MRNA Binding Protein 3
ITCH	Itchy E3 Ubiquitin Protein Ligase
ITFG2	Integrin Alpha FG-GAP Repeat Containing 2
ITGB1	Integrin Subunit Beta 1
KCNMB2	Potassium Calcium-Activated Channel Subfamily M Regulatory Beta Subunit 2
KCTD16	Potassium Channel Tetramerization Domain Containing 16
KCTD9	Potassium Channel Tetramerization Domain Containing 9
KDM3A	Lysine Demethylase 3A
KLF3	Kruppel Like Factor 3
KLF4	Kruppel Like Factor 4
KLHL31	Kelch Like Family Member 31
KLHL42	Kelch Like Family Member 42
KPNA4	Karyopherin Subunit Alpha 4
LAPTM4A	Lysosomal Protein Transmembrane 4 Alpha
LCOR	Ligand Dependent Nuclear Receptor Corepressor
LDHC	Lactate Dehydrogenase C
LEMD2	LEM Domain Containing 2
LHFPL2	LHFPL Tetraspan Subfamily Member 2
LIMCH1	LIM And Calponin Homology Domains 1
LIMS1	LIM Zinc Finger Domain Containing 1

LIPI	Lipase I
LMO3	LIM Domain Only 3
LOX	Lysyl Oxidase
LYRM7	LYR Motif Containing 7
MAP1B	Microtubule Associated Protein 1B
MAP3K1	Mitogen-Activated Protein Kinase Kinase Kinase 1
MAP3K2	Mitogen-Activated Protein Kinase Kinase Kinase 2
MAPK8	Mitogen-Activated Protein Kinase 8
MASP1	Mannan Binding Lectin Serine Peptidase 1
MATR3	Matrin 3
MBNL1	Muscleblind Like Splicing Regulator 1
MEF2D	Myocyte Enhancer Factor 2D
MIER1	MIER1 Transcriptional Regulator
MMP2	Matrix Metallopeptidase 2
MOB3B	MOB Kinase Activator 3B
MRO	Maestro
MTF2	Metal Response Element Binding Transcription Factor 2
MTTP	Microsomal Triglyceride Transfer Protein
MXI1	MAX Interactor 1, Dimerization Protein
MYOCD	Myocardin
NAA50	N(Alpha)-Acetyltransferase 50, NatE Catalytic Subunit
NAALADL2	N-Acetylated Alpha-Linked Acidic Dipeptidase Like 2
NANP	N-Acetylneuraminic Acid Phosphatase
NAP1L1	Nucleosome Assembly Protein 1 Like 1
NCOA7	Nuclear Receptor Coactivator 7
NEDD1	Neural Precursor Cell Expressed, Developmentally Down-Regulated 1
NEK7	NIMA Related Kinase 7
NET1	Neuroepithelial Cell Transforming 1
NFATC3	Nuclear Factor Of Activated T Cells 3
NFIB	Nuclear Factor I B
NHLRC2	NHL Repeat Containing 2
NLN	Neurolysin
NME9	NME/NM23 Family Member 9
NOVA1	NOVA Alternative Splicing Regulator 1
NR1D2	Nuclear Receptor Subfamily 1 Group D Member 2
OARD1	O-Acyl-ADP-Ribose Deacylase 1
P4HA1	Prolyl 4-Hydroxylase Subunit Alpha 1
PAG1	Phosphoprotein Membrane Anchor With Glycosphingolipid Microdomains 1
PAIP2	Poly(A) Binding Protein Interacting Protein 2
PAPD5	Terminal Nucleotidyltransferase 4B
PAPPA	Pappalysin 1
PBX1	PBX Homeobox 1
PBX2	PBX Homeobox 2
PCDH17	Protocadherin 17
PCMTD2	Protein-L-Isoaspartate (D-Aspartate) O-Methyltransferase Domain Containing 2
PDGFRA	Platelet Derived Growth Factor Receptor Alpha
PFDN1	Prefoldin Subunit 1
PFN2	Profilin 2
PKD2	Polycystin 2, Transient Receptor Potential Cation Channel
PLAG1	PLAG1 Zinc Finger
PLEKHA3	Pleckstrin Homology Domain Containing A3
PLEKHB2	Pleckstrin Homology Domain Containing B2
PNRC2	Proline Rich Nuclear Receptor Coactivator 2
POLR3G	RNA Polymerase III Subunit G
PPHLN1	Periphilin 1
PPP1CB	Protein Phosphatase 1 Catalytic Subunit Beta
PPP4R2	Protein Phosphatase 4 Regulatory Subunit 2
PPTC7	PTC7 Protein Phosphatase Homolog
PRKAA1	Protein Kinase AMP-Activated Catalytic Subunit Alpha 1
PRPF40A	Pre-MRNA Processing Factor 40 Homolog A
PRTG	Protogenin
PTCHD4	Patched Domain Containing 4
PTP4A1	Protein Tyrosine Phosphatase Type IVA, Member 1
RANBP2	RAN Binding Protein 2
RBBP9	RB Binding Protein 9, Serine Hydrolase
RBFOX1	RNA Binding Fox-1 Homolog 1
RBM12B	RNA Binding Motif Protein 12B
RDX	Radixin
RFX6	Regulatory Factor X6
RIMKLB	Ribosomal Modification Protein RimK Like Family Member B
RNASEH2C	Ribonuclease H2 Subunit C
RNF122	Ring Finger Protein 122
RNFT1	Ring Finger Protein, Transmembrane 1

RPS6KA6	Ribosomal Protein S6 Kinase A6
RPS6KB1	Ribosomal Protein S6 Kinase B1
RRAGD	Ras Related GTP Binding D
RSBN1L	Round Spermatid Basic Protein 1 Like
RUFY2	RUN And FYVE Domain Containing 2
RUFY3	RUN And FYVE Domain Containing 3
SECISBP2L	SECIS Binding Protein 2 Like
SEMA3A	Semaphorin 3A
SERBP1	SERPINE1 MRNA Binding Protein 1
SH3YL1	SH3 And SYLF Domain Containing 1
SKA2	Spindle And Kinetochore Associated Complex Subunit 2
SKIL	SKI Like Proto-Oncogene
SLC36A1	Solute Carrier Family 36 Member 1
SLC4A4	Solute Carrier Family 4 Member 4
SLC4A7	Solute Carrier Family 4 Member 7
SLC7A2	Solute Carrier Family 7 Member 2
SMAP1	Small ArfGAP 1
SMARCAD1	SWI/SNF-Related, Matrix-Associated Actin-Dependent Regulator Of Chromatin, Subfamily A, Containing DEAD/H Box 1
SNX10	Sorting Nexin 10
SNX27	Sorting Nexin Family Member 27
SNX30	Sorting Nexin Family Member 30
SOCS3	Suppressor Of Cytokine Signaling 3
SOX13	SRY-Box 13
SPATA5	Spermatogenesis Associated 5
SPATS2L	Spermatogenesis Associated Serine Rich 2 Like
SRSF10	Serine And Arginine Rich Splicing Factor 10
SSBP2	Single Stranded DNA Binding Protein 2
SSR3	Signal Sequence Receptor Subunit 3
STYX	Serine/Threonine/Tyrosine Interacting Protein
SYNCRIP	Synaptotagmin Binding Cytoplasmic RNA Interacting Protein
TACR3	Tachykinin Receptor 3
TBL1XR1	Transducin Beta Like 1 X-Linked Receptor 1
TCF4	Transcription Factor 4
TDGF1	Teratocarcinoma-Derived Growth Factor 1
THRA	Thyroid Hormone Receptor Alpha
THRB	Thyroid Hormone Receptor Beta
TMEM106B	Transmembrane Protein 106B
TMEM154	Transmembrane Protein 154
TPM4	Tropomyosin 4
TPP1	Tripeptidyl Peptidase 1
TRAPPC6B	Trafficking Protein Particle Complex 6B
TROVE2	TROVE Domain Family Member 2
TSHZ3	Teashirt Zinc Finger Homeobox 3
TTC14	Tetratricopeptide Repeat Domain 14
TTC39C	Tetratricopeptide Repeat Domain 39C
TTLL5	Tubulin Tyrosine Ligase Like 5
TWF1	Twinfilin Actin Binding Protein 1
UACA	Uveal Autoantigen With Coiled-Coil Domains And Ankyrin Repeats
UBE2D1	Ubiquitin Conjugating Enzyme E2 D1
UBE2K	Ubiquitin Conjugating Enzyme E2 K
UBE3A	Ubiquitin Protein Ligase E3A
UFM1	Ubiquitin Fold Modifier 1
VAMP4	Vesicle Associated Membrane Protein 4
YWHAZ	Tyrosine 3-Monooxygenase/Tryptophan 5-Monooxygenase Activation Protein Zeta
YY1	YY1 Transcription Factor
ZEB2	Zinc Finger E-Box Binding Homeobox 2
ZNF429	Zinc Finger Protein 429
ZNF557	Zinc Finger Protein 557
ZNF655	Zinc Finger Protein 655
ZNF677	Zinc Finger Protein 677
ZNF781	Zinc Finger Protein 781
